# A Comprehensive Prescription for Plant miRNA Identification

**DOI:** 10.3389/fpls.2016.02058

**Published:** 2017-01-24

**Authors:** Burcu Alptekin, Bala A. Akpinar, Hikmet Budak

**Affiliations:** ^1^Cereal Genomics Lab, Department of Plant Sciences and Plant Pathology, Montana State UniversityBozeman, MT, USA; ^2^Sabanci University Nanotechnology Research and Application Centre, Sabanci UniversityIstanbul, Turkey

**Keywords:** miRNA, miRNA annotation, TE-miR, SUmirPredictor, SUmirLocator

## Abstract

microRNAs (miRNAs) are tiny ribo-regulatory molecules involved in various essential pathways for persistence of cellular life, such as development, environmental adaptation, and stress response. In recent years, miRNAs have become a major focus in molecular biology because of their functional and diagnostic importance. This interest in miRNA research has resulted in the development of many specific software and pipelines for the identification of miRNAs and their specific targets, which is the key for the elucidation of miRNA-modulated gene expression. While the well-recognized importance of miRNAs in clinical research pushed the emergence of many useful computational identification approaches in animals, available software and pipelines are fewer for plants. Additionally, existing approaches suffers from mis-identification and annotation of plant miRNAs since the miRNA mining process for plants is highly prone to false-positives, particularly in cereals which have a highly repetitive genome. Our group developed a homology-based *in silico* miRNA identification approach for plants, which utilizes two Perl scripts “SUmirFind” and “SUmirFold” and since then, this method helped identify many miRNAs particularly from crop species such as *Triticum* or *Aegliops*. Herein, we describe a comprehensive updated guideline by the implementation of two new scripts, “SUmirPredictor” and “SUmirLocator,” and refinements to our previous method in order to identify genuine miRNAs with increased sensitivity in consideration of miRNA identification problems in plants. Recent updates enable our method to provide more reliable and precise results in an automated fashion in addition to solutions for elimination of most false-positive predictions, miRNA naming and miRNA mis-annotation. It also provides a comprehensive view to genome/transcriptome-wide location of miRNA precursors as well as their association with transposable elements. The “SUmirPredictor” and “SUmirLocator” scripts are freely available together with a reference high-confidence plant miRNA list.

## Introduction

microRNAs (miRNAs) are small non-coding molecules which regulate gene expression at the post-transcriptional level (Jones-Rhoades and Bartel, 2004; Budak and Akpinar, 2015; Budak et al., 2015b; Alptekin and Budak, 2016; Alptekin et al., 2016). By their regulatory role in a wide range of biological activities including growth, development and stress responses, they stand as irrevocable keystones of cellular life (Fujii et al., 2005; Liu et al., 2008; Kantar et al., 2010; Alptekin et al., 2016). Since the first documentation of miRNAs from *Caenorhabditis elegans*, many different methods have been developed for miRNA identification and elucidation of their functional roles both in animals and plants (Alptekin et al., 2016). In earlier studies, many miRNAs and their target genes have been identified by several experimental approaches including cloning (Sunkar et al., 2005) splinted-ligation mediation (Chamnongpol et al., 2010) and genetic screening (Aukerman, 2003). Despite the strength of such experimental methods in the detection of genuine miRNAs, these methods are considerably time-consuming, labor-intensive and costly; thus, they are not suitable for high-throughput and comprehensive studies (Wang et al., 2005; Kleftogiannis et al., 2013). Recent technological improvements paved the way for next-generation sequencing-based approaches, such as small RNA (small RNA-Seq) sequencing which can be used for high-throughput miRNA identification (Howell et al., 2007). Additionally, advances in technology have led to substantial reductions in sequencing costs and many whole genome sequences are currently available for the discovery of miRNA genes (Egan et al., 2012; Goodwin et al., 2016). Extensive utilization of high-throughput data generated by the next-generation sequencing (NGS) platforms, in turn, promoted the advances in computational approaches for miRNA research (Sunkar et al., 2008). Recently, computational methods applied on NGS data stands as the most powerful strategy for large-scale detection of genuine and novel miRNAs together with their sequential isoforms, isomiRs (Bartel, 2004; Wang et al., 2005; Budak et al., 2014; Budak and Kantar, 2015).

There are several tools for *in silico* miRNA identification such as miRanalyzer, miR-PREFeR, miRTRAP, miRLocator, and MIReNA (Hendrix et al., 2010; Mathelier and Carbone, 2010; Hackenberg et al., 2011; Lei and Sun, 2014; Cui et al., 2015). Majority of these methods rely on the sequence information of previously validated miRNA and non-miRNA sequences such as genes (Friedländer et al., 2008; An et al., 2013), while others perform *de novo* prediction (Yousef et al., 2006; Liu et al., 2015). Considering the utilization of such information, it is possible to classify computational miRNA identification methods under two broad groups as comparative and non-comparative where they both stand with their own advantages and limitations (Kleftogiannis et al., 2013). Comparative methods are based on the conservative nature of the miRNA sequences at inter/intra species level and these methods search for the exact or near-exact matches to previously known miRNAs in a given sequencing data. Despite the high-throughput and relative ease of these methods in the detection of evolutionarily conserved miRNAs across different species, they are inadequate for discovery of novel miRNAs which do not share sequence homology with known miRNAs (Mendes et al., 2009; Kleftogiannis et al., 2013). This limitation of comparative methods gave rise to development of non-comparative methods which are based on machine learning (ML) algorithms (Yousef et al., 2009; Kleftogiannis et al., 2013). ML approaches classify miRNA stem-loops with respect to their structural and thermo-dynamical properties along with their sequential variation. These algorithms utilize some specific rules for miRNA detection, generated while training the program of the program by different datasets such as high-confidence miRNA and gene sequence sets (Williams et al., 2012; Saçar and Allmer, 2014). ML approaches have revealed the presence of many non-homologous miRNAs and were utilized for the detection of disease associated miRNAs in humans (Chen and Yan, 2014; Chen et al., 2015, 2016). However, the accuracy of ML based predictions are strongly affected by the positive and negative datasets utilized in the training process (Mendes et al., 2009); consequently, experimental methods such as northern blotting or reverse transcription PCR are generally required are generally required for validation of genuine miRNAs (Budak and Akpinar, 2015). Comparative methodologies, providing homology evidence, also benefit from experimental validation.

Many *in silico* miRNA identification methods have primarily been developed in and optimized for animals, in particular humans, since miRNAs are medically important considering the discovery of future diagnosis and treatment approaches (Esteller, 2011). Structural and functional properties of miRNAs between animals and plants are significantly different; thus, utilization of the same parameters for miRNA identification and target annotation is not an accurate approach (Mendes et al., 2009; Axtell et al., 2011). In plants, the level of conservation of miRNA precursors (pre-miRNAs) is relatively low, in contrast to animals where pre-miRNAs and their thermodynamic stabilities are more conserved. In addition, plant miRNA stem-loops vary remarkably in length (Ni et al., 2010). Consequently, the identification of plant miRNAs put more emphasis on the detection of appropriate miRNA:miRNA^*^ duplexes on the miRNA precursor (Mendes et al., 2009). The differences in genome structure and organization, even within the plant, also affect miRNA identification process. Many of the economically important plant species, particularly cereals, have a high proportion of repetitive sequences in their genomes (Brenchley et al., 2012; Mehrotra and Goyal, 2014) which might cause several problems in miRNA identification. Also, polyploidy observed in certain plant genomes, such as wheat and barley further exacerbate the accuracy of miRNA quantification and discrimination of homologous copies of miRNAs both in *in silico* and experimental analyses (Mendes et al., 2009; Kleftogiannis et al., 2013). Considering all the above-mentioned issues, specialized criteria for miRNA identification and annotation are required for both animals and plants, and the utilization of separate tools optimized for each group is highly suggested (Meyers et al., 2008). Computational approaches, in particular, benefitting from the homology-based support, have achieved large-scale and efficient detection of plant miRNAs (Kantar et al., 2010; Kurtoglu et al., 2014; Wu et al., 2014; Akpinar et al., 2015; Ebrahimi Khaksefidi et al., 2015; Akpinar and Budak, 2016). In such studies, the selection of reference miRNA set, used in these homology-based approaches, has a great impact on the accuracy of miRNA identification. There are several miRNA databases available for selection of the reference miRNAs and miRBase is the most comprehensive and updated one among these (Zhang et al., 2010; Kozomara and Griffiths-Jones, 2011; Szcześniak and Makałowska, 2014; Budak et al., 2015a). In the current release of miRBase (Release 21), there are more than 2000 miRNA families belonging to 72 different plant species. Only 176 of these miRNAs, however, belonging to 17 species are annotated as “high-confidence” (Kozomara and Griffiths-Jones, 2014). A Majority of the plant miRNAs in miRBase have been identified by homology-based *in silico* methods and mostly lack experimental evidence. Utilization of computationally identified miRNAs, lacking experimental validation or other types of further support, in *in silico* miRNA identification may lead to an overpopulation of false-positives in the process of miRNA identification. Thus, utilization of experimentally supported miRNAs may provide more reliable *in silico* miRNA identification results. Additionally, redundancies in miRNA naming and mis-annotations in mature miRNAs in the current release of miRBase can give rise to further redundancies and contradictions in the downstream miRNA analysis processes such as determination of genomic distribution and quantification of identified putative miRNAs (Van Peer et al., 2014; Budak et al., 2015a). Hence, the annotation of genuine miRNAs from the pool of candidates requires closer inspection. A homology-based *in silico* methodology for plant miRNA identification was developed in 2012 (“SUmirFind” and “SUmirFold,” Lucas and Budak 2012). Herein, we report further refinements and improvements to this methodology, enabling increased sensitivity and sensibility, in response to the complications associated with the aforementioned plant genome attributes. Also, we present a high-confidence miRNA list, selected from the entries deposited on miRBase (release 21) that should aid in computational identification of plant miRNAs with reduced number of false-positive predictions (Kozomara and Griffiths-Jones, 2014). The current pipeline was tested on both genomic and transcriptomic sample data from *Brachypodium distachyon* and *Triticum aestivum*, revealing high confidence miRNAs belonging to more than 20 miRNA families. Our results provided valuable insight to the miRNAome of these two plants together with their specific targets.

## Materials and methods

### Workflow

#### Input data set and software dependencies

**Figure 1:**
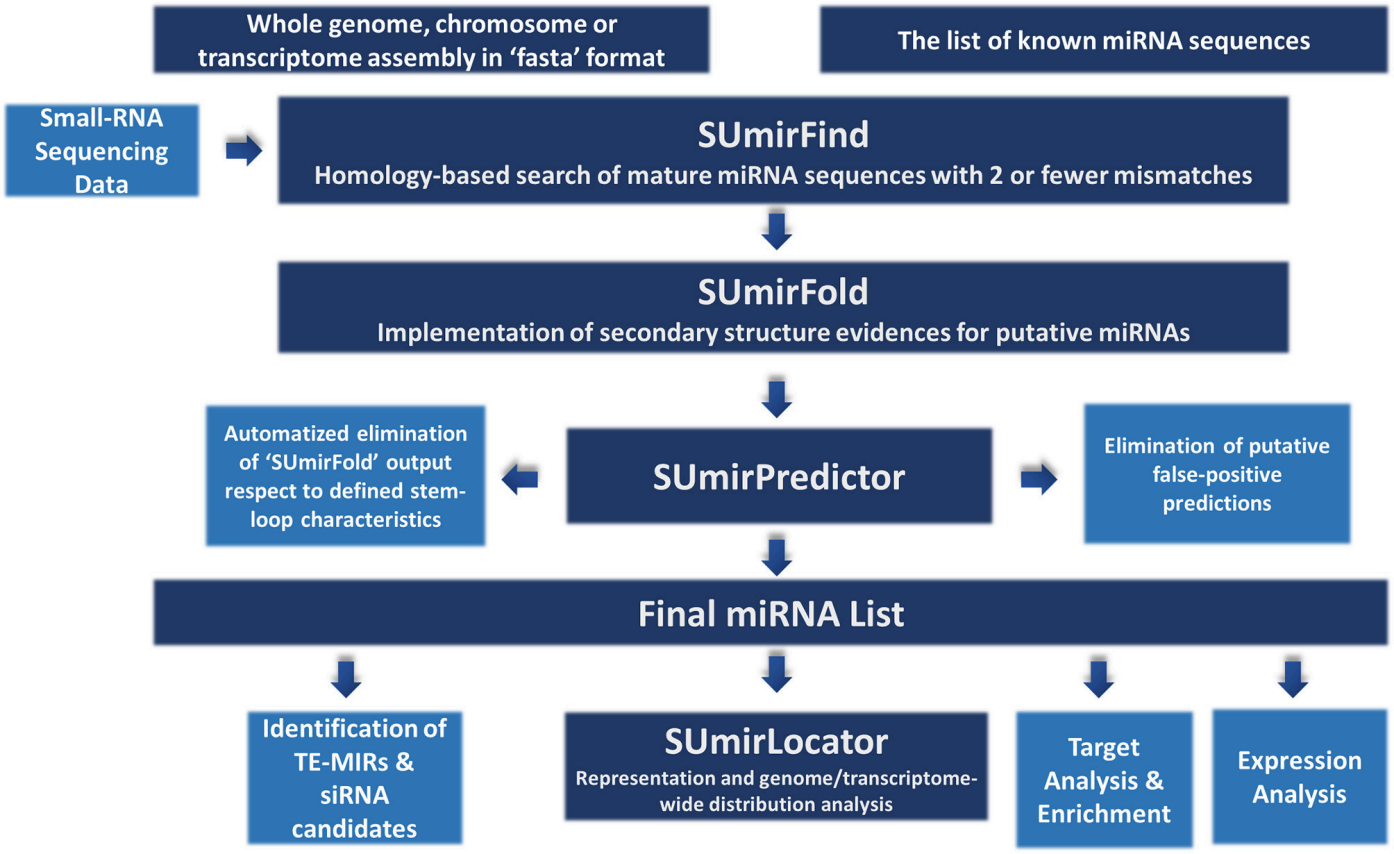
**An overview about miRNA identification methodology**. The pipeline accepts sequences from genomic and transcriptomic data in “fasta” format. It can also work with small RNA sequencing data with some modifications. “SUmirFind” script searches for detection of any putative miRNAs by alignment of sequences to known plant miRNAs with 2 or fever mismatches. Candidate sequences are then searched for presence of pre-miRNA-like secondary structures by “SUmirFold” while the candidates are further eliminated by “SUmirPredictor” based on miRNA precursor characteristics. Potential miRNA sequences are also inspected for detection of any false-positive predictions with the alignment of candidates to other known small RNA sequences and organellar genomes. The obtained final list of mature miRNAs and their precursors are inspected with a few more analysis for characterization and annotation of miRNAs. Detected putative pre-miRNA structures are further evaluated for the representation and genomic/transcriptomic distribution analysis with the help of “SUmirLocator” script. Target identification and enrichment analysis of miRNA candidates are conducted based on “psRNAtarget” and Blast2GO software. Candidate miRNAs are also analyzed for the *in silico* expression evidence at both pre-miRNA and mature miRNA level. Additionally, miRNA precursors are searched for understanding their association with transposable elements (TE) and based on their relation level; they are further characterized as TE-miRs or siRNA candidates.

The methodology for miRNA identification from genomic/transcriptomic and small RNA-Seq data is summarized in Figure 1. Our miRNA identification pipeline was originally designed for utilization of high-throughput genomic and transcriptomic sequences in FASTA format as input and any list of reference mature miRNAs as query for homology-based exploration of putative miRNA sequences. The pipeline can also be used for small RNA-Seq data with additional modifications (See section “Adaptation of pipeline for small RNA-Sequencing data”). For relatively short raw input DNA/RNA sequences from genomic/transcriptomic data, sequences must be assembled into contigs prior to analysis since the sequences which are longer than 200 nucleotides (nt.) are more suitable for an accurate analysis or miRNA precursors. Additionally, the chosen reference miRNA set is crucial for accurate mining of miRNAs and the use of a list of “high-confidence” or “experimentally-validated” miRNAs is strongly encouraged.

Pre-installation of a few software is required in order to run our miRNA identification pipeline. The Blast++ standalone tool kit (Camacho et al., 2009) and UNAFold software (Markham and Zuker, 2008) together with a Perl programming environment is required for the minimal use of the pipeline. For the complete pipeline Blast2GO (Conesa and Götz, 2008), RepeatMasker (Tarailo-Graovac and Chen, 2009), and GMAP (Wu and Watanabe, 2005) are recommended for functional annotation, repeat masking and sequence alignments. These optional programs can be replaced by similar software depending on user's choices; however, in this case, optimization of alternative programs may be required. Additionally, NGS assembly software may be required if the input data is composed of relatively short reads. The choice of the NGS assembly software will depend on the sequencing platform from which the NGS data was obtained and to the user's preferences.

#### Homology-based miRNA identification with “SUmirFind,” “SUmirFold,” and “SUmirPredictor”

This miRNA identification pipeline basically utilizes two sequential and easy-to-use Perl scripts, “SUmirFind” and “SUmirFold” which were originally published in 2012 (Lucas and Budak, 2012) and successfully employed in the identification of several miRNAs, particularly from cereal species (Kurtoglu et al., 2013, 2014; Akpinar et al., 2015; Alptekin and Budak, 2016). Here, a new Perl script, “SUmirPredictor” (Supplementary Document 1), is added to our pipeline which automates the final evaluation of candidate sequences and generation of final miRNA list. This script also provides a unique name for each putative miRNA based its location on the hairpin-shaped miRNA precursor and its homologous reference miRNA. The workflows of these three scripts are detailed below.

At the first step of our miRNA identification pipeline, “SUmirFind” script searches for potential miRNA candidates within given input sequences, by aligning the reference miRNA list, comprised of known mature miRNA sequences, with two or less mismatches, using the BLAST algorithm (Camacho et al., 2009). Following the identification of miRNA candidates, “SUmirFold” script submits a ~700 nucleotide long fragment flanking the putative mature miRNA sequence to UNAFold to generate and evaluate the potential secondary structures of miRNA precursors (Markham and Zuker, 2008). “SUmirFold” picks the secondary structure with the lowest Minimum Free Folding Energy (MFE) and discards potential miRNA candidates if the respective fold-back structure of miRNA precursor, also called “hairpin,” fails to fulfill criteria for being genuine miRNA precursor (explained in Lucas et al., 2012). Additionally, “SUmirFold” marks the candidate sequences as “suspects” and list them as a separate output when putative miRNA-miRNA^*^ duplexes do not contain any mismatches, since such sequences may correspond to inverted repeats or siRNA sequences (Lucas and Budak, 2012). If a fold-back structure carrying a potential mature miRNA satisfies all the criteria, “SUmirFold” excises the sequence from 20 nucleotides away of the mature miRNA start site and refolds in order to form the hairpin-shaped precursor of miRNA, also referred as pre-miRNA. The results of “SUmirFold” process are written into text files contain the information about putative mature miRNAs and their precursors along with post-script format by UNAFold enabling the visualization of hairpin structures of precursors.

“SUmirFind” and “SUmirFold” scripts basically provide evidence for the presence of an appropriate secondary structure, a “putative miRNA precursor or pre-miRNA,” which contains a candidate mature miRNA sequence within. Finally, “SUmirPredictor” evaluates qualified potential precursor sequences with respect to the following pre-defined pre-miRNA characteristics based on previous observations on genuine miRNA features (Meyers et al., 2008; Kurihara and Watanabe, 2010).

1. Potential precursors, or hairpins, cannot have multi-loop structures above the mature miRNA location.
2. Mature miRNA and miRNA^*^ sequences cannot extend into the head section of the hairpin.
3. Mismatches at the DICER-LIKE enzyme cut regions of mature miRNA and miRNA^*^ sequences are not allowed.

**Figure 2:**
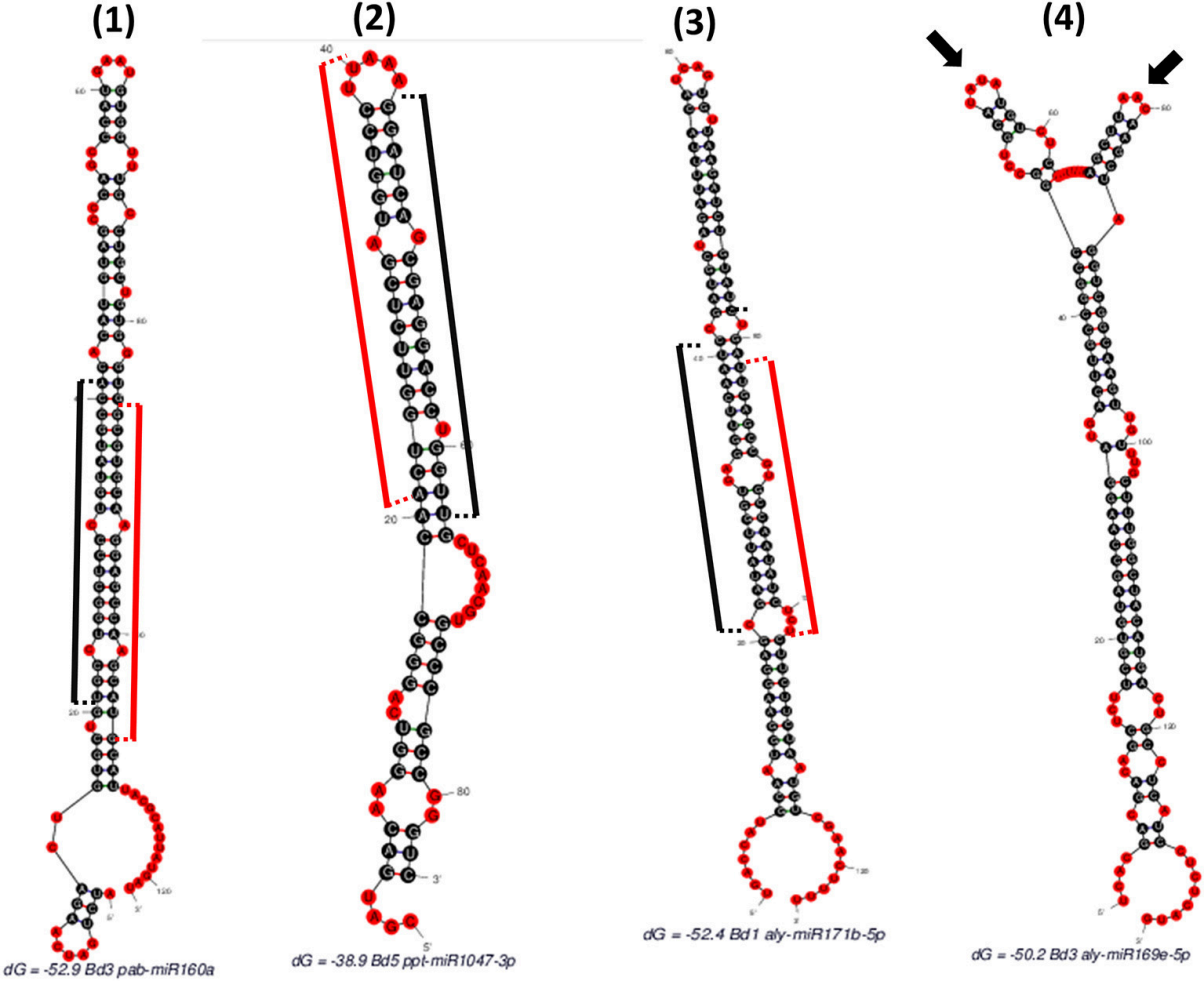
**Different hairpins obtained as “SUmirFold” outputs and their filtration process with “SUmirPredictor”**. **(1)** Mature miRNA starts at 21th base and ends at 41th base where the miRNA^*^ starts at 86th base and ends at 106th base [indicated by black (mature miRNA) and red (miRNA^*^) sticks]. There is no mismatch in the DICER-LIKE enzyme cutting region and there is a proper loop structure. Such structures are remarked as “OK” by “SUmirPredictor” since it represents genuine miRNA characteristics. **(2)** Mature miRNA start site aligns between 44 and 64th bases where the miRNA^*^ detected in between 21 and 40th bases. Since the two nucleotides of miRNA^*^ aligns in the head section of hairpin structure, DICER-LIKE enzyme may not process it properly; thus, this miRNA is remarked as “Head” by “SUmirPredictor”. **(3)** Mature miRNA aligns between 21 and 41th bases and there is a mismatch on the start area of mature miRNA where the DICER-LIKE enzyme cutting region located. Enzyme may not able to process this sequences and such structures are remarked as “Dicer-cut”. **(4)** miRNA precursor has more than one loop structure on its head, so this structure is remarked as “Multiloop” by “SUmirPredictor” (Two different loops were indicated by arrows).

**Figure 3:**
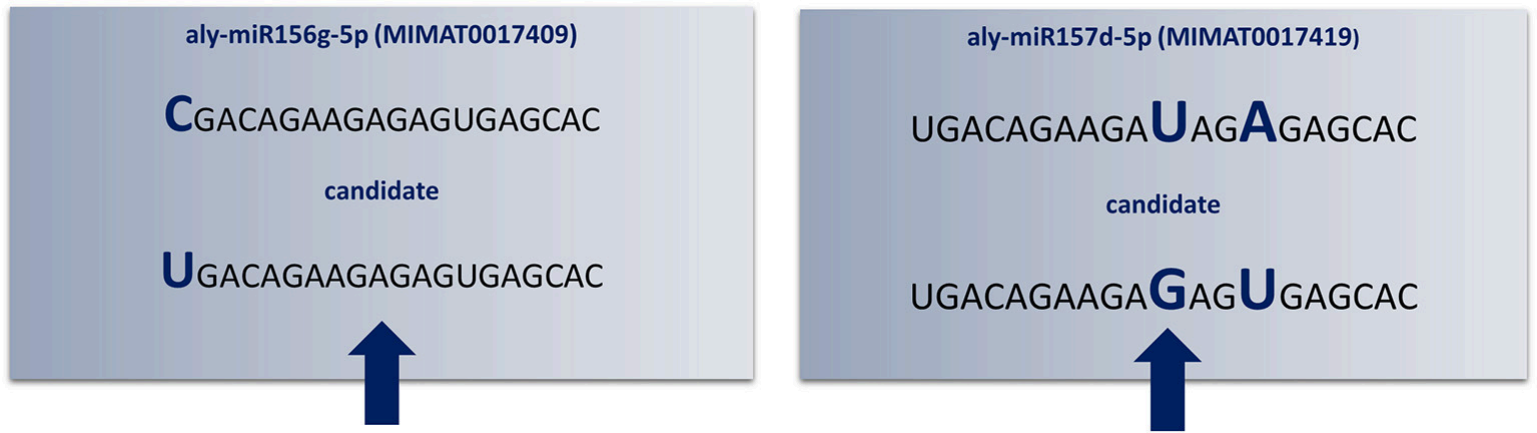
**Redundant annotations detected by “SUmirPredictor”**. Up to three mismatches criteria used in the initial identification of candidate mature miRNA sequences may lead to redundant annotations of the same candidate sequence (indicated by arrows). Here, *Arabidopsis lyrata* miR156g-5p and miR157d-5p had mismatched bases in different locations; consequently, the same mature miRNA sequence appears as twice as two different candidates (Aly: *Arabidopsis lyrata*). Since the aly-miR156g-5p displayed higher sequence homology to putative mature miRNA sequence, candidate miRNA named as miR156 and miR157 was eliminated.

“SUmirPredictor” directly processes the output of “SUmirFold”. It must be noted that the “suspect” miRNA candidates which are marked and separated by “SUmirFold” should be independently processed by “SUmirPredictor” since the pool of these candidates have a higher potential for false-positives such as confusion with siRNAs (Lucas and Budak, 2012). “SUmirPredictor” outputs two separate files: (1) Output file of the “SUmirFold” scripts with remarks on each potential precursor: “OK” for qualifiers; “Multiloop,” “Head,” “Dicer-cut” for non-qualifiers, indicating the criterion failed to be fulfilled (Figure 2), (2) Qualifiers list, including candidate miRNA name—mature miRNA/miRNA^*^ and pre-miRNA sequences—homolog reference miRNA name. It should be noted that the pre-miRNA structures referred as “Multiloop,” which have branched loops at their terminal end, are particularly discarded from the pool of genuine miRNAs by “SUmirPredictor” since the stability of such structures is problematic at the pre-miRNA level despite appearing as genuine candidates at the pri-miRNA level (Zhu et al., 2013). The second output file of “SUmirPredictor” filters redundant blast hits blast hits which may result in mis-annotation of putative miRNA sequences. Since up to two mismatches are allowed in the identification of candidates homologous to known plant miRNAs by “SUmirFind,” single mature miRNA may be annotated as more than one miRNA family and each annotation may align to the same candidate sequence with different sets of mismatches (Figure 3). “SUmirPredictor” eliminates such redundancies by picking the best annotation with highest similarity to know plant miRNA sequence and outputs the following information in the secondary output file: “miRNA ID—mature miRNA sequence—pre-miRNA sequence–index number (index of the respective entry in the first output file of “SUmirPredictor” for tracing back option if needed)-conserved miRNA ID-miRNA^*^ sequence' information. In case of equal similarity to different conserved miRNAs, the all conserved miRNAs IDs are separated by a comma and selection of the most suitable name for candidate miRNA sequence is left to user preference. In this step, blast searches (Camacho et al., 2009) for candidate miRNA precursors might be performed to elucidate the similarity between the precursor of redundantly-annotated homolog miRNAs and putative miRNA name might be provided based on the highest similarity score; however, such results may represent controversies regarding the non-conserved nature of plant pre-miRNAs.

Discrepancies in the naming of newly identified miRNAs are problematic for miRNA researchers and no solutions have been presented to this problem yet (Budak et al., 2015a). Problems with miRNA naming include miRNAs from the same miRNA family, having widely differing sequences, due to the location of the mature miRNA on the precursor (either 3′ or 5′ end of precursor sequence). For such miRNAs, we propose a revised naming to avoid confusion:

1. If the homolog miRNA from the reference list has the hairpin arm information such as **“miR156a-3p”** and if the newly identified miRNA is also generated from the same arm of the hairpin-shaped precursor (in this case, 3′ of precursor sequence), the newly identified also carries the hairpin arm information (in this case, **miR156-3p**).
2. If the homolog miRNA from the reference list does not have the hairpin arm information, such as **“miR156a,”** the newly identified miRNA is named according to the location of the mature miRNA detected by “SUmiRFold” (If the miRNA is detected on the 3′ arm of the hairpin, the miRNA is named as **miR156-3p**).
3. If the homolog miRNA from the reference list has the hairpin information such as **“miR156a-3p”** and if the newly identified miRNA is generated from the opposite arm of the hairpin-shaped precursor (in this case, 5′of precursor sequence), the newly identified miRNA takes just the family ID of conserved miRNA sequence, without the presence of any extensions (in this case, **miR156**).

“SUmirPredictor” adjusts the name of each newly identified miRNA according to above-defined rules. It does not specify the letter extensions of miRNA IDs which requires a more comprehensive analysis of pre-miRNA structures at the miRNA family level (Budak et al., 2015a).

“SUmirPredictor” also discards all potential miRNA^*^ sequences for defined mature miRNAs. One mature miRNA sequence might be associated with several miRNA^*^ sequences which may vary in their flanking regions. Also, in some cases, these differences may arise from the small bulges reside inside the mature miRNA/miRNA^*^ duplex. In this pipeline, all potential miRNA^*^ for each mature miRNA are reserved and further utilized in the process of small RNA expression analysis (See section: “miRNA expression analysis as an *in silico* evidence for the genuineness of putative miRNAs”), however, one can eliminate and determine particular miRNA^*^ sequence for a defined mature miRNA. Finally, “SUmirPredictor” eliminates the miRNAs which have any undefined sequences marked with “N” in their mature miRNA sequences since mature miRNAs are short (around 20 nt.) and existing undetermined sequences may lead to false-positive results in course of target identification.

#### Elimination of putative false-positive predictions from new miRNA pool

In some cases, other small non-coding RNA species such as transfer RNA (tRNA), ribosomal RNA(rRNA), small nuclear RNA (sn-RNA), and small nucleolar RNA (sno-RNA) together with repetitive elements may generate false-positive predictions in miRNA identification process (Kang and Friedländer, 2015). However, elimination of such sequences is controversial for miRNA mining considering recent studies revealing the presence of miRNAs within tRNA genes (Maute et al., 2013). Thus, decision for sorting out of these sequences depends on the nature of the dataset and focus of research. In this updated pipeline, any non-coding RNA species and repetitive sequences are not eliminated prior to the miRNA mining analysis. Following the miRNA identification, both mature miRNA and their precursor sequences are aligned to other non-coding RNA species using BLAST and positive hits which have query identity and coverage with more than 95% are eliminated. Additionally, pre-miRNA sequences are further analyzed in order to detect transposable element related miRNAs [See Section: Identification of Transposable Element Related miRNAs (TE-MIRs)]. Manycrop species have contain high quantities of transposable elements in their genomes which may code for thousands of functional miRNAs (Piriyapongsa and Jordan, 2008; Li et al., 2011).

Although the lack of evidence for the presence of miRNAs coming from organellar genome in plants, a few organelle-associated miRNAs have been detected in humans (Sripada et al., 2012). Considering the potential presence of organelle associated miRNA sequences across the candidate miRNA pool, mature and pre-miRNA sequences are aligned to organellar genomes with BLAST and putative miRNA sequences matching organellar genomes, namely mitochondria and chloroplast, are separately recorded.

#### Exploration of miRNA distribution and miRNA representation by “SUmirLocator”

**Figure 4:**
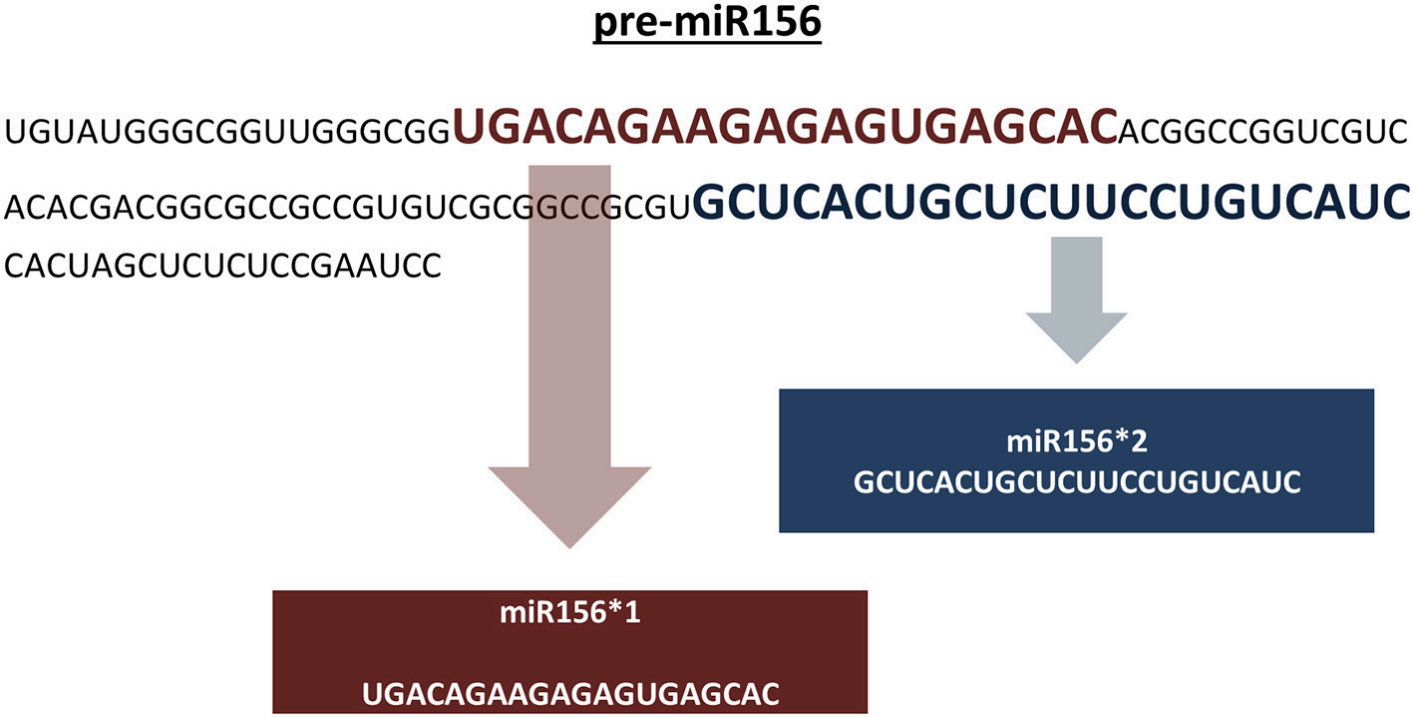
**Including pre-miRNA sequences which codes for different mature miRNAs into miRNA representation**. Putative pre-miR156 sequence is predicted to encode two distinct mature miRNA sequences for miR156 family. Both of these miRNAs are included in the genomic representation as separate units. It must be noted that these sequences are not mature miRNA/miRNA^*^ pairs; instead, they are two different sequences belonging to miR156 family.

Genomic representation refers to the sum of all genomic locations of a miRNA family in a genome across its chromosomes. Genomic representation analyses provide insights into the distribution and organization of miRNA genes across the genome, providing information about putative miRNA genes grouped in specific chromosomal locations. In the absence of a reference genome sequence, genomic assemblies composed of non-overlapping sequence contigs can still provide important clues into the genomic distribution of miRNA families. With respect to our representation definition, pre-miRNAs identical in sequence are included in the overall representation if: (1) the pre-miRNA sequence is predicted from different positions on the same assembled sequence, and (2) the pre-miRNA sequence is predicted from a single genomic location that carries two different mature miRNA sequences (Figure 4). In our pipeline, putative miRNA precursors together with the mature miRNA sequences which satisfy afore-mentioned criteria are further analyzed for their genomic representation using an additional Perl script, “SUmirLocator” (Supplementary Document 1). Using the input of genomic data which is utilized in *in silico* miRNA identification in prior steps, script defines the location of unique pre-miRNAs individually. Also, it counts the occurrence of unique locations for each member of the same miRNA family in order to identify genome-wide copy number variation among miRNA families, in other words, their respective representations. Running time for “SUmirLocator” analysis is relative to the size of input data, genome size and the number of predicted pre-miRNA sequences. The output is contained in two comma-delimited files, “pre-miRNA-count” and “pre-miRNA-location,” and one text file “expression.tbl,” respectively. The first file contains detailed information about the locations of the miRNA precursors including the strand information as “sense” or “antisense.” The second file provides a summary for the family-based count of miRNAs and basically presents the miRNA representation. The text output is generated for downstream *in silico* miRNA expression analysis. Additionally, “SUmirPredictor” reserves all associated the miRNA^*^ sequences for each mature miRNA and gives the user preference of either choosing one of the sequences or analyzing all of them. If miRNA^*^ sequences are selected, “SUmirLocator” rechecks the miRNA^*^ sequences and writes them as a separate output file which will be used for down-stream expression analysis (See section: miRNA expression analysis as an *in silico* evidence for the genuineness of putative miRNAs).

“SUmirLocator” can also be used for identified miRNAs from transcriptomic data where the transcriptome-wide representations (in this case the total count of miRNA families with members coming from different contigs including different isoforms) of miRNA families are assessed. If transcriptomic data is utilized, “SUmirLocator” outputs for pre-miRNA sequences may provide a rough idea about alternative splicing of miRNA genes. A closer inspection of miRNA sequences generated from different transcriptomic isoforms of the same gene may offer information about alternative splicing patterns of related miRNA genes together with its effect on mature miRNA sequences. Alternative splicing of miRNA genes during transcriptional process might have influence on the pre-miRNA sequences by resulting in different stem-loop like structures (Melamed et al., 2013; Agranat-Tamir et al., 2014). On the other hand, different pre-miRNA like stem-loop structures obtained from various isoforms of the same miRNA gene may make inroads to the formation of distinct mature miRNA sequences which may regulate separate targets (Melamed et al., 2013). In our analysis, miRNA family members coming from all contigs including different transcriptomic isoforms are also counted and recorded as representation of related miRNA family. Additionally, in case the presence of high quality genome data, the pre-miRNA sequences obtained by miRNA mining from transcriptomic data, can be aligned back to the genome with the help of alternative splicing-aware aligners such as GMAP (Wu and Watanabe, 2005) and potential alternative splicing of miRNA genes are confirmed by comparison with “SUmirLocator” results.

The representation of miRNA families may also give a rough idea about the relative expression levels of particular miRNAs, however; the representation of *in silico* identified miRNA families may not necessarily agree with experimental results. Since the miRNA expression is highly time/condition/tissue specific, the experimental condition where the data generated for *in silico* miRNA mining and experimental validation must be same or similar (Budak et al., 2014). In all cases, validation of *in silico* identified miRNAs with experimental techniques such as, qPCR or Northern Blotting, should provide a more accurate profile for differential expression of miRNAs of interest.

#### miRNA expression analysis as an in silico evidence for the genuineness of putative miRNAs

In miRNA biogenesis, miRNA genes are first transcribed into pri-miRNA sequences by the activity of RNA Polymerase II, which are then poly-adenylated and capped similar to mRNA sequences (Kurtoglu et al., 2013). Consequently, miRNA precursors can be detected in the ESTs and cDNA libraries along with transcriptome assemblies. In order to provide evidence for actual expression of putative pre-miRNA sequences, identified pre-miRNAs are aligned to the EST/cDNA sequences and assembled transcriptomic data via BLAST Algorithm (Camacho et al., 2009). The sequences which satisfy a specific cut-off for query identity and query coverage, determined by users with respect to expression dataset and query, are defined as “*in silico* expressed miRNA precursors.” In addition to this expression evidence analysis, the reads from small RNA-Seq studies can be aligned back to the putatively identified pre-miRNAs to ensure the genuineness of computationally identified miRNA precursors. Pre-miRNA sequences which have small RNA reads aligning with the *in silico* predicted miRNA/miRNA^*^ regions increases the confidence that these sequences do represent precursor sequences for functional and expressed miRNAs.

This pipeline also provides an *in silico* expression evidence for putative mature miRNA/miRNA^*^ duplexes, utilizing BLAST alignments of putative miRNA/miRNA^*^ sequences to small RNA sequencing data. A series of specific parameters are set for optimized alignment of short miRNA sequences to small RNA-Seq reads: -task *blastn-short*–*ungapped*–dust “*no”*–e-value *1000*–wordsize *7*–strand “*plus.”* Importantly, the short blast mode option is used (-task *blastn-short*) with a combination of ungapped parameters in order to provide a reliable alignment of mature miRNA and miRNA^*^ sequences to small RNA-Seq reads. Also, a high e-value is employed in alignment process since the expectation value for short sequences is higher compared to longer ones. Both miRNA and miRNA^*^ sequences which are also obtained as the output of “SUmirLocator” process, inside the “expression.tbl” file, are aligned to small RNA reads, trimmed and cleaned from adaptor sequences. miRNA/miRNA^*^ sequences which are present in the small RNA-Seq libraries with 100% query identity and coverage are accepted as “expressed.” The 100% cut-off is specifically chosen for mature miRNAs since these sequences are tiny and any mismatch tolerant alignment can affect the sensitivity of the analysis. If at least three reads from the small RNA sequencing data match each of the mature miRNA and miRNA^*^ sequences with the criteria above, then the predicted miRNA is accepted as “*in silico* expressed.” This step is highly recommended to increase the reliability of the computationally identified miRNAs, since the presence of mature miRNA/miRNA^*^ duplex is essential for the validation of mature miRNA expression (Kozomara and Griffiths-Jones, 2014). The cutoff for the number of small RNA reads aligned to each mature miRNA and miRNA^*^ sequences can be modified by the user preferences; however, this pipeline suggests at least three matching reads considering the scarcity of plant small RNA sequencing experiments, compared to animals, where the already available data volume allows the ccutoff of 10 or more reads for *in silico* evidence of miRNA sequences (Kozomara and Griffiths-Jones, 2014).

#### Identification of transposable element related miRNAs (TE-miRs) and potential small interfering RNA (siRNA) candidates

Certain plant miRNAs are known to be identical or homologous to transposable elements (TE), which are generally termed as “Transposable Element-related miRNAs” or “TE-miR” (Li et al., 2011; Kantar et al., 2012; Kurtoglu et al., 2014). This miRNA identification pipeline checks for the presence of such miRNAs by comparing the putative miRNA precursor sequences against a given database of repeat elements using RepeatMasker software that employs the Cross-Match alignment algorithm (http://www.phrap.org/phredphrapconsed.html) (http://www.repeatmasker.org/). The precursor sequences covered by repeats more than 50% of their lengths are recorded as TE-miR. The potential TE-miRs are also further analyzed for repetitive element distributions by repeat families.

Small interfering RNAs (siRNAs), a class of double-stranded RNAs of 20–25 nucleotides in length, exhibit many similarities to miRNAs despite the presence of major differences in their biogenesis (Tang, 2005). siRNAs are generated from perfectly base-paired, long, double stranded RNAs, by the activity of several members of DICER-LIKE enzymes (Parent et al., 2015). siRNAs generally target the same gene from which they are transcribed although there are some examples of non-self-targeting siRNAs such as trans-acting siRNA (ta-siRNA) (Zhang et al., 2014). Thus, transposable elements are popular targets of siRNA molecules and in fact, siRNAs are thought to be evolved for transposon silencing in order to protect the genome and sustain the genomic stability (Ito, 2012). Conversely, miRNAs have the ability to target many other genes different from their precursor (Carthew and Sontheimer, 2009). There are several hypotheses about the shared origin of TE-miRs and siRNAs where they both are very similar or identical to TEs (Piriyapongsa and Jordan, 2008) and because of this similarity, it is troublesome to differentiate between TE-miRs and siRNAs. Our pipeline first reports potential siRNA molecules at the “SUmirFold” step where the perfectly complementary miRNA/miRNA^*^ duplexes are separated from the main output as “suspects” (Lucas and Budak, 2012). In addition, miRNA precursors passing all criteria set by “SUmirFind,” “SUmirFold,” and “SUmirPredict,” are further analyzed for repetitive content as described above. Precursor sequences with almost perfect complementarity to TEs (up to 3 mismatches allowed) following the TE-miR analysis are accepted as “potential TE-miR” which may also include some siRNA sequences.

#### miRNA target analysis and target enrichment

miRNAs mainly target mRNAs and regulate their expression by inhibiting the translation of them into functional proteins through translation repression or by suppressing their transcription via mRNA cleavage (Zhang et al., 2006; Rogers and Chen, 2013). Identification of genuine plant miRNA targets is crucial for understanding their effects of at the molecular level. This pipeline employs an online tool for detecting putative miRNA targets, “psRNATarget” (http://plantgrn.noble.org/psRNATarget/), selected as one of the most reliable and precise tool for miRNA target mining (Dai and Zhao, 2011; Srivastava et al., 2014). psRNATarget takes both target complementarity and target site accessibility into consideration, together with the assessment of multiple target sites present in a given mRNA molecule; thus, it may assign more than one target for a given miRNA sequence (Dai and Zhao, 2011). The target sequences identified by psRNATarget are then annotated through similarity searches against annotated protein databases from all or related plants using BlastX tool of the BLAST toolkit (Camacho et al., 2009) and Gene Ontology (GO) annotations are retrieved using Blast2GO software at Biological Process (BP), Molecular Function (MF), and Cellular Component (CC) levels (Conesa and Götz, 2008).

Following miRNA target identification and annotation, the most significant target with a known functional annotation for each mature miRNA is identified with the following procedure based on the two important parameters defined by psRNATarget tool for putative miRNA-miRNA targets pairs: UPE and Expectation. “UPE” is the binding energy between miRNA and its target pair, and lower UPE values indicate a better miRNA-target binding. “Expectation” is a statistical measure, based on the randomness of particular miRNA sequences to bind a mRNA molecule. Low “expectation” values, similar to e-value in blast, demonstrates statistically more significant miRNA-target pairs (Dai and Zhao, 2011). For detection of the most significant targets, UPE and expectation values are summed up for a given target for all targets of the same miRNA and the target sequence which has the lowest sum is reserved as most enriched miRNA target. In case the same UPE + expectation values, the abundances of distinct Blast2GO functional annotations are taken into account and the most abundant target is picked as the most significant. It must be noted that this procedure operates only on targets with known functional annotations to enable the evaluation of a miRNA in a functional context together with its target; therefore, hypothetical or predicted targets, targets with unknown functions and with no known homologs are excluded from the target enrichment analysis.

In addition to detection of the most significant targets based on the previously explained analysis, determination of the most statistically-significant GO terms for a given miRNA family may provide insights about the pathways where the miRNA is functioning. For enrichment of statistically significant GO-terms, Blast2GO software can be utilized since it has an integrated Fisher's exact test analysis tool (Conesa and Götz, 2008). In order to detect enriched GO-terms, the Fisher's exact test can be performed separately for each miRNA family followed by retrieving associated target transcript IDs. The outputs of statistical enrichment can also eliminated further based on FDR < 0.05.

#### Modification of the pipeline for miRNA prediction from small RNA sequencing data

Although our methodology was originally designed and automated for the prediction of homologous miRNAs from relatively long next-generation sequence reads and/or assemblies, this method can also be adapted to process small RNA reads. This adapted version of the methodology incorporates an initial step of sequence similarity analysis of short small RNA reads to the homolog miRNAs with a Perl script “SUmirFind_smRNA.pl” (Supplementary Document 1). This script basically utilizes the same procedure with “SUmirFind.pl,” however; the blast code is specifically optimized for identification of small RNA reads which shows similarity to the known miRNAs. Following the determination of miRNA-like small RNA reads, sequences aligned to known miRNAs with 2 or fever mismatches, the aligned part of reads to known miRNA are trimmed with an in-house script and aligned back to the genomic or transcriptomic data to discard the candidate miRNA precursors via “SUmirFind” and “SUmirFold.” In this secondary “SUmirFind” process, the trimmed small RNA reads are utilized instead of homolog miRNA list without allowing any mismatches to determine the genomic/transcriptomic encounter sequence of sRNA reads. “SUmirFold” utilize the outputs of the second “SUmirFind” process to mark and discard the pre-miRNA sequences which is followed by “SUmirPredictor” and down-stream analysis processes. It must be noted that miRNA mining with our methodology from small RNA sequencing reads is relatively slow compared to genomic/transcriptomic miRNA mining since it is not originally designed for this process. However, it has the capability to identify both homolog miRNA and their new family member with the precise determination of their precursor structures.

### Evaluation of the new pipeline for genuine miRNA identification

In order to analyze the efficiency and accuracy of our pipeline, both genomic and transcriptomic data belonging to diploid Brachypodium and hexaploid bread wheat were evaluated following the procedure summarized in Figure 1: Whole genome assembly of *B. distachyon* cultivar Bd21 [genome version 3.0, downloaded from “Phytozome11” website (https://phytozome.jgi.doe.gov/), leaf transcriptome assembly of *B. distachyon* cultivar Bd1-1 (SRA 17815, obtained from https://trace.ddbj.nig.ac.jp/DRASearch/submission?acc=SRA171815], *T. aestivum* cultivar Chinese Spring genome (The International Wheat Genome Sequencing Consortium, 2014) and *T. aestivum* transcriptome data from spike tissues of cultivar Chinese spring (obtained from Unité de Recherche Génomique Info (URGI, http://wheat-urgi.versailles.inra.fr/Seq-Repository/RNA-Seq). The *de novo* assembly transcriptomic data from raw reads was constructed by Trinity software (Grabherr et al., 2011). Quality trimming and adaptor removal of reads were performed by Trimmomatic (v 0.32) using default parameters “LEADING:5, TRAILING:5, MINLEN:36” (Bolger et al., 2014). After assessment of the assembly quality, all three datasets from the two species were used for miRNA identification.

A reference list of miRNAs was constructed based on miRBase Release 21 for this study (Kozomara and Griffiths-Jones, 2011). Mature miRNA sequence of miRNAs referred as high confidence by miRBase or with experimental evidence were combined. Among these, redundant mature miRNA sequences were eliminated and a non-redundant list of reference miRNAs was obtained. This non-redundant list of 1404 miRNAs was utilized for all downstream analyses (Supplementary Document 2). It should be noted that only miRNAs either cloned with PCR and/or validated by Northern Blotting or Real-Time quantitative PCR were accepted as “experimentally-validated” since these are highly reliable experimental methods of miRNA detection (Chen et al., 2010).

For candidate miRNA sequences with more than one assigned miRNA family IDs by SUmirPredictor, a single miRNA ID was chosen. For the renaming process, the pre-miRNA sequences of mature miRNAs in miRBase21 were aligned to the putative, newly-identified miRNA precursors. The miRNA family ID with the highest similarity in precursor sequences was picked. The predicted miRNA sequences were further evaluated for any false-positive predictions. Both mature miRNA sequences and miRNA precursors were blasted against the sequence of other non-coding RNAs (tRNA, rRNA, sn-RNA, and sno-RNA) which were gathered from National Center of Biotechnology Information (NCBI) (http://www.ncbi.nlm.nih.gov) and European Nucleotide Archive (ENA) (http://www.ebi.ac.uk/ena) databases (-dust “*no*,” -e-value “*1e-5”*). Additionally, all obtained mature miRNA sequences together with their precursors was aligned to the organellar genomes (for *B. distachyon* chloroplast sequence, Genbank Acc. No: EU325680.1, for *T. aestivum* chloroplast sequence, Genbank Acc. No: KC912694.1, for *T. aestivum* mitochondrion sequence, NCBI Ref. No.: NC_007579.1) using parameters as -dust “*no*,” -e-value “*1e-15.”* Positive matches with >95% identity and query coverage were excluded. Subsequently, the representation of putative miRNAs was assessed by “SUmirLocator.” In the case of transcriptomic miRNA mining, pre-miRNA sequences were aligned back to the respective genome with splicing-aware alignment program GMAP (Wu and Watanabe, 2005) (parameters: -n “*1” -nofails* -x *0*) to detect the possible effect of alternative splicing of miRNA genes. These alignments were interpreted in combination with the “SUmirLocator” outputs to assess the potential of our pipeline in identifying alternative splicing events in of miRNA generation.

The *in silico* expression analysis for putatively identified miRNAs was performed at both mature miRNA and pre-miRNA levels. The miRNA precursors were aligned to the available EST sequences and transcriptome assemblies which were constructed by Trinity software. A detailed summary of alignment dataset is provided in Supplementary Document 3. miRNA precursors which were covered by more than 95% of their length with a > 95% sequence identity were remarked as “*in silico* expressed pre-miRNAs.” The mature miRNAs and miRNA^*^ sequences of *B. distachyon* and *T. aestivum* were also aligned to a set of small RNA sequencing data (Supplementary Document 3) followed by the quality check of small RNA reads by FastQC toolkit (Andrews, 2010) and adaptor removal by Cutadapt software (Martin, 2011). Additionally, the small RNA sequencing reads from *B. distachyon* and *T. aestivum* (PRJNA115065 and PRJNA115065, respectively, obtained from NCBI) were aligned back to identified pre-miRNA sequences by “SUmirFold” with both Bowtie2 (Langmead and Salzberg, 2012) and GMAP (Wu and Watanabe, 2005) in order to show the mapping sites of sRNAs reads on pre-miRNA sequences. The pre-miRNA sequences were utilized to generate an index prior to Bowtie2 and GMAP analyses. sRNA reads were aligned to indexed pre-miRNA sequences in the “local” alignment mode with Bowtie2. GMAP alignment was performed with the “-n 1” and “-x 0”options in order to inhibit the chimeric alignments. Alignment outputs, in *bam* format, were visualized with IGV software (Thorvaldsdóttir et al., 2013) and compared with “SUmirFold” outputs.

Putative miRNAs were further evaluated for detection of TE-miRs and potential siRNA candidates. Putative pre-miRNA sequences identified from all datasets were aligned against a publicly available repeat library of the Poaceae family (MIPS-REdat/Poaceae v9.3p, ftp://ftpmips.helmholtz-muenchen.de/plants/REdat/) which contains 34,135 different repeat sequences (Nussbaumer et al., 2013) RepeatMasker version 4.0.5 (http://www.repeatmasker.org) at default settings. The miRNA sequences aligned to repetitive elements with more than 50% of their lengths were remarked as “TE-miR,” while sequences with perfect complementarity to TEs (up to 3 mismatches allowed) recorded as “potential siRNA candidates.” The distribution of sRNA reads on the precursors, obtained with Bowtie2 and GMAP, were also controlled to support the genuineness of TE-miR and siRNA candidates. When the sRNA reads were detected as concentrated on the predicted mature miRNA and miRNA^*^ locations, similar to sRNA read distribution on miRNA precursors, this accepted as a support for presence of TE-miRs while the dispersed distribution of sRNAs provided support for siRNA candidates.

Putative miRNA targets were also predicted separately for each dataset with the utilization of psRNATarget web-tool (http://plantgrn.noble.org/psRNATarget/) at default parameters (Dai and Zhao, 2011). Coding sequences from *B. distachyon* annotation version 3.1 (downloaded from https://phytozome.jgi.doe.gov/) and *T. aestivum* annotation version 2.2 (downloaded from ftp://ftpmips.helmholtz-muenchen.de/plants/wheat/IWGSC/, The International Wheat Genome Sequencing Consortium, 2014) were used for *in silico* target prediction. Functional annotation of the putative miRNA targets was performed using Blast2GO (http://www.blast2go.com) (Conesa and Götz, 2008). The initial blast step was performed against all non-redundant Viridiplantae (taxid: 33090) proteins (3,485,798) at an e-value cutoff 10^−6^, and the following mapping and annotation steps were carried out at default parameters by Blast2GO. Statistically significant and enriched GO-terms were further selected based on two sided Fisher's exact test outputs which provide the FDR cut-off “< 0.05.” Gene Ontology (GO) terms were also recorded, analyzed, and visualized with multilevel pie graphs. Target enrichment analysis was performed as detailed above, based on annotations, UPE and Expectation values.

To test the small RNA adaptation of pipeline, a subset of sRNA reads from *B. distachyon* (NCBI: PRJNA115065) and whole genome assembly of *B. distachyon* cultivar Bd21 were utilized. The small RNA reads were aligned to high-confidence miRNA list via “SUmirFind_smRNA.pl” allowing 3 mismatches and the aligned part of small RNA reads were trimmed. The trimmed sequences were utilized as the query for second “SUmirFind” process where the genomic encounters of sRNA reads were discarded and utilized with “SUmirFold” for detection of miRNA precursors. Small RNA reads carrying potential miRNA candidates were named based on their homolog partners and previously defined miRNA naming parameters.

## Results

### Identification and comparative analysis of genomic/transcriptomic miRNAs

In order to explore the effectiveness of our pipeline as well as to compare and contrast the impacts of current refinements on *in silico* miRNA identification process, we evaluated all scripts with both genomic and transcriptomic dataset. The genome sequence assembly of *Brachypodium* (version 3.0) was compared against 1404 high-confidence reference miRNAs (Supplementary Document 2) using “SUmirFind” script and a total of 14,379 sequences matched to the reference mature miRNAs with 2 or fewer mismatches (Table 1). Flanking sequences of these candidate miRNAs were evaluated for secondary structure characteristics by “SUmirFold” script and approximately, 36% of these sequences (5152 different hairpin structures where 1062 of them stands as “suspect”) were able to fold into hairpin structures, satisfying initial criteria, including those marked as “suspect” (Table 1). “Suspect hairpins,” the sequences where the miRNA-miRNA^*^ duplexes do not contain any mismatches, were eliminated from this study since both *B. distachyon* and *T. aestivum* have high content of TE elements which may generate false-positive predictions clustered in suspect hairpins. Following the “SUmirPredictor” analysis, a total of 1015 different putative *Brachypodium* miRNA sequences, ~25% of putative hairpin structures passing “SUmirFold,” corresponding to 40 different miRNA families qualified all criteria in the final step of *in silico* miRNA prediction (Table 1, Supplementary Document 4).

**Table 1:** **Summary statistics of miRNA identification and filtering corresponding to four different data sets from ***B. distachyon*** and ***T. aestivum*****.

**Data name**	**Assembly length (Mbp)**	**# of SUmiRFind hits**	**# of SUmiRFold hairpins**	**# of identified different miRNA and miRNA precursor sequences**	**# of corresponding miRNA family**
*B. distachyon* (genome)	~272 Mbp	14,376	4090 (+1062 suspects)	1015	40
*B. distachyon* (transcriptome)	~218 Mbp	9482	1198 (+106 suspects)	87	21
*T. aestivum* (genome)	~17 Gbp	118,100	14,290 (+3116 suspects)	7627	48
*T. aestivum* (transcriptome)	~114 Mbp	5688	265 (+32 suspects)	106	20

The transcriptome assembly of *B. distachyon* cultivar Bd1-1 generated 161 Mbp of total data corresponding to 218,347 contigs where the average contig length was 741 bp. miRNA prediction by “SUmirFind” yielded 9482 matches on 3020 contigs (1.3% of all contigs), which were fold into 1304 hairpin structures including the “suspects” (Table 1). “SUmirPredictor” suggested 81 different miRNA members corresponding to 21 miRNA families (Table 1, Supplementary Document 4). Putative miRNAs identified from both genomic and transcriptomic sequences of *B. distacyon* were comparatively analyzed. Twenty miRNA families out of 21 detected from *B. distachyon* cultivar Bd1-1 transcriptomic data were common with cultivar Bd21 genome. miR444 was only identified from transcriptomic data which may potentially be a cultivar-specific miRNA; however, further analysis for a firm conclusion is necessary. miRNA sequences were also analyzed at the pre-miRNA level by aligning the miRNA precursor sequences which were identified from both datasets. Alignment results showed that 63 pre-miRNA sequences out of 87 (~72% of all identified miRNAs), identified from Bd1-1 cultivar, were similar to Bd21 pre-miRNAs with more than 95% query identity and coverage. Twenty miRNA families identified from Bd21 were not detected at the transcriptome level. Considering the spatiotemporal expression of miRNAs, presence of small portion of miRNAs in both data sets is an expected result.

The enormous genome of hexaploid bread wheat (~17 Gbp) generated 118,100 sequences associated with homolog miRNAs in course of “SUmirFind” process where only 14,290 of them were proceed by “SUmirFold,” At the end of the “SUmirPredictor” process, 7627 miRNA sequences corresponding to 48 miRNA families were identified. On the other hand, Trinity-generated transcriptome assembly of *T. aestivum* spike tissue, 114 Mbp in total length with an average contig length of 456 bp outputted 5688 matches corresponding to 2556 contigs across 251,010 totally generated transcripts on the “SUmirFind” process (1.01% of total transcripts). These sequences were fold into 297 hairpin structures including “suspects” (Table 1). Final evaluation by “SUmirPredictor” suggested the presence of 105 putative pre-miRNA like hairpins, excluding “suspects,” coding for 20 miRNA families (Table 1, Supplementary Document 4). Comparative analysis of miRNAs at the transcriptome and genome level revealed the common presence of 17 miRNA families whereas miR1127, miR162, and miR818 were not detected from genomic data. These sequences either might be false-positive results or plant specific miRNAs. Even though the same cultivar of the wheat were utilized in the analysis process, it is possible that different plants of same cultivar might generate different miRNA families, especially in species where a finished quality genome sequence is not available. The pre-miRNA level comparison of identified miRNAs from both datasets revealed the common presence of 27 miRNAs out of 105 with more than 95% query identity and coverage. Although the relatively small amount of common miRNAs between genomic and transcriptomic data (~25%), all the pre-miRNAs identified from transcriptomic data were detected as identical to genomic miRNA precursors more than 60%.

### Characteristics of putative miRNAs and elimination of false-positive results

The characteristics of the putative mature and pre-miRNA sequences together with pre-miRNA like hairpins were analyzed to control the genuineness of identified mature and pre-miRNA sequences. The average mature miRNA length was observed as ~21 nt. long both across the miRNAs from *B. distachyon* genome and transcriptome where it was detected as 21.8 and 21.5 nt. for *T. aestivum* genome and transcriptome miRNAs, respectively. These results were consistent with previous studies since many of plant mature miRNAs are ranging in 19–24 nucleotide with a bias toward 21 bases in length (Kurihara and Watanabe, 2010; Kurtoglu et al., 2014). Regardless of the length similarity detected in mature miRNAs, pre-miRNAs were identified as highly different from each other respect to length and sequence. The longest putative pre-miRNA identified from *B. distachyon* both genome and transcriptome was pre-miR156 with 328 nucleotides long in length (identified from Bd21 genome) while it was miR1117 in *T. aestivum* genome with 332 nucleotides in length. The average length was observed as 135 nt. long (st.dev = 34.2, median = 128) among putative pre-miRNAs identified from *B. disctachyon* genome while it was 153 nucleotide long (st.dev = 30.5, median = 142) for transcriptomic data. For *T. aestivum* miRNAs identified from genome, the average pre-miRNA length was 112 nt. long (st.dev = 27.86, median = 102) while it was 111 nucleotide long (st.dev = 15.86, median = 117) in transcriptomic data. Minimum folding energy (MFE) and Minimum Folding Energy Index (MFEI) are other important criteria for the determination of miRNA related putative secondary structures. In this analysis, the average MFE value of identified miRNAs for *B. distachyon* genome was −62.61 (st.dev = 15.60, median = −61.7) where MFEI was detected as 1.04 (st.dev = 0.21, median = 0.99). For the miRNAs identified from transcriptomic data of *Brachypodium*, average MFE was detected as −69.19 (st. dev = 11.62, median = −66.1) while the average MFEI was 0.90 (st.dev = 0.13, median = 0.87). Putative pre-miRNAs identified from *T. aestivum* were represented similar values for both of the characteristics; the average MFE values were observed as −63.07 (st. dev = 16.68, median = −59.2) for miRNA identified from genome and −54.13 (st.dev = 13.12, median = −60.5) for transcriptomic miRNAs while average MFEI was observed as 1.38 (st.dev = 0.40, median = 1.26) and 1.13 (st.dev = 0.24, median = 1.02) respectively which show an agreement with previous studies and the property of real miRNA sequences (Axtell, 2013; Kurtoglu et al., 2014).

The final lists of putative miRNAs mined from *B. distachyon* and *T. aestivum* were searched for redundant miRNA ID's deriving from the similarity in mature miRNA sequences of conserved miRNAs presented in the reference miRNA list and three different pairs of redundant miRNA IDs were detected in *B. distachyon* genome while no miRNA with redundant IDs detected among *B. distachyon* transcriptome and both of transcriptomic and genomic data of *T. aestivum*. The most convenient miRNA names for each redundant miRNA IDs was assigned after manual control of similarity between newly identified miRNA precursors and conserved miRNA precursors which were taken from miRBase21. Using blast alignments, “miR156-miR157” doublet was renamed as “miR157”; “miR159–miR319” doublet was changed to “miR319” and “miR482–miR2118” doublet was changed to miR2118 in *B. distachyon* miRNA dataset. Following the control of miRNA IDs, mature miRNAs and their putative precursors were aligned to known non-coding RNAs and organellar genomes of *B.distachyon* and *T. aestivum* with aim of detecting putative false-positive predictions and miRNAs originating from organellar genomes. According to results of blast alignments, any false-positive prediction among putative miRNAs or organellar miRNA was not detected from all the of the miRNA set.

### Genome/transcriptome-wide distribution of putative miRNAs and representation analysis

**Figure 5:**
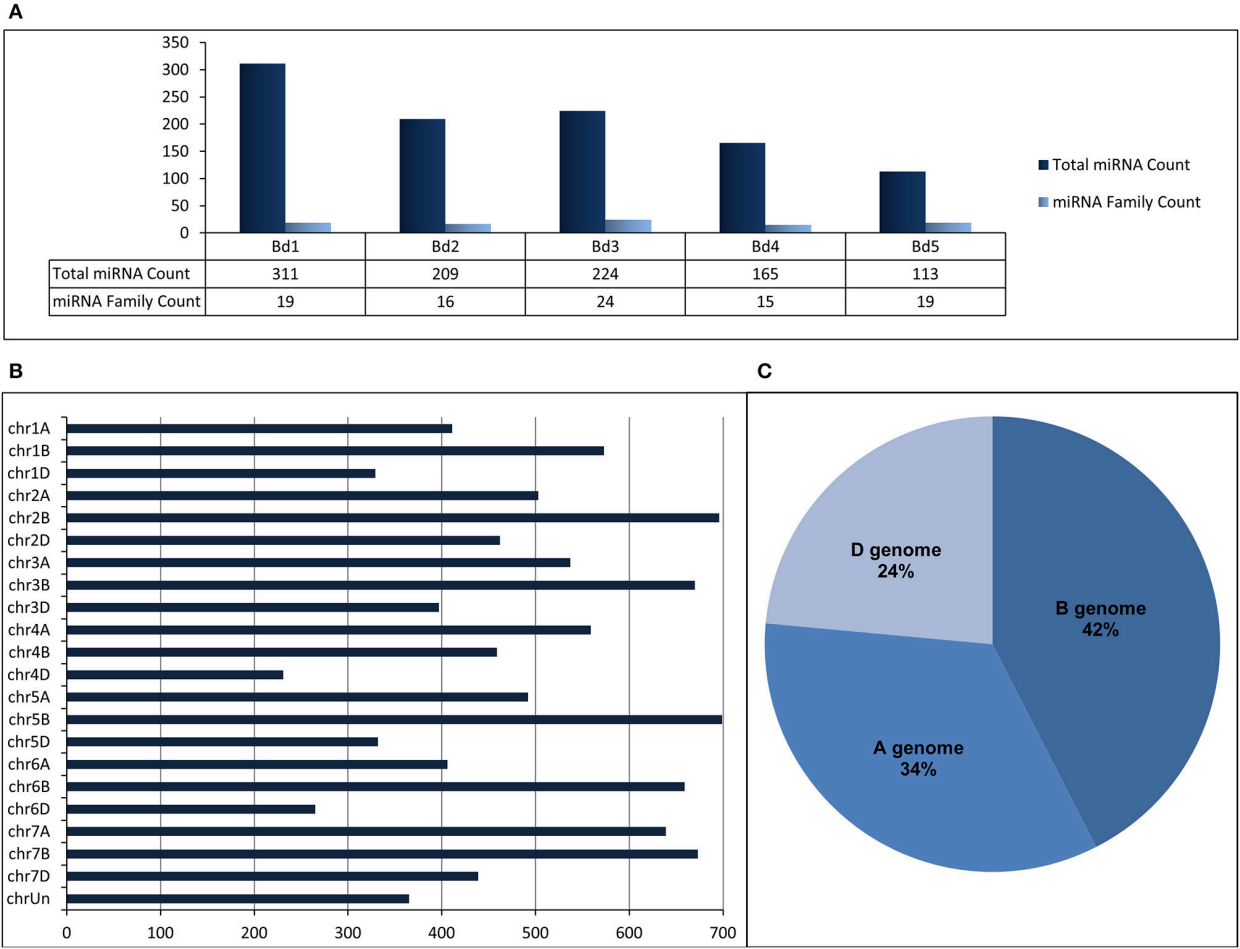
**(A)** Distribution of identified putative miRNAs on different chromosomes of *B. distachyon*. **(B)** Distribution of identified putative miRNAs on different chromosomes of *T. aestivum*. **(C)** miRNA content of each sub-genome of *T. aestivum*.

“SUmirLocator” suggested that many miRNAs identified from *B. distachyon* genome were located on chromosome 1 (311 miRNAs corresponding to 19 miRNA families) while miRNA variety was the highest on chromosome 3 (Figure 5A, Table 2, Supplementary Document 5). The distribution of miRNAs on sense and antisense orientation was almost equal (521 miRNAs on sense direction, 501 miRNAs on antisense direction); however, some miRNAs were likely transcribed from only one orientation. For instance, miR167, miR394, miR397, and miR1127 were only transcribed from antisense direction, while miR319, miR396, miR393, and miR818 were transcribed only from sense direction. Some miRNA families such as miR1122 or miR1128 had multiple coding regions on different chromosomes, whereas others, such as miR319 or miR529 had coding regions exclusive to one chromosome (Table 2). Additionally, miRNA families distributed virtually on all chromosomes were the most highly-represented miRNA families. Remarkably, miR1436, miR1439, and miR1122 were the most highly represented families on the *Brachypodium* genome where their total count corresponded to ~72% of all identified miRNAs. Many different sequences of mature and pre-miRNA associated with these miRNA families were detected across the genome which might be the reason of their high representation (Supplementary Document 5). Additionally, most of the sequences belonging to these highly representative miRNA families were associated with TE elements which might stand as the reason of their high representation.

**Table 2:** **Distribution of miRNA families on the different chromosomes of the ***B. distachyon*** and ***T. aestivum*****.

**Chromosome**	**miRNA Family ID**
Bd1	miR1122, miR127, miR1128, miR1133, miR1135, miR1432, miR1435, miR1436, miR1439, miR160, miR166, miR167, miR169, miR171, miR395, miR396, miR399, miR437, miR528
Bd2	miR1122, miR1128, miR1133, miR1135, miR1139, miR135, miR1436, miR1439, miR156, miR157, miR159, miR164, miR169, miR319, miR399,miR437
Bd3	miR1118, miR1122, miR1128, miR1133, miR1135, miR1136, miR1139, miR1435, miR1436, miR1439, miR156, miR160, miR164, miR166, miR169,miR172, miR2275, miR394, miR395, miR397, miR437, miR529, miR818, miR845
Bd4	miR1122, miR1128,miR1133, miR1135, miR1435, miR1436, miR1439, miR156, miR157, miR166, miR167, miR169, miR2118, miR437,miR818
Bd5	miR1118, miR1122, miR1128, miR1133, miR1135, miR1136, miR1139, miR156, miR157, miR169, miR171, miR2118, miR2218, miR393, miR395, miR399, miR479, miR482, miR530
Tae chr1A	miR1117, miR1118, miR1120, miR1122, miR1128, miR1131, miR1135, miR1136, miR1137, miR1436, miR164, miR166, miR171, miR399, miR9664, miR9666
Tae chr1B	miR1117, miR1118, miR1122, miR1123, miR1125, miR1128, miR1131, miR1133, miR1135, miR1136, miR1137, miR1436, miR164, miR166, miR171, miR399, miR9664
Tae chr1D	miR1117, miR1121, miR1122, miR1125, miR1128, miR1135, miR1136, miR1137, miR1139, miR1436, miR164, miR166, miR171, miR399, miR9664
Tae chr2A	miR1117, miR1118, miR1120, miR1121, miR1122, miR1125, miR1128, miR1131, miR1135, miR1136, miR1137, miR1436, miR169, miR393, miR395, miR399, miR530, miR9666
Tae chr2B	miR1117, miR1118, miR1120, miR1122, miR1123, miR1125, miR1128, miR1130, miR1131, miR1133, miR1135, miR1136, miR1137, miR1436, miR169, miR171, miR393, miR395, miR399, miR437, miR530
Tae chr2D	miR1117, miR1118, miR1120, miR1122, miR1125, miR1131, miR1135, miR1136, miR1137, miR1139, miR1436, miR169, miR393, miR395, miR399, miR530
Tae chr3A	miR1117, miR1118, miR1120, miR1121, miR1122, miR1125, miR1135, miR1136, miR1137, miR1139, miR1436, miR156, miR393, miR399, miR9666, miR9677
Tae chr3B	miR1117, miR1118, miR1120, miR1121, miR1122, miR1128, miR1131, miR1133, miR1135, miR1136, miR1137, miR1436, miR1439, miR156, miR172, miR319, miR437, miR9677
Tae chr3D	miR1117, miR1118, miR1122, miR1135, miR1136, miR1137, miR1138, miR1436, miR1439, miR156, miR399, miR9669, miR9677
Tae chr4A	miR1117, miR1118, miR1120, miR1122, miR1125, miR1128, miR1131, miR1133, miR1135, miR1136, miR1137, miR1436, miR167, miR169, miR171, miR9666
Tae chr4B	miR1117, miR1118, miR1120, miR1121, miR1122, miR1125, miR1128, miR1130, miR1131, miR1133, miR1135, miR1136, miR1137, miR1436, miR169, miR171
Tae chr4D	miR1117, miR1118, miR1122, miR1128, miR1135, miR1136, miR1137, miR1436, miR166, miR167, miR169, miR171
Tae chr5A	miR1117, miR1118, miR1120, miR1122, miR1125, miR1131, miR1135, miR1136, miR1137, miR1139, miR1436, miR156, miR166, miR167, miR169, miR528, miR9666, miR9772
Tae chr5B	miR1117, miR1118, miR1120, miR1121, miR1122, miR1125, miR1128, miR1131, miR1133, miR1135, miR1136, miR1137, miR1436, miR160, miR166, miR167, miR169, miR2118, miR398, miR5062, miR9772
Tae chr5D	miR1117, miR1118, miR1120, miR1121, miR1122, miR1135, miR1136, miR1137, miR1138, miR1436, miR1439, miR156, miR160, miR166, miR167, miR169, miR398, miR9772
Tae chr6A	miR1117, miR1118, miR1121, miR1122, miR1131, miR1135, miR1136, miR1137, miR1436, miR156, miR160, miR394, miR396, miR397, miR9666, miR9670
Tae chr6B	miR1117, miR1118, miR1120, miR1121, miR1122, miR1125, miR1128, miR1131, miR1133, miR1135, miR1136, miR1137, miR1436, miR160, miR164, miR394, miR396, miR397, miR9663
Tae chr6D	miR1117, miR1118, miR1122, miR1125, miR1131, miR1135, miR1136, miR1137, miR1436, miR156, miR160, miR164, miR394, miR396, miR9662, miR9670
Tae chr7A	miR1117, miR1118, miR1121, miR1122, miR1123, miR1125, miR1131, miR1135, miR1136, miR1137, miR1436, miR160, miR169, miR2275, miR396, miR399, miR9666
Tae chr7B	miR1117, miR1118, miR1122, miR1125, miR1128, miR1131, miR1135, miR1136, miR1137, miR1436, miR160, miR166, miR169, miR396, miR399
Tae chr7D	miR1117, miR1118, miR1120, miR1121, miR1122, miR1125, miR1131, miR1135, miR1136, miR1137, miR1436, miR160, miR166, miR169, miR2275, miR399
Tae chrUn	miR1117, miR1121, miR1122, miR1128, miR1131, miR1133, miR1135, miR1136, miR1137, miR1436, miR169, miR171, miR399, miR9666

Regarding to miRNA identification results of *T. aestivum*, miR1117, miR1122, and miR1436 were detected as the miRNAs families with the most members across the genome with 4003, 3241, and 1037 miRNA isoforms, respectively (Supplementary Document 5). Most of the miRNAs were detected on chromosome 5B (699 miRNA associated region) which is followed by chromosome 2B (696 miRNA associated region) and 7B (673 miRNA associated region) (Figure 5B). Also, many miRNAs were identified as coming from B genome (4429 miRNA associated region) while the D genome has the least miRNA associated region with (2455 miRNA associated region) (Figure 5C). Additionally, it was observed that the miRNA distribution on sense and antisense strands is almost equal (5402 miRNAs on sense and 5394 miRNAs on antisense), similar to *Brachypodium* miRNAs.

**Figure 6:**
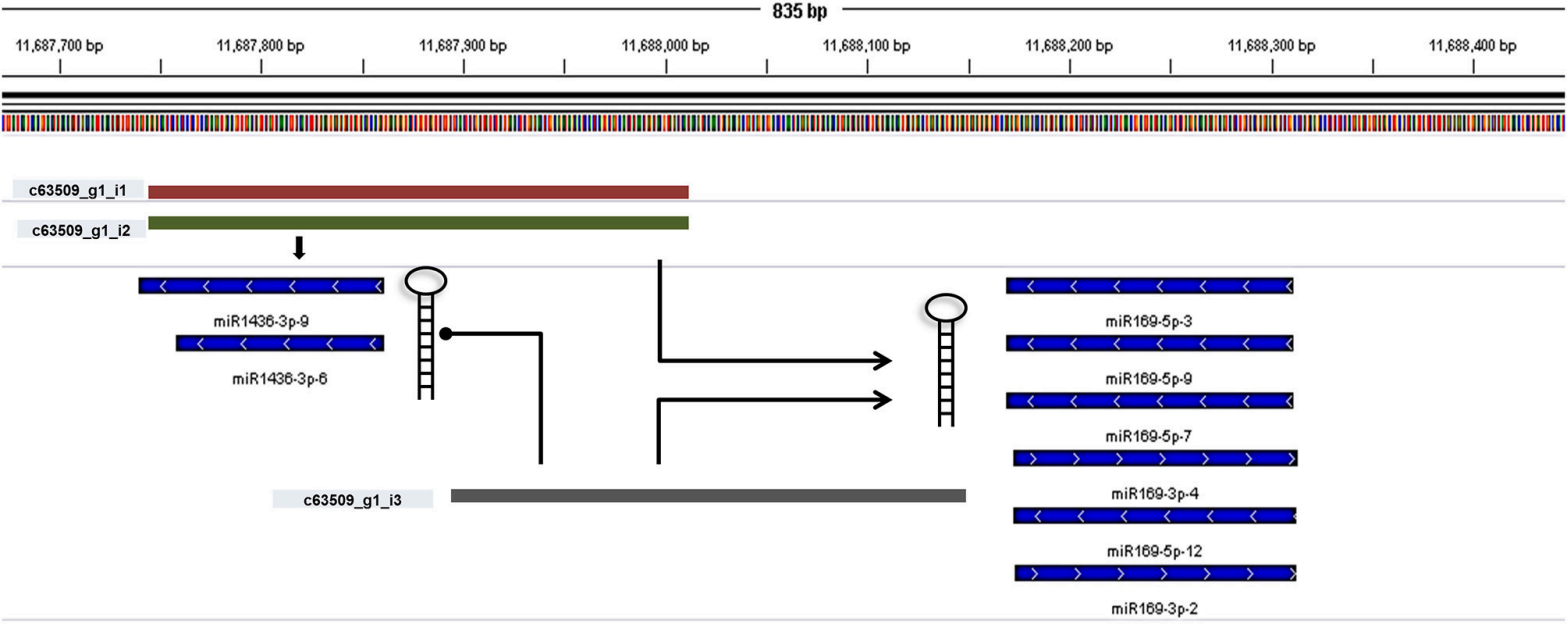
**Alternative splicing of miRNA precursors**. miRNA genes might get through alternative splicing and the spliced variants might generate different miRNA precursors. In order to understand such effects of alternative splicing, miRNA precursor identified from transcriptomic data were aligned back to genome with GMAP and alignment results were visualized with IGV. In this example, three different contigs can be transcribed from the same genomic region of *Brachypodium* genome (c63509_g1_i1, c63509_g1_i2, and c63509_g1_i3). The generation of isoform 1 and 2 leads to formation of miRNA members from both miR169 and miR1436 families. If the isoform 3 is produced, the miR1436 sequences cannot be generated from this transcript.

miRNA identification from *Brachypodium* transcriptome suggested that miR169, miR156, and miR166 family members were the most representative miRNAs where their precursors detected as generated from many different contigs (Supplementary Document 5). Closer examination of miRNA distribution on different isoforms of same contigs demonstrated the effect of possible alternative splicing events on miRNA generation. For instance, contig named as “c63509” associated with two different miRNA families: miR169 and miR1436 (Figure 6, Supplementary Document 5). The first and second isoforms of this transcript resulted in the generation of both miRNA families while the third isoform only associated with miR169 family members. In order to validate this result, the miRNA precursors obtained from *B. distachyon* transcriptome were aligned to genome with GMAP and alignment results confirmed that these miRNA precursors are coming from the same genomic region (Supplementary Document 5). In some cases, it was detected that alternative splicing is not effective on miRNA sequence. miRNA results from contig “c72093” suggested that 8 different isoforms of same gene resulted in the same miRNA sequence (miR156-3p-1: GCUCACUCCUCUUUCUGUCAGC) (Supplementary Document 5). These results suggest that possible alternative splicing events might be effective on generation of different miRNA varieties which can be detected by examination of “SUmirLocator” outputs. Additionally, the genomic locations of *Brachypodium* transcriptome miRNAs, identified with GMAP, and genomic locations of miRNAs identified from genome, detected with “SUmirLocator” were compared in order to see the similarity between identified miRNAs from genome and transcriptome in more detail. Sixty-two (71% of all identified miRNAs) of *Brachypodium* transcriptome miRNAs were detected as generated from exact same or near same location with their genomic partners. The miRNA sequences which were identified from transcriptomic data but not detected as coming from the same genomic location with their genomic partners might still stand as genuine miRNA candidates; however, they might be encoded from different locations since the genomic miRNAs detected from cultivar Bd21 while the transcriptomic miRNAs were detected from cultivar Bd1-1.

In the transcriptome data of *T. aestivum* spike, the most represented miRNAs were detected as miR1436, miR1122, and miR1130 which approximately correspond to 51% of all identified miRNAs (Supplementary Document 5). Interestingly, miR1117 was not detected as represented with a high number, likewise the result for identified miRNAs from genome. At the end of “SUmirPredictor” process, only 86 of contigs were associated with putative miRNA precursors. In most of cases, more than one miRNA were detected as located on the same transcriptomic contig where the highest number of associated miRNA count within one contig was 5. miRNA sequences which are detected as originating from same contig were generally associated with the same miRNA precursor. In some cases, transcription of same contig from different directions resulted in the generation of different miRNAs which might represent the importance of transcription direction for miRNA biogenesis. It was also noticed that different isoforms of same contig generated by Trinity were associated with different miRNA families. Additionally, similar to *B. distachyon* miRNAs, some miRNAs detected as transcribed from only sense direction (e.g., miR167, miR1128, and miR1131) while some of them were generated from only antisense direction (e. g., miR1125, miR1127, and miR1133) (Supplementary Document 5).

The miRNA location analysis on the *T. aestivum* transcriptome also provided insights about genomic and transcriptomic organization of different miRNA genes. Some members of miR1135 and miR1136 families were detected as proceed from the same transcript (Supplementary Document 5). Additionally, in some cases, different isoforms of same transcript were associated with different miRNA sequences. Across the miRNAs identified from *T. aestivum* transcriptome; miR1120, miR1122, miR1128, miR1130, and miR1436 were detected as relatively located in a close proximity to each other while some of these miRNAs were associated with the same precursor sequence (Supplementary Document 5). To analyse the genomic organization of miRNAs in detail along with the effect of possible alternative splicing events in miRNA genes in wheat, miRNAs identified from transcriptomic data were aligned back to genome with GMAP. miRNAs were aligned to each chromosome separately and alignment result showed convenience with “SUmirLocator” results of *T. aestivum* for the miRNAs which are identified from both genome and transcriptome. Many miRNAs were detected as distributed on several chromosomes while some of them clustered on particular ones. For instance, miR167 family members were detected as located on chromosome three regarding to both GMAP and “SUmirLocator” alignment results. Additionally, comparisons of different alignment outputs also confirmed the possible effect of alternative splicing event on *T. aestivum*. As an example, “SUmirLocator” suggested that possible alternative splicing event on the region which code for contig “c195255” might have an effect on miRNA variety. First isoform give rise to generation of a specific sequence belongs to miR1439 while this miRNA is not detected on second and third splicing isoforms (particularly in the chromosome 2A) (Supplementary Document 5). Genomic alignment of this miRNA confirmed that this entire miRNA precursor is transcribed from same region and possible alternative splicing can affect the miRNA variety. Overall, the analysis of both genomic and transcriptomic locations of identified miRNAs provided insights to the organization of miRNA genes and their regulation at the transcriptional level.

### miRNA expression analysis

*In silico* miRNA expression analysis provides evidence that the computationally identified miRNAs are likely expressed, thereby supporting the genuineness of the respective miRNA. For pre-miRNA expression evidence, newly identified putative miRNA precursors were aligned to known EST sequences together with constructed transcriptome assemblies (Supplementary Document 3), which suggested that expressed precursor sequences likely exist for 22 miRNA families identified from *Brachypodium* genome while all miRNA precursors were detected as expressed for transcriptome miRNAs. Since the pre-miRNA sequences identified from transcriptomic data are coming from genuine transcripts, the presence of *in silico* expression evidence for all transcriptomic miRNAs is an expected result. At the mature miRNA level, 596 and 437 of genomic *Brachypodium* mature miRNA and miRNA^*^ sequences were aligned against sequence reads coming from small RNA libraries (Supplementary Document 3) with 100% query identity and coverage, respectively with at least 3 reads (Supplementary Document 6). Among these, 374 mature miRNA/miRNA^*^ duplexes had expression evidence both for miRNA and miRNA^*^ and, thus, accepted as “*in silico* expressed.” For *Brachypodium* transcriptome miRNAs, expressed mature miRNA count was detected as 49 (56% of all identified miRNAs) corresponding to 13 miRNA families. The miRNA sequences for which expression evidence could not be obtained with the currently available expression data may still be expressed under highly specific conditions or at specific tissues or developmental stages.

Across more than 7000 miRNAs identified from the *T. aestivum* genome, 3867 of them showed corresponding to 40 miRNA families were detected as “*in silico* expressed” at mature miRNA level while this number 2148 at the pre-miRNA level. Of the 102 different mature miRNA/precursor pairs identified from *T. aestivum* spike transcriptome, 37 (36% of all identified miRNAs) miRNA precursors correspond to 14 miRNA families were detected as computationally expressed by aligning to ESTs and contigs of transcriptome assemblies with >95% query identity and query coverage, distinctly from the *B. distachyon* transcriptome miRNAs where the utilized data for pre-miRNA expression contains higher amount of sequences. Additionally, 83 of mature miRNA sequences together with 90 of miRNA^*^ sequences were aligned to small RNA sequencing reads (Supplementary Document 3) with 100% query identity and coverage. Across all identified miRNAs from *T. aestivum* spike transcriptome, 73% of them corresponding to 75 miRNA/miRNA^*^ duplex were detected as “*in silico* expressed” at the mature miRNA level (Supplementary Document 6).

**Figure 7:**
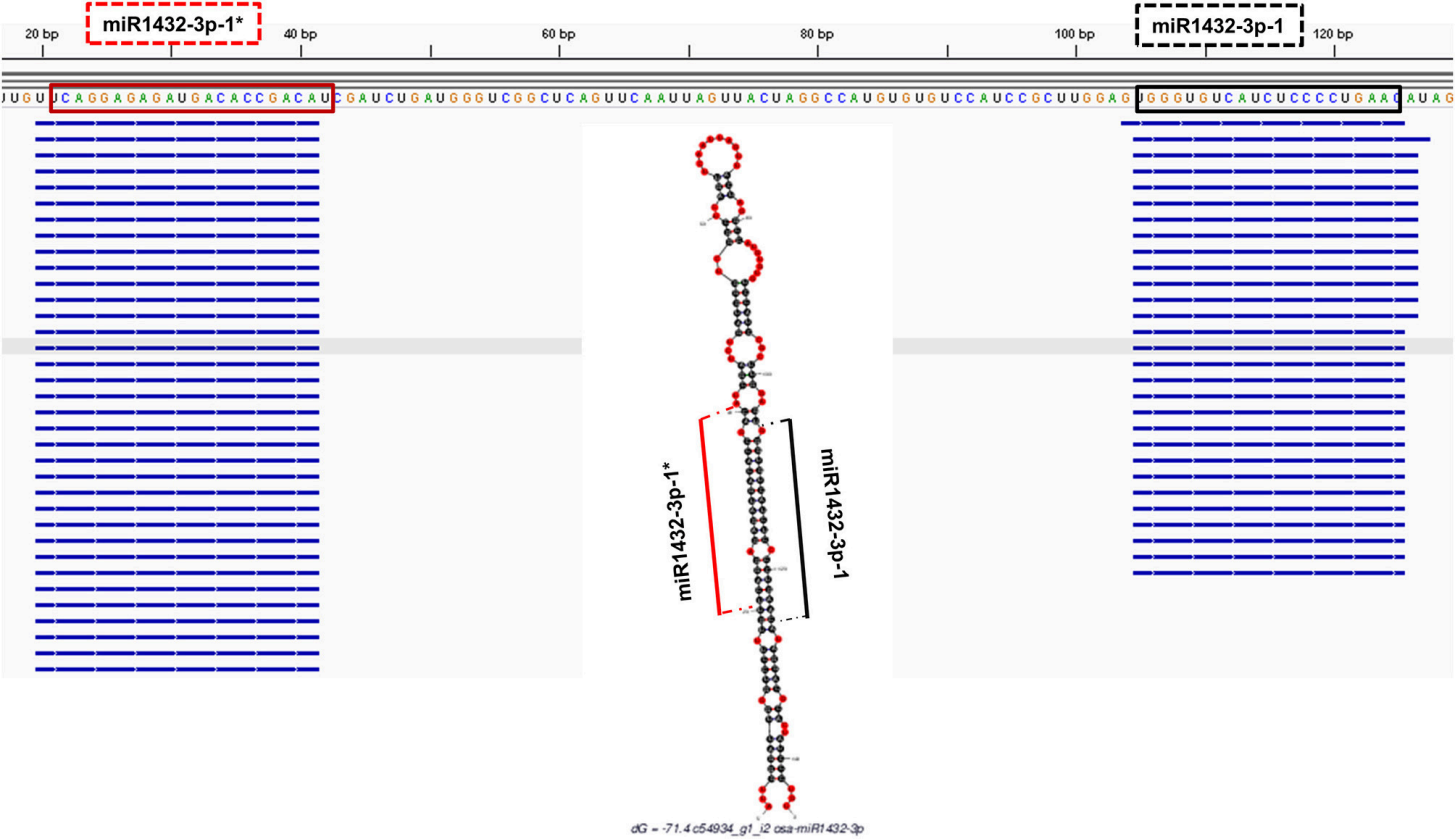
**Alignment of small RNA sequencing data to pre-miRNA**. The small RNA sequencing reads are aligned to the predicted pre-miRNA sequences to show the efficiency of “SUmirFold” and “SUmirPredictor” for detection of mature miRNA/miRNA^*^ pairs on the miRNA precursors. Alignment result from GMAP and Bowtie2 which are visualized with IGV showed that small RNA reads were successfully aligned to predicted locations of mature miRNA/miRNA^*^ pairs for miR1432-3p-1 from *B. distachyon*. This analysis can be used for inspection of genuineness of miRNA precursor sequences in an addition to *in silico* pre-miRNA expression analysis expression analysis.

In order to check the genuineness of identified pre-miRNAs, small RNA reads from *Brachypodium* and *T. aestivum* were aligned back to pre-miRNAs with both Bowtie2 and GMAP and alignment result were compared to predicted location of mature miRNA/miRNA^*^ duplexes via “SUmiRFold.” Approximately, 74% of pre-miRNAs identified from genome and 68% of pre-miRNAs detected from transcriptome of *Brachypodium* were aligned back to sRNA reads. For *T. aestivum*, only 29% of miRNAs identified pre-miRNAs from genome were covered by sRNA reads while this percentage was 80% for transciptomic miRNAs. sRNA reads were detected as aligned the exact same location with the one predicted by “SUmirFold” (Figure 7) for many miRNAs and proved the effectiveness of “SUmirFold” for determination of miRNA locations of the precursor sequences. The miRNAs which are not covered or aligned to sRNA reads may still stand as genuine miRNAs since the alignments result are directly correlated with number of sRNA reads used in the analysis. Additionally, some miRNAs, particularly identified from genome may not be expressed under given condition; however, their expression might occur in some other specific conditions. Overall, these results showed that this pipeline able to identify genuine miRNAs in a precise manner.

### Identification of TE-miR

Repetitive elements including transposable elements (TEs) can constitute up to 80–90% of plant genomes (Feschotte et al., 2002). In order to detect association of predicted *Brachypodium* and *Triticum* miRNA sequences with TEs, pre-miRNA sequences were aligned to known plant TEs from Poaceae repeat library which contains 34,135 sequences. Overall, 81% of *Brachypodium* miRNAs identified from genome precursors contained TEs with more than 50% of their lengths and termed as TE-MIRs (Supplementary Document 7). Among these, 90 pre-miRNA sequences were aligned to TEs with an almost perfect complementary and classed as “potential siRNA candidates” (Supplementary Document 7). Across putative TE-miRs of *Brachypodium* genome, more than 200 of these sequences were detected as “*in silico”* expressed at mature miRNA level while only 23 of siRNA candidates were observed to have expression evidence. TE-miR content of *Brachypodium* transcriptomic miRNAs were relatively lower; only 38 miRNA precursors were detected as “TE-miRs” while 9 of them separated as potential siRNA candidates. All the siRNA candidates were detected as “*in silico* expressed” at mature miRNA level while 37 of TE-miRs showed the expression evidence (Supplementary Document 7).

The content of TE-miRs detected as higher in *T. aestivum* in both genome and transcriptome. Approximately, 95% of all identified miRNAs were detected as TE-miRs since they contained TE elements with more than 50% of their lengths. Of these, 74% of TE-miR candidates aligned to TE elements with 3 or fewer mismatches and were grouped as siRNA candidates. The expression analysis of these miRNAs showed that 3634 putative TE-miRs were detected as expressed at mature miRNA level while 2712 of them were detected as siRNA candidates (Supplementary Document 7). Additionally, 98 out of 105 miRNAs identified from *T. aestivum* transcriptome were detected as “TE-miR” candidates while 30 of them represented siRNA potential. Across TE-miR candidates of *T. aestivum* transcriptome miRNAs, 68 of them showed *in silico* miRNA expression evidence while all the siRNA candidates were detected as putatively expressed. In addition to a number of hypotheses on the mechanisms of plant miRNA origins, such as inverted-duplication and spontaneous evolution, TEs can also contribute the evolution of miRNAs (Voinnet, 2009). Thus, it is possible to associate the high abundance of TEs across miRNA precursors with the evolutionary source of plant (Li et al., 2011; Kurtoglu et al., 2013; Alptekin and Budak, 2016). On the other hand, it is highly challenging to differentiate between TE-miRs and putative siRNAs (Piriyapongsa and Jordan, 2008). Thus, the siRNA candidates might be actual TE-miRs, however, further analysis are necessary to make this discrimination.

**Figure 8:**
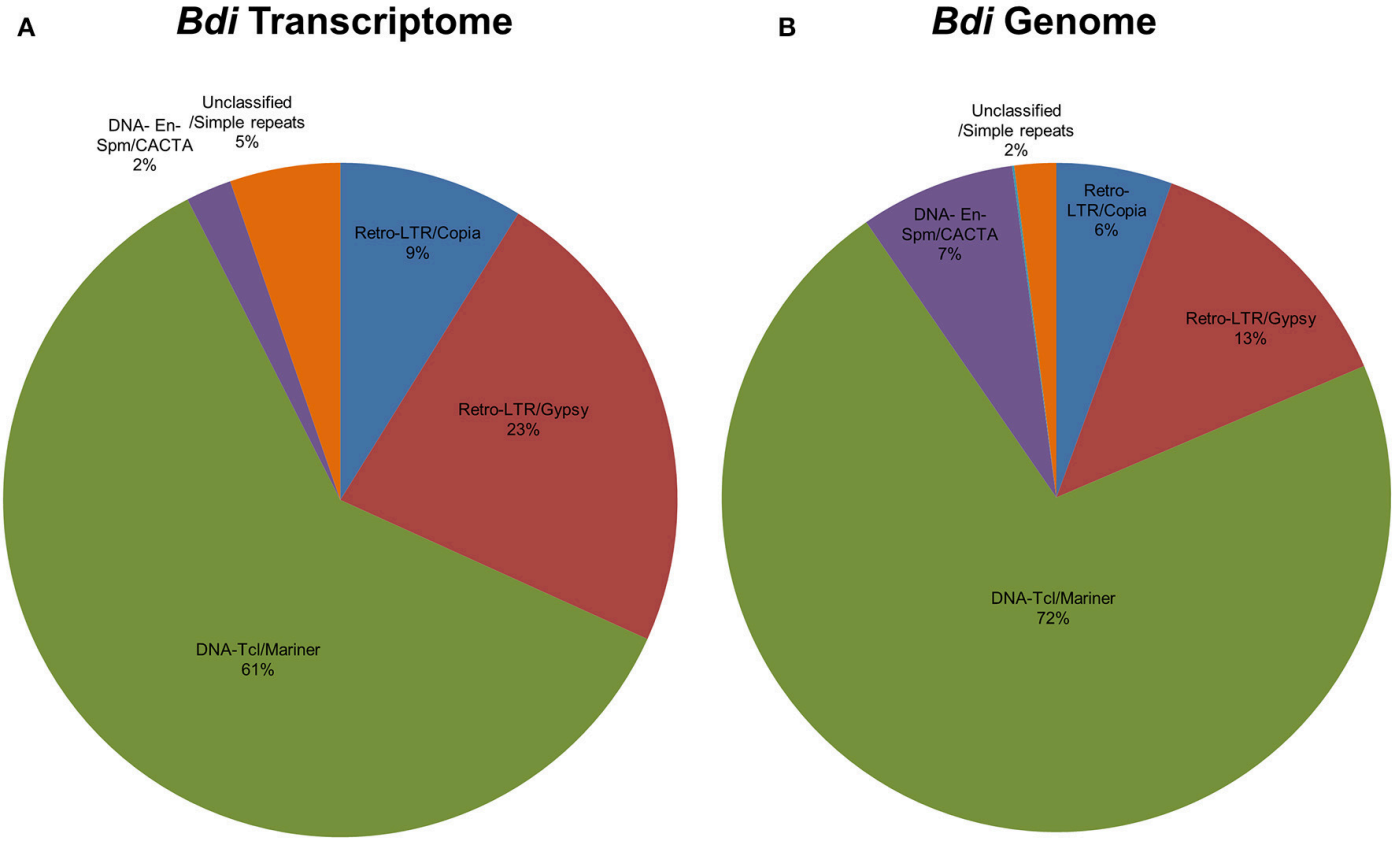
**Distribution of TE elements families on ***Brachypodium*** pre-miRNA sequences. (A)** Distribution of TE element families on *B. distachyon* transcriptome miRNAs. **(B)** Distribution of TE element families on *B. distachyon* genome miRNAs.

**Figure 9:**
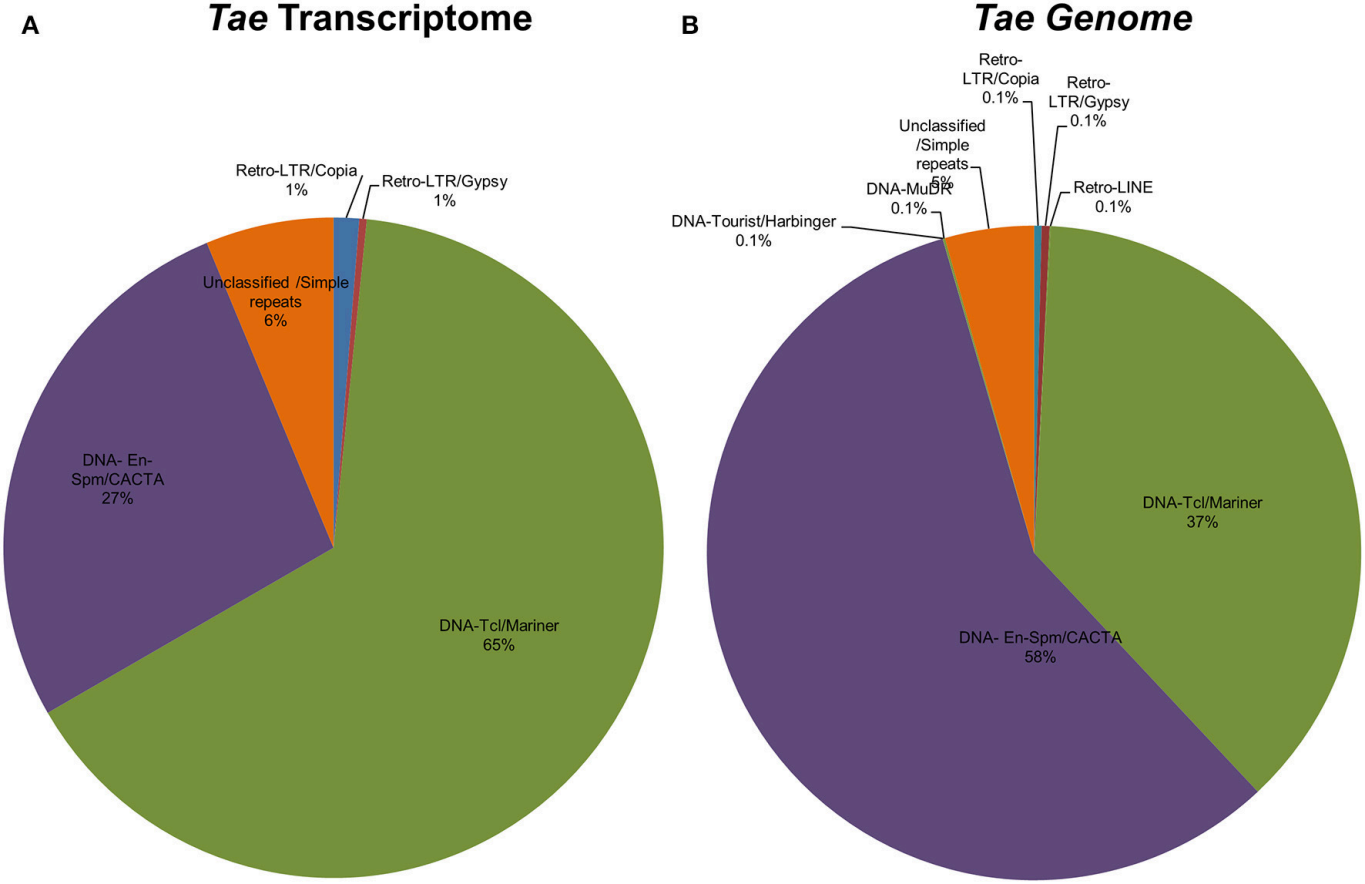
**Distribution of TE elements families on ***T. aestivum*** pre-miRNA sequences. (A)** Distribution of TE element families on *T. aestivum* transcriptome miRNAs. **(B)** Distribution of TE element families on *T. aestivum* genome miRNAs.

In this analysis, both Class I Retro-elements and Class II DNA transposons were observed across all putative precursor sequences, with DNA transposons being remarkably abundant (Supplementary Document 7, Figures 8A,B, 9A,B). Tcl/Mariner DNA transposon family were detected as covering many TE-miRs in *B. distachyon*, both at genome (Figure 8B) and transcriptome level (Figure 8A), while En-Spm/CACTA was the mostly found TE family in *T. aestivum* miRNAs identified from genomic data (Figure 9B). Additionally, the miRNA families associated with TEs were further analyzed and miR1122, miR1436, and miR1439 family members were detected as the miRNA families which has the most members of TE-miRs. On the other hand, majority of non-TE-miRs belongs to miR393, miR394, miR395, miR397, miR399, miR529, miR530, miR845, miR221, and miR2175 families in *B. distachyon* and to miR162, miR167, and miR1118 families in *T. aestivum*.

**Figure 10:**
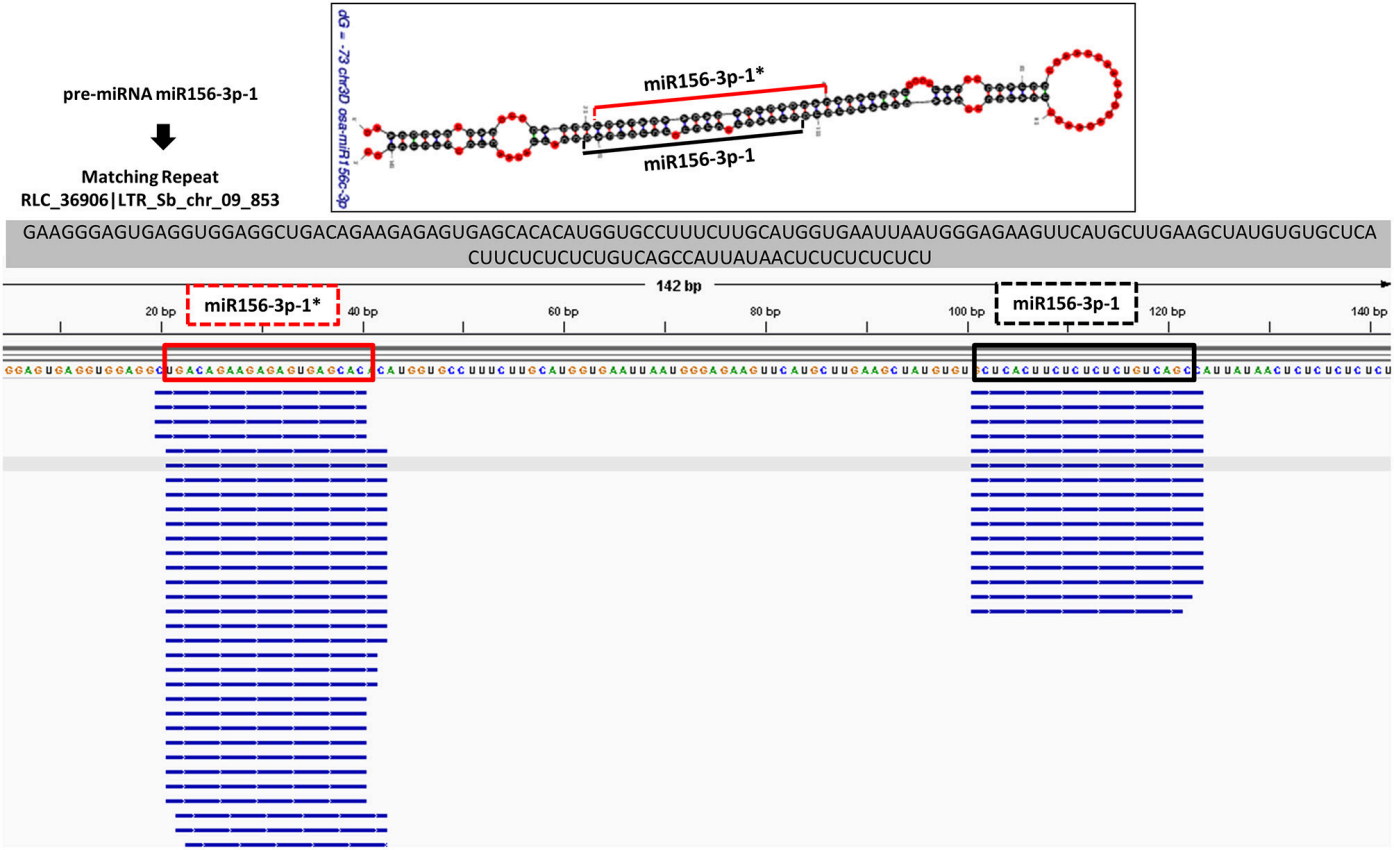
**Distribution of sRNA reads on putative TE-miR**. miR156-3p-1 from *T. aestivum* genome is a TE-miR candidate which aligned to TE element “RLC_36906|LTR_Sb_chr_09_853” with more than 50% of its length. The distribution of sRNA reads are concentrated on regions where the mature miRNA and miRNA^*^ sequences were predicted as located by “SUmirFold” (mature miRNA is between 101 and 122th (marked with black square) and star sequence is between 21 and 40th bases (marked with red square).

**Figure 11:**
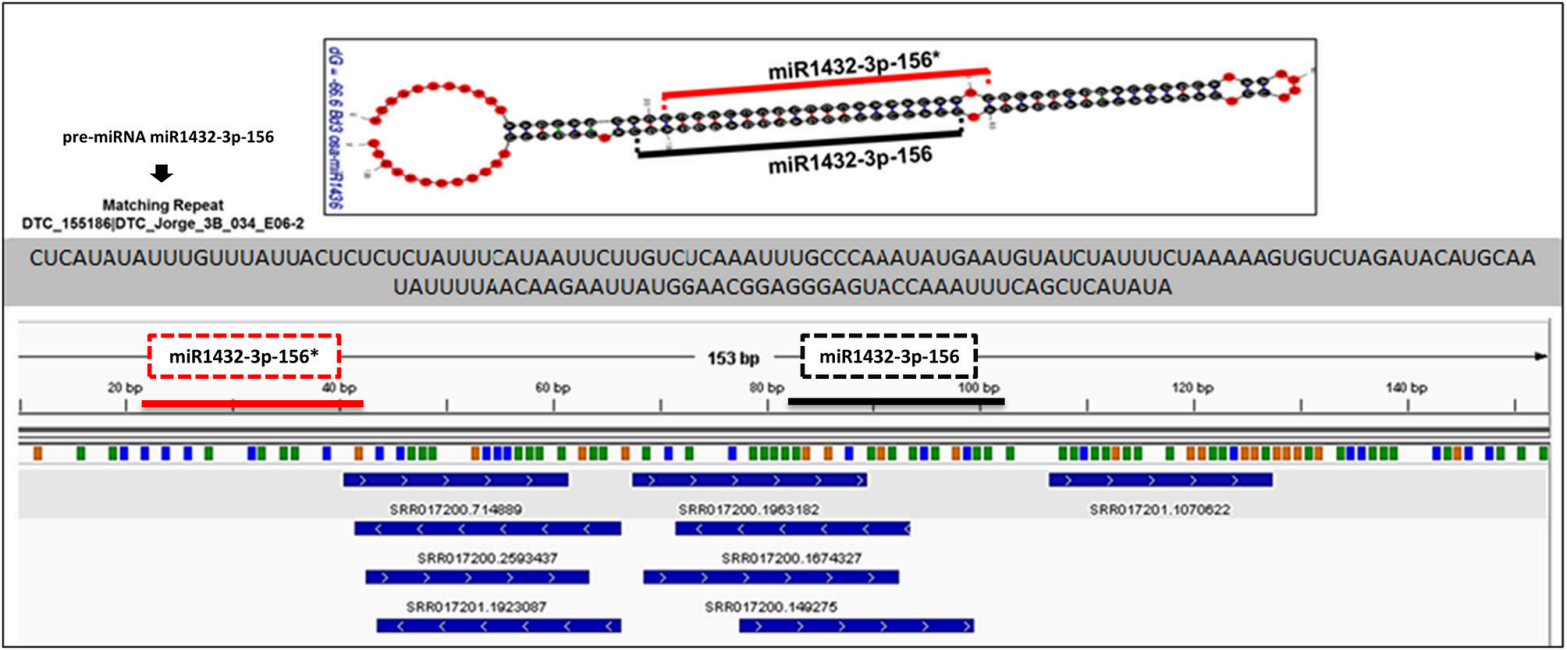
**Distribution of sRNA reads on putative siRNA candidates**. miR1436-3p-156 from *B. distachyon* genome is a siRNA candidate which shows similarity to TE element “DTC_155186|DTC_Jorge_3B_034_E06-2” and the dispersed distribution of sRNA reads on the precursor supports the genuineness of this predicted siRNA. Instead of mature miRNA (marked with black sticks, from 82 to 102th bases) and miRNA^*^ locations (marked with red sticks, from 21 to 41th bases), sRNA reads are dispersed along the precursor.

The distribution of sRNA reads on the precursors of TE-miRs and siRNA candidates were also analyzed in order to further support the genuineness of these candidates. In the majority of the TE-miRs, the sRNA reads were detected as concentrated on the predicted mature miRNA and miRNA^*^ locations (Figure 10). Many siRNA candidates were also supported by relatively even distribution of sRNA reads on the precursors where there is sufficient sRNA sequence data (Figure 11). It is a known phenomenon that siRNAs target the TE elements and suppress their expression in order to maintain the genomic stability (Ito, 2012). Dispersed distribution of sRNA reads on the precursors of siRNA candidates provides evidence for this kind of a silencing mechanism.

### Putative miRNA targets and their enrichment

miRNAs regulate gene expression by binding on the complementary sites of target mRNAs and suppressing their expression through translational inhibition or mRNA decay/cleavage (Fahlgren and Carrington, 2010). Thus, identification of target transcripts of miRNAs provides information about their functional role at the cellular level. Putative targets of predicted miRNAs represented a diverse distribution in *T. aestivum* and *B. distachyon* (Supplementary Document 8). Some miRNAs suggested having important regulatory roles for plant metabolism. For instance, miR397 from *Brachypodium* genome suggested that it is targeting “probable magnesium transporter NIPA8 isoform X1” which might have an essential role for magnesium metabolism for plant together with the enzymes which utilize this element. In another example, miR160 from *T. aestivum* suggested that it is targeting “Auxin response factor 22” which is an important factor in hormone signaling in plants. Such miRNA-target pairs provide a rough idea for the detection and selection of functionally important miRNA families for validation among the pool of putatively identified miRNAs.

Target enrichment analysis for known proteins helped the key roles of several miRNAs in a more accurate way and easier elimination. For instance, 1107 different miRNA associated targets were decreased to 160 for *B. distachyon* genome miRNAs. Target processing mode (cleavage and inhibition) of most of the enriched targets were associated with “cleavage” where the target transcript destroyed by cleavage of the mRNA transcript. Multiplicity is a value which is given by psRNATarget and shows the different target binding sites for miRNAs (Dai and Zhao, 2011). Many enriched targets associated with only one target binding site and their multiplicities were counted as 1. Regarding to enriched target analysis results, highly represented miRNAs suggested enriched targets with key roles in essential molecular pathways (Table 3, Supplementary Document 8). One of the mostly represented miRNAs identified from *B. distachyon* genome, miR1436, detected as targeting “methyltransferase 6 isoform X2” and “WAT1-related At5g64700-like” proteins are the most enriched targets. Also, some other miR1436 family members were detected as targeting “Dead-Box Associated Protein,” “Heat Shock Protein,” “DNA Excision Repair Associated Protein” and “Early Dehydration Responsive Protein,” suggesting crucial involvement in stress responses (Supplementary Document 8). On the other hand, miR1122, one of the most highly represented miRNAs for both *T. aestivum* and *B. distachyon*, was suggested that targeting “Pre-mRNA-processing-splicing factor 8” regarding to annotation of *T. aestivum* CDS even though the most enriched target of this miRNA family was detected as “PREDICTED: Uncharacterized protein LOC100837429 isoform X1” regarding to *Brachypodium* annotation. Target analysis and enrichment also revealed that miRNAs which are generated as a result of alternative splicing are targeting different proteins. For instance, the miRNAs which are generated different isoforms of from *T. aestivum* “c195255_g1” associated with different targets. The miR1439-3p-4 which the one expression is lost in the second and third isoforms associated with “KH domain-containing” protein while miR1436-3p-8 and miR1436-3p-1 were associated with “potassium transporter 13” and miR1130-5p-10 is associated with “Tropinone reductase.”

**Table 3:** **The most enriched known targets of mostly represented miRNAs from ***B. distachyon*** and ***T. aestivum*****.

**miRNA ID**	**Predicted most enriched known target**
miR1117	NA
miR1122	Pre-mRNA-processing-splicing factor 8, Uncharacterized protein LOC100837429 isoform X1
miR1130	Tropinone reductase, Kinesin KIF15
miR1436	Methyltransferase 6 isoform X2, WAT1-related At5g64700-like protein, Calcium-dependent kinase
miR1439	Uncharacterized protein LOC100824126, Weak chloroplast movement under blue light 1-like, Ubiquitin carboxyl-terminal hydrolase 27 isoform X1
miR156	Squamosa promoter-binding 3
miR166	Uncharacterized protein LOC106866306
miR169	Uncharacterized protein LOC100822852 isoform X1, Probable transport Sec1a isoform X2

Statistical enrichment of GO-terms was performed by utilizing Fisher's exact test method for each miRNA family in each dataset (Supplementary document 8). The most significant GO-terms was chosen based on FDR cut-off < 0.05 and outputs showed some essential regulatory features of miRNAs in several pathways. Interestingly, no enriched GO term was detected for the *T. aestivum* transcriptome miRNAs with the given cutoff. Several miRNA families from *T. aestivum*; miR166, miR169, miR171, miR2275, miR397, miR5062, miR9662, miR9663, and miR9664; associated with “kinase activity” which may suggest their roles in cellular signaling. No information is detected about the specific kinase family which these miRNAs are regulating even the presence of several association with miR1429 and miR531 with MAPK kinase pathway in rice (Raghuram et al., 2014). Enriched GO-terms for miR397 from *B. distachyon* suggested its association with stress response based on the presence of GO-terms such as “response to stress,” “response to external stimulus,”' and “response to biotic stimulus.” Additionally, miR167 from *T. aestivum* was associated with “pollen-pistil interaction” suggesting its regulatory role in development. This miRNA family was also associated with the young tissues such as shoot-tips and flowers in *Arabidopsis* (Glazińska et al., 2014), therefore, further elucidation of its function in *T. aestivum* might be important for understanding the developmental regulations.

### Small RNA sequencing adaptation of miRNA pipeline

In order to detect the feasibility of our method to small RNA sequencing data, a small portion of sRNA reads from *B. distachyon* (200,000 sRNA sequences) was used as a trial data. Following the adaptor trimming of these reads, they were aligned to high-confidence miRNA list allowing up to 3 mismatches and these alignments resulted in 17,434 blast hits (Supplementary Document 9). The small RNA reads which were detected as similar to known miRNA sequences were trimmed at locations where they aligned to known miRNA sequences. Trimmed sequences were aligned back to *Brachypodium* genome with “SUmirFind” and their hairpin sequences were detected with “SUmirFold.” SUmirFold generated 740 positive hits were the small RNA reads were aligned back to genome and 144 of this locations folded into hairpin-shaped miRNA precursors (Supplementary Document 9). Processing of these sequences with “SUmirPredictor” resulted with 82 miRNA sequences corresponding to 16 miRNA families. These miRNA sequences were also compared to the miRNAs detected from *Brachypodium* genome. Seventy-seven pre-miRNA sequences identified from sRNA data were aligned to miRNA precursors identified from *B. distachyon* genome with 100% query coverage together with more than 70% identity and only five of the miRNAs identified from small RNA reads were not detected across *Brachypodium* genome miRNAs (miR1122-3p-8, miR1122-3p-9, miR1122-3p-10, miR1127-5p-1, and miR160-3p-1) (Supplementary Document 9).

## Discussion

In recent years, intensified focus on miRNA research has resulted in the generation of many different pipelines and software for the identification of miRNAs (Jones-Rhoades and Bartel, 2004; Kleftogiannis et al., 2013). Previously, we have developed an automated pipeline consisting of two consecutively run scripts, “SUmirFind” and “SUmirFold” for *in silico* plant miRNA identification from large-scale sequencing data (Lucas and Budak, 2012), which helped unlock many potential miRNA species from *Triticeae* family members (Akpinar et al., 2015; Akpinar and Budak, 2016; Alptekin and Budak, 2016). Herein, we refined our pipeline with the implementation of two additional scripts by taking aim at providing increased sensitivity and specificity in course of homology-based *in silico* miRNA identification. In the virtue of current refinements, this methodology provides mining of miRNAs from genomic and transcriptomic data in a sensitive manner together with their detailed annotation and characterization. Additionally, we showed that this pipeline can be adapted to small RNA sequencing data by incorporation of a few additional steps.

Utilization of an accurate reference miRNA set is crucial for both homology and machine learning based miRNA mining. Reference miRNA set selection is also important in the process of miRNA identification from small RNA sequencing data since many pipelines for small RNA data processing require a list of known miRNA sequences (Kang and Friedländer, 2015; Tam et al., 2015). Unfortunately, the genuineness of whole miRNAs in the miRBase, which is the most comprehensive miRNA database, is skeptical, because; many miRNA sequences lack experimental evidence and mainly identified with *in silico* methods (Meng et al., 2012; Kozomara and Griffiths-Jones, 2014). Using *in silico* identified miRNAs in the process of computational miRNA mining may not generate reliable results since the genuineness of *in silico* miRNAs is not certain. As a suggestion to this issue, a set of high-confidence miRNAs were release in the latest version of miRBase; however, some other problems has arisen, in this case (Kozomara and Griffiths-Jones, 2014). One of the major constraints of being a “high-confidence” miRNA is the alignment of mature miRNA/miRNA^*^ sequences with least 10 different reads of existing small RNA sequences (Kozomara and Griffiths-Jones, 2014). This stringent parameter is subservient in order to prove the genuineness of miRNA sequences identified from animals, particularly humans, however; this rule might be critical for plant miRNAs since the small RNA sequencing projects are relatively fewer compared to animals. Consequently, such situation may cause to overlook some important plant miRNAs which are experimentally well-characterized but cannot be detected among the high-confidence plant miRNAs because of small RNA alignment evidence. Consideration of defined problems, we constructed a list of high confidence miRNAs by prying out of high-confidence and experimentally-validated plant miRNAs from miRBase Release 21 which might be useful for future miRNA analyses in plants along with their identification. We believe that this miRNA list increase the sensitivity of our miRNA methodology for detection of genuine miRNA candidates in course of analysis.

The suggested methodology was tested with both genomic and transcriptomic data from *T. aestivum* and *B. distachyon*. These crops were particularly included in the analysis since both have an available genome sequence and different small RNA sequencing studies present in public databases. The outputs of “SUmirFind-Fold-Predictor” processes suggested that the number of identified miRNAs from transcriptomic data is much fewer compared to genome or both of the plants. Even though transcriptome assemblies may contain miRNA precursors which can be used for miRNA identification, generally they represented with a fairly small amount of transcriptome (~1% of both *B. distachyon* and *T. aestivum* transcriptomic data) since the miRNA precursors are prone to quick maturation into mature miRNA/miRNA^*^ duplexes, thus, such distribution of miRNA precursors in the transcriptomic data is expected (Kurihara and Watanabe, 2010). Remarkably, *T. aestivum* genome suggested much more miRNAs compared to *B. distachyon* (~7-fold more). Wheat is a hexaploid organism with a huge (17Gbp) and complex genome which consists of three different sub-genomes (A, B, and D) (Brenchley et al., 2012; Choulet et al., 2014); thus, the big difference between miRNA counts might be associated with the genome size differences.

Identified miRNAs from *T. aestivum* and *B. distachyon* were carefully named prior to the other down-stream analysis. Since some miRNA family members such as miR156/miR157 or miR159/miR319 show high similarities at the mature miRNA level (Voinnet, 2009; Jones-Rhoades, 2012), redundant miRNA annotations, meaning that exact same mature miRNA sequences with more than one miRNA ID in, may occur in the process of homology-based computational miRNA identification (Figure 3). The idea behind the addition of “SUmirPredictor” script to the pipeline was providing a more automatic and precise miRNA annotation, specifically considering the presence of redundant miRNA annotations and associated miRNA naming problems. In course of testing the pipeline with genomic and transcriptomic data, “SUmirPredictor” successfully detected and discarded redundant miRNA annotations where it also provide the name of miRNA sequences as separated by comma in case of equal similarity, which is a rare case (only three different pairs of miRNAs in identified from *Brachypodium* genome). This problem is mainly cause from ambiguous naming and mis-annotations of miRBase miRNAs (Budak et al., 2015a); consequently, the decision about the naming of such miRNA sequences is left to the user preference. Another problem arising from the mis-naming of miRBase miRNAs is the absence of hairpin arm information such as 3′ or 5′ arm of the related miRNA hairpin. The sequences of miRNAs which are coming from the different arms of the same hairpins are highly different from each other. The lack of this arm information in the miRNA naming cause the presence of many miRNA sequences which are associated with the same miRNA family but relatively different in the sequence level. This methodology also suggested a solution for this naming problem and it successfully named the newly identified miRNA sequences respect to both the hairpin information about the homology miRNA presented in reference miRNA set and the miRNA hairpin information coming as a result of “SUmirFold” process.

Following the naming process, putative miRNA sequences were inspected to detect any presence of contaminations which may come from any other type of small non-coding RNA sequences in the process of computational identification steps are also controlled. The existence of miRNA sequences derived from other non-coding sequences, tRNA sequences in humans has been shown in a few studies even though no evidence has been detected for plants yet (Schopman et al., 2010; Maute et al., 2013). Presence of such miRNAs might cause false-positive identification of miRNA sequences. In another case, the miRNA coding precursor might have dual coding capacities for both miRNAs and other type of small RNAs (Petfalski et al., 1998; Lee et al., 2009). Thus, elimination of small non-coding RNA sequences prior to the miRNA identification analysis may cause fails to notice such sequences. With the consideration of all these points, identified miRNA sequences and their precursors were aligned to other non-coding small RNA sequences via Blast algorithm and any such contamination was not detected across the whole identified miRNAs from both *Brachypodium* and *Triticum* which shows the sensitivity of our pipeline for specifically identifying miRNA sequences. Additionally, any present association of identified miRNA sequences with organellar genome was also checked by BLAST alignment of miRNA sequences to organellar genomes; however, no organelle-related miRNA was identified. The miRNA sequences originating from organellar genomes is an emerging topic, specifically in human miRNA research (Borralho et al., 2015; Srinivasan and Das, 2015). Up to now, no organelle genome associated miRNA sequence was detected in plants. Such situation may arise from a different regulation and biogenesis of organelle originating miRNA sequences; however, further research is necessary.

In order to check the genuineness of identified miRNAs, several other analyses were conducted in this methodology. The *in silico* expression analysis of identified putative miRNAs showed the presence of identified miRNAs both at the pre-miRNA and mature miRNA levels even though mining evidence for the precursor sequences from EST and transcriptome data can be less productive, compared to mature miRNAs. Since the processing of plant mature miRNAs from the precursor sequences quickly happens and their lifespan is very limited, the abundance of these precursors is relatively rare across EST and cDNA sequences (Kurihara and Watanabe, 2010; Budak and Akpinar, 2015). However, it is still possible to detect a few precursor sequences which serve as an evidence for the genuineness of the computationally identified miRNAs. Thus, the fewer detection of miRNA precursors in the *in silico* miRNA expression analysis is an expected result. In the small RNA expression analysis, both the mature miRNA and miRNA^*^ sequences were aligned to the reads of small RNA sequencing since the miRNA/miRNA^*^ is presented as duplex inside of the cell and they are separated from each other in the complementary target binding step (Naqvi et al., 2012; Rogers and Chen, 2013). As a result of *in silico* small RNA expression analysis, overall more than 40% of all identified mature miRNA/miRNA^*^ duplexes from *B. distachyon* and *T. aestivum* were aligned to the small RNA libraries with 100% query identity and coverage together with satisfying at least three reads cut-off which supports the genuineness of these identified miRNAs. The miRNAs which did not provide small RNA expression evidence with regard to our parameter might still be genuine miRNAs since the expression evidence for miRNA^*^ sequences is hard to obtain. The miRNA^*^ sequences are generally much less stable compared to mature miRNA sequences and the detected miRNA^*^ sequences in small RNA sequencing libraries are generally associated with highly expressed miRNAs (Finnegan and Pasquinelli, 2013). Additionally, the mature miRNA/miRNA^*^ duplexes which are not detected in the small RNA libraries might be still expressed under highly specific conditions. In another case, these miRNAs might be detected in the sequencing experiment when they bind to their targets; however, the experimental evidence is necessary in order to prove their existence under given conditions. In addition to *in silico* miRNA analysis, the reads from sRNAs were also mapped identified pre-miRNA sequences to show evidence for the genuineness of identified miRNA precursors. Alignment results showed that many pre-miRNAs were covered by small RNA reads on the location that “SUmirFold” detect the miRNA presence (Figure 7). The pre-miRNAs which were not covered with small RNA reads can still be genuine miRNA sequences since the small RNA libraries are highly condition/time/tissue specific. Particularly in consideration of miRNAs identified from genome, they might be expressed just under specific condition which may not be covered by the aligned sRNA libraries, thus it is an expected result that whole genome miRNAs are not covered by sRNA reads.

Along with *in silico* expression analysis, the genuineness of the identified miRNA sequences were also controlled by inspection of specific miRNA-miRNA precursor characteristics such as length of mature miRNA and miRNA precursors, MFE and MFEI values. In this analysis, the most of the mature miRNAs were detected as 21 nucleotide long which is an expected situation for plant miRNAs (Thakur et al., 2011). Identified mature miRNA hairpins showed low MFE value which is consistent with previous results from literature (Kurtoglu et al., 2014; Akpinar and Budak, 2016). Despite low MFE is a crucial indicator for the presence of miRNA-associated hairpins (Bonnet et al., 2004), it might be an unreliable source for plant miRNA mining process since miRNA precursors have significant variation in length (Thakur et al., 2011). Thus, high MFEI values were considered as significant point for discrimination process of miRNAs from other RNA species such as tRNAs (MFEI = 0.64), rRNAs (MFEI = 0.59), mRNAs (MFEI = 0.62–0.66) or pseudo-hairpins produced by coding sequences (Schwab et al., 2005; Kantar et al., 2012). Detected high MFEI values from this study also showed convenience with previous studies (Zhang et al., 2007; Jin et al., 2008; Kantar et al., 2012) and serve as a consistent point for the genuineness control of our miRNA identification method.

Understanding the genomic organization of miRNA genes together with their transcriptional regulation provide insights into their biogenesis (Guo et al., 2014). The genomic location of *MIR* genes may affect the generation and maturation of miRNAs. It was shown that multiple miRNAs sometimes come from the same transcript or from different alternative spliceoforms of the same gene (Olena and Patton, 2010; Nozawa et al., 2012). For such miRNAs, it is also possible to have a type of regulatory circuit which may generate fluctuations in the miRNA expression level under biotic and abiotic stresses (Rajwanshi et al., 2014; Dolata et al., 2016). In the light of such information pointing out the importance of miRNA location, “SUmirLocator” script was added on our pipeline and it successfully represented the genomic/transcriptomic distribution of identified putative miRNA families. Both in *Brachypodium* and *T. aestivum* genome, miRNA sequences from the same family were generally detected as located in a close proximity with each other. These sequence isoforms of same miRNA family members may target either the same or different target genes. It is also possible that the primary transcripts of such miRNA genes might be common and their regulation might be conducted with other regulator elements at the pre-miRNA level and mature miRNA level. The presence of many mature miRNA sequences transcribed from same pre-miRNA may represent some examples of such situations (Supplementary Document 5). Although the regulation of miRNA genes is well-studies and the presence of such miRNAs were shown in animals, there is not adequate information for plants (Cai et al., 2009; Slezak-Prochazka et al., 2010; Schanen and Li, 2011). “SUmirLocator” results provided a rough idea regarding to the genomic organization of miRNAs together with their regulation at the transcriptomic level. In addition, it is possible to detect the chromosomal and sub-genomal distribution of miRNA sequences. For instance, results of “SUmirLocator” process suggested that B sub-genome of *T. aestivum* contains more miRNA coding regions.

The outcomes of “SUmirLocator” process from the transcriptomic data further provided insights about biogenesis and transcriptional control of miRNA genes. Several miRNAs identified from *T. aestivum* transcriptomic data such as miR1120 and miR1436 were detected as generated from the same miRNA precursor. Both of these miRNA sequences has a potential to be transformed into genuine mature miRNA sequences by the effect of DICER-LIKE enzyme or in another case, one of these miRNAs might turn into functional mature miRNAs. Interestingly, the generation of different isoforms of same contig, which might have resulted from a possible alternative splicing event, did not affect the possible miRNA coding region of transcript in case of miR1120–miR1436 (Supplementary Document 5). However, it might also be possible that alternative splicing events on miRNA genes has an effect on the generation of different mature miRNA sequences. Although there is a lack of such studies for plants in the literature, research from other organisms shows the possibility of such events (Rasschaert et al., 2016). “SUmirLocator” results also show the importance of transcriptional direction for the miRNA generation process. In the case of miR1128 and miR1436 (contig c199396_g2_i3, Table 3), the sense and antisense transcription of exact same miRNA gene resulted in the generation of different miRNAs. Regarding to associate transcriptional signal, which comes from upstream regulatory processes, the choice of transcriptional direction might be done, consequently the choice of miRNA. Thus, we suggest that “SUmirLocator” script might be useful in the further search of such transcriptional regulation of miRNA genes.

“SUmirLocator” also offer important information about possible genomic copy number and expression profiles of identified putative miRNA families under given condition based on their genome/transcriptome-wide representation. In our analysis, some miRNAs such as miR1436 and miR1439 from *Brachypodium* and miR1122 from *Triticum* have been represented with more genome/transcriptome-wide copy number compared to others. Such miRNA families were remarked as “highly-representative” which tends to obtain robustness at both mature and precursor miRNA levels. The high representation of miRNA families may be associated with several other factors. First of all, TE-associated miRNA families might show high expression compared to others since repetitive elements are ubiquitously found all around the genome, specifically in crops like wheat. Also, highly-represented miRNAs may target specific mRNAs which have essential functions in the regulation of cellular life. As an example, many targets of miR1436 family members were associated with important enzymes such as “serine-threonine protein kinases” involved in signal transduction pathways. The post-transcriptional regulation of signaling elements via miRNAs has already been shown with a few animal studies (Inui et al., 2010; Zhao et al., 2013) and the robust expression of this miRNA might be associated with its regulatory function in signaling pathway. On the other hand, the miRNA families targeting the molecules which have many isoforms also be expressed highly compared to others, which also might be the case for miR1436. However, in both cases, experimental validation of the miRNA-target pairs is necessary and “SUmirLocator” may provide a more focused experimental design for such experiments.

In many organisms, TE–miRNA association has been fairly represented; hence identification of miRNAs from overall sequences including TEs is crucial with the aim of unlocking the complete set of miRNAs in a given species (Yao et al., 2007; Piriyapongsa and Jordan, 2008; Li et al., 2011; Gim et al., 2014; Budak and Kantar, 2015). Common practice in miRNA identification both in plants and animals usually involves elimination of these repetitive sequences prior to miRNA identification, in order to avoid mis-annotation of repeat-related siRNAs as miRNAs; however, this approach overlooks genuine miRNAs encoded by TEs (Li et al., 2011; Budak and Akpinar, 2015). In order to avoid inadequate detection of miRNAs, our miRNA identification guideline does not include repeat-masking step prior to miRNA mining from genomic/transcriptomic data; instead, it analyzes identified putative pre-miRNA structures with respect to their relation with TEs. In our analysis, majority of the identified miRNAs was associated with TEs, particularly DNA transposons. There are a few hypotheses which attempt to describe the relation of miRNAs and TEs. One of the miRNA evolution hypothesis claims that miRNAs are evolved as a result of TE-to-MITE (Miniature Inverted Repeat Transposable Elements) transition (Piriyapongsa and Jordan, 2008; Fattash et al., 2013). In addition to TE-to-MITE transition, current studies propose that miRNA genes are generated with accumulation of mutations in inverted repeat sequences, while some other hypothesis suggesting direct transcription of miRNAs from TEs with the assistance of transcriptionally regulative elements (Fahlgren et al., 2007; Feldman and Levy, 2012; Roberts et al., 2014). According to high content of repetitive elements associated with pre-miRNA sequences, our results also agree with previous studies and highlight the importance of identification of TE associated miRNAs. Additionally, an important proportion of identified TE-miRs showed *in silico* expression evidence at the mature miRNA level which may indicate their genuineness. In order to obtain a detailed observation about TE-miRs, experimental validation of concerned ones might be necessary. However, it must be underlined that the elimination of repetitive sequences in course of miRNA analysis stands as a highly speculative option.

Another puzzling issue about TE-miRs is the discrimination siRNAs across TE-miR pools. siRNA molecules have many common characteristics with miRNAs and this situation sometimes result with false-positive prediction of TE-miRNAs and siRNAs (Tang, 2005; Lucas and Budak, 2012). siRNA molecules are evolved to suppress the activity of transposable elements in plant genomes; thus, they show a high degree of complementarity to TE elements (Ito, 2012; Thiebaut et al., 2014). Considering this, we grouped the miRNAs which show a resemblance to TE elements as siRNA candidates and TE-miRs, where the miRNAs which their precursors aligned to TE elements with a perfect or almost-perfect complementary manner. The amount of siRNA molecules were generally detected as “low” except the miRNAs identified from *T. aestivum* genome. Across all the miRNAs associated with TE elements, a significant amount of siRNA candidates (2712 sequences, Supplementary Document 7) were also detected as showing *in silico* miRNA expression at mature miRNA level which means that they were aligned to small RNA sequence together with their star sequences. Thus, it is not certain that these sequences are “actual siRNA” and further experimental evidences are necessary to show whether this sequences are TE-miRs or siRNAs. Additionally, miR1117 which shows evidences for being siRNA candidate did not represent any CDS target in the target analysis process which might suggest that its original target can be TEs where this sequence must be annotated as siRNA, in such case. Additionally, the association of this miRNA with TE element and chromosome-wide high representation was previously shown in wheat (Lucas and Budak, 2012). In order to understand the genuineness of this miRNA, further experimental characterization remains necessary.

Accurate and precise identification of microRNAs is the key step for the miRNA research both in animals and plants. Despite the presence of many comprehensive and reliable miRNA detection methods in animals, accurate identification of plant miRNAs still stands as a problematic issue. Here, we presented a comprehensive methodology for plant miRNA identification and its further computational characterization. Our method relies on homology-based and comparative prediction of miRNAs in a given genomic or transcriptomic sequence and it has ability to predict miRNA sequences in a sensitive manner with their further detailed characterization across different plants. Currently, the most popular miRNA identification method is small RNA sequencing which is an expensive and highly condition-specific tool. Identification of miRNA sequences from DNA-RNA sequences provides an overview of the potential miRNA repertoire of the plant species which might not be represented by small RNA sequencing studies since the miRNAs obtained from small RNA reads are specifically expressed miRNAs in a given condition. Considering this, we designed our methodology as optimized for genome and transcriptome-wide miRNA mining. Independently of this optimization, we also showed that this pipeline can be used with small RNA sequencing data, with minor modifications. Consequently, we were able to identified miRNA sequences from genomic/transcriptomic and small RNA sequencing data.

Despite the presence of several miRNA identification pipelines present in literature, there is a lack of comprehensive guideline for characterization of plant miRNAs. We believe that our methodology suggests insightful solutions to cover this absence by providing a detailed analysis of identified miRNAs. It provides a miRNA naming strategy which takes into consideration of redundant miRNA annotation problem together with the miRNA hairpin characterization. It suggests a methodology for inspection of miRNA & TE association both in the manner of TE-miR and siRNA candidates' identification. It also provides detailed information about miRNA localization together with clues about possible effects of alternative splicing events in *MIR* genes. Additionally, it suggests a solution to miRNA-target pair enrichment problem where the precise elimination of false-positives targets can conduct. Furthermore, it offers an *in silico* expression analysis for both at pre-miRNA and mature miRNA level. Our pipeline, with further refinements presented in this study, has already provided efficient results for complex crop species and it can be utilized for all of the other genomic/transcriptomic data associated with diploid or polyploid plant species. Additionally, the high-confidence miRNA list released in this study can be used as a reference guide for several miRNA analyses.

## Author contributions

HB conceived and designed the study, supervised all analyses and prepared the final manuscript. BA performed the computational analyses and drafted the manuscript. BAA contributed to the computational analyses and revised the draft manuscript.

## Funding

This research was funded by Montana Plant Sciences Endowment.

### Conflict of interest statement

The authors declare that the research was conducted in the absence of any commercial or financial relationships that could be construed as a potential conflict of interest.
