# Supplementary material for: A Comprehensive Prescription for Plant miRNA Identification
Source: Front Plant Sci. 2017 Jan 24;7:2058. doi: 10.3389/fpls.2016.02058 (PMC5258749; doi:10.3389/fpls.2016.02058)
Supplement: Supplementary file 1 [file Table1.pdf]

Supplementary Document 1: ‘SUmirPredictor’ and ‘SUmirLocator’ Perl scripts

---

SUmirPredictor.pl: A script that uses SUmirFold.pl outputs to screen structures of predicted miRNAs for specified folding criteria by conservation Copyright © Hikmet Budak, 2016. Digital copies of the scripts and a usage guide are freely available from the author (e-mail: hikmet.budak@montana.edu)

Folding criteria:

1- Mismatches: max 4 for sense and max 6 for antisense strands

2- Dicer: mismatched sequences in the cut points of mature miRNA and miRNA* start sites

3- Multiloop: only one loop is acceptable between mature mirna and mirna*

4- Head: both mature mirna and mirna* sequences should not be involved in the head part)

```
use strict;

use warnings;

my $filename = "";

my $dic = "";


##################################################

my $usage = "SUmirPredictor.pl SUmirFold.hairpins.tbl.fileoutput SUmirFold.hairpins.folderoutput";

##################################################


if ($#ARGV==-1){

    print("Correct usage is: ".$usage."\n");

    exit -1;

} else {

    if ($#ARGV==1){

        $filename = $ARGV[0];
```

```
        $dic = $ARGV[1];
    } else {
        $filename = $ARGV[0];
        $dic = substr($filename, 0, length($filename)-4);
    }
}
if (!-d $dic) {
    print("Correct usage is: ".$usage."\n");
    exit -1;
}

my @hairpins = ();
my @start1 = ();
my @end1 = ();
my @screen = ();
my @whole_data = ();
my @blacklist = ('Unique', 'Hit', 'Hit ID');

open(my $FILE, $filename) || die "Cannot open file: ".$filename."\n";
chomp(my @lines = <$FILE>);
close $FILE;

my $x = -1;
my $lineID = 0;
```

```
for(my $i=0; $i<=$#lines; $i++){
    $lineID++;
    my $line = $lines[$i];
    my @lineelems = split(/\t/, $line);

    if ($line ne "" && substr($line, 0, 2) ne "\t" && !(grep( /^$lineelems[0]$/, @blacklist ))){
        $x++;
        my $n = scalar(@lineelems);
        my $temp = $line;

        if ($n < 20){
            $temp .= $lines[$lineID+1];
            my @tmparr = split(/\t/, $lines[$lineID+1]);
            $n += scalar(@tmparr);
            if ($n < 20){
                $temp .= $lines[$lineID+2];
            }
        }

        my @store = split(/\t/, $temp);
        push(@whole_data, $temp);
        push(@hairpins, $dic."/".$store[0].".hairpin.fsa_1.ct");
        push(@start1, $store[8]);
        push(@end1, $store[9]);
```

```
        push(@screen, '');
    }
}

my $er2count = 0;
my $count = -1;
my @b = ();
foreach my $i (@hairpins){
    $count++;

    open(my $FILE2, $i) || die "Cannot open file: ".$i."\n";
    chomp(my @lines2 = <$FILE2>);
    close $FILE2;

    my @a = (0);
    @b = (0);
    my @c = ('0');
    my $m = 0;

    eval {
        foreach my $line (@lines2) {
            $m++;
            if ($m > 1 && $line ne "") {
                my @store = split(/\t/, $line);
```

```
        push(@a, $store[0]);
        push(@b, $store[4]);
        push(@c, $store[1]);
    } elsif ($m == 1) {
        my $primirnalength = (split(/\t/, $line))[0];
    }
}

# 1- check for mismatches, max 4 for sense and max 6 for antisense
my $mismatchB = 0;
my $start2 = $b[$end1[$count] - 2];
my $end2 = $b[$start1[$count]] + 2;
my $mirna2 = '';

for (my $j = $start2; $j < $end2 + 1; $j++) {
    $mirna2 .= $c[$j];
    if ($b[$j] == 0) {
        $mismatchB++;

        if ($mismatchB > 6) {
            $screen[$count] = "MismatchB";
        }
    }
}
```

```
my @newdata = split(/\t/, $whole_data[$count]);
$newdata[10] = $start2;
$newdata[11] = $end2;
$newdata[12] = $mirna2;

$whole_data[$count] = join("\t", @newdata);
if (!$screen[$count]) {
    my $mismatchA = 0;
    for (my $j = $start1[$count]; $j < $end1[$count] + 1; $j++) {
        if ($b[$j] == 0) {
            $mismatchA++;

            if ($mismatchA > 4) {
                $screen[$count] = "Mismatch";
            }
        }
    }
}


# 2- check for Dicer functions
if (!$screen[$count]) {
    if ($b[$start1[$count]] == 0) {
```

```
        $screen[$count] = "Dicer";
    }
    if ($b[$end1[$count] - 2] == 0) {
        $screen[$count] = "Dicer";
    }
}

# determine start-end sites
my $start = $start1[$count];
my $end = $end2;

if ($start2 < $start) {
    $start = $start2;
    $end = $end1[$count];
}

# 3- check for multiloop
if (!$screen[$count]) {
    my $temp = $b[$start];
    for (my $k = $start + 1; $k < $end + 1; $k++) {
        my $check = $b[$k];
        if ($check != 0) {
            if ($check < $temp) {
                $temp = $check;
```

```
            } elsif ($check > $temp) {
                $screen[$count] = "Multiloop";


            }
        }
    }
}



# 4- check for head
if (!$screen[$count]) {
    my $temp = $b[$start];
    my $check = 0;
    for (my $k = $start + 1; $k < $end + 1; $k++) {
        if ($b[$k] != 0) {
            if ($check == $b[$k]) {
                my @u2 = ();
                for (my $u = $k - $x; $u < $k; $u++) {
                    push(@u2, $u);
                }

                for (my $test = $start1[$count]; $test < $end1[$count] + 1; $test++) {
                    if (grep(/^$test$/, @u2)) {
                        $screen[$count] = "Head";
```

```
                    }
                }
                if (!$screen[$count]) {
                    for (my $test = $start2; $test < $end2 + 1; $test++) {
                        if (grep(/^$test$/, @u2)) {
                            $screen[$count] = "Head";
                        }
                    }
                }
            }
            $x = 0;
        } elsif ($b[$k] == 0) {
            $x += 1;
            $check = $a[$k - 1] if ($x == 1);
        }
    }
}
if (!$screen[$count]) {
    $screen[$count] = "OK";
}
}; if($@) {
    $er2count++;
}
}
```

```
open(my $OUTPUT, ">".$filename.".edited.tbl") || die "Cannot open file for writing: ".$filename.".edited.tbl\n";

print $OUTPUT "Screen\tUnique Hit ID\tNew miRNA ID\tNew miRNA Sequence\tNew miRNA Length\tConserved miRNA ID\tConserved miRNA Sequence\tConserved miRNA Mismatch\tSequence ID\tMature Start\tMature End\tmiRNA* Start\tmiRNA* End\tmiRNA* Sequence\tHairpin Location\tPre-miRNA Length\tPre-miRNA MFE\tPre-miRNA GC%\tPre-miRNA MFEI\tPre-miRNA Start\tPre-miRNA Sequence\tmirna* Sequence";



my @mirnas = ();

my @seqs = ();

my @mismatch = ();

my @homolog = ();

my @index = ();

my @location = ();

my @mirna2 = ();



for (my $i=0; $i < scalar(@screen); $i++){

    my $data = $whole_data[$i];

    my @splitdata = split(/\t/, $data);

    print $OUTPUT "\n".$screen[$i]."\t".$data;

    my $start2 = $b[$end1[$count]-2];

    my $end2 = $b[$start1[$count]] + 2;



    if ($screen[$i] eq "OK" && (index("N", $splitdata[2]) == -1)){

        eval {

            push(@seqs, $splitdata[2]."\t".$splitdata[19]);
```

```
            my $mirID = (split(/\./, (split(/-/, $splitdata[1]))[1]))[0];
            my $mir = $mirID;
            $mir =~ s/[^\d]//g;
            push(@mismatch, $splitdata[6]);
            push(@homolog, $splitdata[4]);
            push(@index, $i+1);
            my $loc = substr($splitdata[13], 0, 1);
                                push(@mirna2, $splitdata[12]);
            push(@location, $loc);
            my $homolog_loc = (split(/-/, $splitdata[4]))[-1];
            if (grep(/^$homolog_loc$/, ("3p", "5p")) && $loc ne substr($homolog_loc, 0, 1)){
                $mir = $mir;
            } else {
                $mir .= "-".$loc."p";
            }
            push(@mirnas, "miR".$mir);
        } or do {
            print("3: error in: ".$data."\n");
        };
    }
}

my %full;
for (my $i = 0; $i < scalar(@mirnas); $i++){
```

```
if (grep(/^$seqs[$i]$/, keys(%full))){
                    my $mirnas_now = $mirnas[$i];
    if ($full{$seqs[$i]}[1] > $mismatch[$i]){
        $full{$seqs[$i]} = [$mirnas_now, $mismatch[$i], $index[$i], $homolog[$i], $mirna2[$i]];
    } elsif($full{$seqs[$i]}[1] == $mismatch[$i]) {
        if (! grep(/^$mirnas[$i]$/, split(/,/, $full{$seqs[$i]}[0]))) {
            $full{$seqs[$i]}[0] .= ','.$mirnas[$i];
            $full{$seqs[$i]}[2] .= ','.$index[$i];
                                        if (! grep(/^$homolog[$i]$/, split(/,/, $full{$seqs[$i]}[3]))){
                                                    $full{$seqs[$i]}[3] .= ','.$homolog[$i];
                                                    $full{$seqs[$i]}[4] .= ','.$mirna2[$i];
                                        }
            print("similar mismatch found. look at mirnaID: ".$full{$seqs[$i]}[0]."\n");
        } elsif(grep(/^$mirnas[$i]$/, split(/,/, $full{$seqs[$i]}[0]))) {
                                        $full{$seqs[$i]}[2] .= ','.$index[$i];
                                        if (! grep(/^$homolog[$i]$/, split(/,/, $full{$seqs[$i]}[3]))){
                                                    $full{$seqs[$i]}[3] .= ','.$homolog[$i];
                                                    $full{$seqs[$i]}[4] .= ','.$mirna2[$i]
                                        }
                              }
    }
} else {
    $full{$seqs[$i]} = [$mirnas[$i], $mismatch[$i], $index[$i], $homolog[$i], $mirna2[$i]];
}
```

```
}
close($OUTPUT);


open(my $OUTPUT2, ">".$filename.".out.tbl");
foreach my $x (keys(%full)){
    my @tmparr = @{$full{$x}};
    my $joinedstr = join("\t", splice(@tmparr, 2, scalar($full{$x})-2));
    print $OUTPUT2 $full{$x}[0]."\t".$x."\t".$joinedstr."\n";
}
close($OUTPUT2);


print("Number of structures with missing information: ".$er2count."\n");
print("\nFinished successfully.\n");
```

SUmirLocator.pl –Perl script that uses SUmirPredictor.pl outputs to count the number of occurrences of putative miRNAs in a given genome (or any other fasta file) by conservation. Copyright © Hikmet Budak, 2016. Digital copies of the scripts and a usage guide are freely available from the author (e-mail: hikmet.budak@montana.edu)

```
#!/usr/bin/env perl
# mirna representation (IDs are in forms of miR156 etc.)
##################################################
use strict;
use warnings;
##################################################
my $usage = "SUmirLocator.pl genome.fasta SUmirPredictor.output.out.tbl";
```

```
################################################
my $genome;
my $filename;
if ($#ARGV<1){
    print("Correct usage is: ".$usage."\n");
    exit -1;
} else {
    $genome = $ARGV[0];
    $filename = $ARGV[1];
}

# --------------------


sub alllocations {
    my ($alist, $x) = @_;

    my @ret = ();
    while ($alist =~ /($x)/g) {
        push @ret, (pos($alist)-length $1);
    }

    return @ret;
}
```

```
# ----------------------

open (my $FILE, $genome) or die "Cannot open genome file: ".$genome."\n";
my @geneID = ();
my @geneSeq = ();
my $done = 0;
my $seq = '';

while (my $line = <$FILE>) {
    chomp($line);
    if (substr($line, 0, 1) eq ">") {
        if ($seq ne '') {
            push(@geneSeq, $seq);
            $seq = '';
        }
        push(@geneID, $line);
        $done = 1;
    } else {
        if ($done == 1) {
            $seq .= $line;
        }
    }
}
push(@geneSeq, $seq);
```

```
close($FILE);


# --------------------


# my $filename = "plantmiRNAs.txt.fsa.results.tbl.hairpins.tbl.out.tbl";

open (my $FILE2, $filename);

chomp(my @alllines = <$FILE2>);

open (my $OUT1, ">".$filename.".edited");

open (my $OUT3, ">".$filename.".expression.tbl");


my %senseSeq = ();

my %antiSeq = ();

my @mirnaID = ();

my %mirnaIndex = ();

my %howmany = ();


for(my $i = 0; $i <= $#alllines; $i++){

    my $line = $alllines[$i];

    if (substr($line, 0, 3) eq "miR"){

        my @lineelems = split(/\s/, $line);

        my $mirna = $lineelems[0];

        my $mirna2 = $lineelems[-1];


        if (grep(/^$mirna$/, @mirnaID)) {
```

```
    my $a = $mirnaIndex{$mirna}+1;
    $mirnaIndex{$mirna} = $a;
} else {
    $mirnaIndex{$mirna} = '1';
    push(@mirnaID, $mirna);
}

my $index = $lineelems[3];


my $seq = $lineelems[2];
my $newseq = $seq;
$newseq =~ tr/UAGC/ATCG/;
my $newseq2 = $seq;
$newseq2 =~ tr/UAGC/TAGC/;

while($seq =~ m/([^UAGC])/g){
    print("something is wrong with ".$seq."\n")
}
$newseq = reverse($newseq);

if (grep(/^$newseq2$/, keys %senseSeq)) {
    $senseSeq{$newseq2} .= "\t".$mirna.'-'.$mirnaIndex{$mirna}.','.$index;
} else {
    $senseSeq{$newseq2} = $mirna."-".$mirnaIndex{$mirna}.",".$index;
```

```
    }

    if (grep(/^$newseq$/, keys %antiSeq)) {
        $antiSeq{$newseq} .= "\t".$mirna.'-'.$mirnaIndex{$mirna}.','.$index;
    } else {
        $antiSeq{$newseq} = $mirna."-".$mirnaIndex{$mirna}.",".$index;
    }

    my $summary = join("\t", @lineelems[1..$#lineelems]);

    foreach my $mir2 (split(/,/, $mirna2)){
        print $OUT3 $mirna.'-'.$mirnaIndex{$mirna}."\t".$lineelems[1]."\t".$mir2."\n";
    }

    print $OUT1 $mirna."-".$mirnaIndex{$mirna}."\t".$summary."\n";

    #   If you want to count mirna Isomers instead of general mirnaIDs
    #    uncomment the 1st line and comment the 2nd line below
    #my $newmirna = $mirna.'-'.$mirnaIndex{$mirna};
    my $newmirna = $mirna;
    if (!(grep(/^$newmirna$/, keys %howmany))){
        $howmany{$newmirna} = [0, 0];
    }
}
```

```
}
close($FILE2);
close($OUT1);
close($OUT3);
# --------------------------


open(my $OUT2, ">premirna-locations.csv") or die "Cannot create file: premirna-locations.csv\n";
print $OUT2 "mirnaID\tindex\treadID\tpremirna location\tpremirna length\tstrand";
eval{
    foreach my $seq2 (keys %senseSeq){
        my $geneCount = -1;
        for(my $j = 0; $j <= $#geneSeq; $j++){
            my $gene = $geneSeq[$j];
            $geneCount++;
            my @loc1 = alllocations($gene, $seq2);
            if (scalar(@loc1) > 0) {
                foreach my $loc (@loc1) {
                    my @allmirnas = split(/\t/, $senseSeq{$seq2});
                    foreach my $mirnas (@allmirnas) {
                        my @mirnaselems = split(/,/, $mirnas);
                        my @firstelems = split(/-/, $mirnaselems[0]);
                        my @geneIDelems = split(/\s/, $geneID[$geneCount]);
                        my $mirna = join("-", @firstelems[0..($#firstelems-1)]);
                        my $index = $mirnaselems[1];
```

```
                        print       $OUT2       "\n".$mirnaselems[0]."\t".$index."\t".substr($geneIDelems[0],       1,
length($geneIDelems[0]))."\t".$loc."\t".length($seq2)."\tSENSE";


                        if (!grep(/^$mirna$/, (keys %howmany))){
                            die;
                        }
                        $howmany{$mirna}[0]++;
                    }
                }
            }
        }
    }
    foreach my $seq3 (keys %antiSeq){
        my $geneCount = -1;
        foreach my $gene (@geneSeq){
            $geneCount++;
            my @loc1 = alllocations($gene, $seq3);
            if (scalar(@loc1) > 0) {
                foreach my $loc (@loc1) {
                    my @allmirnas = split(/\t/, $antiSeq{$seq3});
                    foreach my $mirnas (@allmirnas) {
                        my @mirnaselems = split(/,/, $mirnas);
                        my @firstelems = split(/-/, $mirnaselems[0]);
                        my @geneIDelems = split(/\s/, $geneID[$geneCount]);
                        my $mirna = join("-", @firstelems[0..($#firstelems-1)]);
```

```
                        my $index = $mirnaselems[1];

                        print $OUT2 "\n".$mirnaselems[0]."\t".$index."\t".substr($geneIDelems[0], 1, length($geneIDelems[0]))."\t".$loc."\t".length($seq3)."\tANTISENSE";

                        if (!grep(/^$mirna$/, (keys %howmany))){

                            die $mirnas;

                        }

                        $howmany{$mirna}[1]++;

                    }

                }

            }

        }

    }

}; if ($@) {

    print "Check if there is only one miRNA per line.\nYou should choose one from the miRNAs separated by commas.\nError in mirnaID ".$@."\n";

}

close($OUT2);



# ---------------------------------------



open(my $OUTPUT3, ">premirna-counts.csv") or die "Cannot create file:premirna-counts.csv\n";;

print $OUTPUT3 "mirnaID\ton SENSE\ton ANTISENSE\ttotal";

foreach my $many (keys %howmany){
```

```
print $OUTPUT3 "\n".$many."\t".$howmany{$many}[0]."\t".$howmany{$many}[1]."\t".($howmany{$many}[0]+$howmany{$many}[1]);

}

close($OUTPUT3);
```

SUmirFind_sRNA.pl– Perl script used for detection of small-RNA reads which align to known miRNA sequences with three of fewer mismatches. Copyright © Hikmet Budak, 2016. Digital copies of the scripts and a usage guide are freely available from the author (e-mail: hikmet.budak@montana.edu)

```
#!perl -w

print "\n\n+++ SUmirFind.pl v1.1 +++\n\n";



my ( $mirnaquery, $blastdatabase ) = @ARGV or die "Please specify a fasta file containing the miRNA sequences you wish to search with, and the BLAST database you wish to search, with full path if it is not in the current directory";



# Get number of mismatches from user



print "Please specify the maximum number of base mismatches you wish to allow between your query miRNAs and test sequences.\n";

print "Maximum permitted mismatches (1-3 recommended): ";

my $mislimit = <STDIN>;

chomp $mislimit;

$mislimit =~ s/\r//;

if ( $mislimit =~ m/\D+/ )

{

die "Your entry was not a number.  Please run again and enter digits only.\n";

}
```

```
else
{
print "Searching for miRNA sequences with a maximum of $mislimit mismatches.\n";
}

# Convert fasta file into a table of miRNAs

my $mirnacount = 0;
open ( MIRNAS, $mirnaquery ) or die "Could not open $mirnaquery.  Is it in the right folder?";
open ( MIRNATABLE, ">".$mirnaquery.".tbl" ) or die "Could not open an output file!";

print MIRNATABLE "# miRNA ID\tsequence";

while($line = <MIRNAS>)
{
	chomp $line;
	$line =~ s/\r//;
	if ($line =~ /^>(\S+)\s*/)
	{
		print MIRNATABLE "\n$1";
		print MIRNATABLE "\t";
		$mirnacount++;
	}
	else
```

```
		{
			print MIRNATABLE "$line";
		}
}
print MIRNATABLE "\n";

close MIRNAS;
close MIRNATABLE;
print "$mirnacount miRNA sequences detected in query file.\n";

# Populate hash table of miRNAs from newly generated table

my (%QuerymiRNAs);
open ( MIRNADATA, $mirnaquery.".tbl" ) or die "The miRNA table was not generated";

while ($line = <MIRNADATA>)
{
		chomp $line;
		if ( $line =~ /^#/ )
		{
			next;
		}
		elsif ( $line =~ /(\S+)\t(\S+)/ )
		{
```

```
                    my $idkey = $1;

                    my $seqval = $2;

                    $QuerymiRNAs{"$idkey"} = "$seqval";

          }

}



close MIRNADATA;

my $mirnacount2 = scalar(keys(%QuerymiRNAs));



print "miRNA data analysed....  Now running BLAST for $mirnacount2 miRNAs.  This could take some time.\n";



# Run BLAST for the specified miRNAs



system ("blastn -task blastn-short -query $mirnaquery -db $blastdatabase -qcov_hsp_perc 100 -ungapped -penalty -1 -reward 1 -strand plus -dust 'no' -evalue 1000 -word_size 7 -outfmt 6 -out $mirnaquery.allhits" );



# Filter blast hit table (in output format 6) to remove alignments with >specified mismatches



print "BLAST complete, now filtering.\n";



open ( BLASTHITS, $mirnaquery.".allhits" ) or die "Couldn't find the BLAST output!";

open ( FILTEREDHITS, ">".$mirnaquery.".results.tbl" ) or die "Results file failure";
```

```
print FILTEREDHITS "# Query ID\tSubject ID\t%\tlength\tmism\tgaps\tqstart\tqend\tsstart\tsend\tevalue\tbitscore\n";

my $badcount = 0;

my $goodcount = 0;

while ( $line = <BLASTHITS> )

{

	chomp $line;

	my ( $qid, $sid, $percent, $allength, $mismatch, $gaps, $qstart, $qend, $sstart, $send, $evalue, $bitscore ) = split /\t/, $line;

	my $qlength = length $QuerymiRNAs{ $qid };

	my $difference = $qlength - $allength;

	if ( $mismatch + $difference > $mislimit )

	{

		$badcount++;

		next;

	}

	else

	{

		print FILTEREDHITS "$line\n";

		$goodcount++;

	}

}

close BLASTHITS;

close FILTEREDHITS;
```

```
unlink ($mirnaquery.".tbl");

unlink ($mirnaquery.".allhits");



print "Filtering complete.  $badcount hits were rejected due to being too short, or having too many mismatches.\n";

print "$goodcount hits were recorded in the file ".$mirnaquery.".results.tbl\n";
```
