# Supplementary material for: A Comprehensive Prescription for Plant miRNA Identification
Source: Front Plant Sci. 2017 Jan 24;7:2058. doi: 10.3389/fpls.2016.02058 (PMC5258749; doi:10.3389/fpls.2016.02058)
Supplement: Supplementary file 2 [file Table2.PDF]

Supplementary Document 2 (fasta): The non-redundant list reference miRNAs used in this study which contains 1,404 different mature miRNA sequences. The list was constructed by utilization of miRBase database release 21 (http://www.mirbase.org/). Only ‘high confidence plant miRNA’ presented in miRBase (Kozomara & Griffiths-Jones, 2014) and miRNAs which have the experimental evidence (cloned by PCR, Q-RT PCR and Nothern Blot) were added to the list. All other miRNAs presented in miRBase and identified by ‘similarity’, ‘small RNA sequencing and ‘in silico methods’ were discarded.

>aly-miR156a-3p MIMAT0017398

GCUCACUGCUCUUUCUGUCAGA

>aly-miR156b-3p MIMAT0017400

GCUCACCUCUCUUUCUGUCAGU

>aly-miR156c-3p MIMAT0017402

GCUCACUGCUCUAUCUGUCAGA

>aly-miR156d-3p MIMAT0017404

GCUCACUCUCUUUCUGUCAUA

>aly-miR157c-3p MIMAT0017418

GCUCUCUAUACUUCUGUCACC

>aly-miR157c-5p MIMAT0017417

UUGACAGAAGAUAGAGAGCAC

>aly-miR158a-3p MIMAT0017422

UCCCAAAUGUAGACAAAGCA

>aly-miR158a-5p MIMAT0017421

CUUUGUCUACAAUUUUGGAAA

>aly-miR159a-3p MIMAT0017424

UUUGGAUUGAAGGGAGCUCUA

>aly-miR159a-5p MIMAT0017423

GAGCUCCUUGAAGUUCAAACG

>aly-miR159b-3p MIMAT0017426

UUUGGAUUGAAGGGAGCUCUU

>aly-miR159b-5p MIMAT0017425

GAGCUCCUUGAAGUUCAAUGG

>aly-miR160a-3p MIMAT0017436

GCGUAUGAGGAGCCAUGCAUA

>aly-miR167a-3p MIMAT0017474

GAUCAUGUUCGCAGUUUCACC

>aly-miR168a-3p MIMAT0017482

CCCGCCUUGCAUCAACUGAAU

>aly-miR168a-5p MIMAT0017481

UCGCUUGGUGCAGGUCGGGAA

>aly-miR169a-3p MIMAT0017486

GGCAAGUUGUCCUUGGCUACA

>aly-miR169a-5p MIMAT0017485

CAGCCAAGGAUGACUUGCCGA

>aly-miR169d-3p MIMAT0017492

GCAAGUUGACCUUGGCUCUGU

>aly-miR169e-3p MIMAT0017494

GCAAGUUGACCUUGGCUCUGU

>aly-miR169e-5p MIMAT0017493

UGAGCCAAGGAUGACUUGCCG

>aly-miR169g-3p MIMAT0017498

GCAAGUUGACCUUGGCUCUGU

>aly-miR169g-5p MIMAT0017497

UGAGCCAAGGAUGACUUGCCG

>aly-miR171b-3p MIMAT0017518

UUGAGCCGUGCCAAUAUCACG

>aly-miR171b-5p MIMAT0017517

AGAUAUUAGUGCGGUUCAAUC

>aly-miR171c-3p MIMAT0017520

UUGAGCCGUGCCAAUAUCACG

>aly-miR171c-5p MIMAT0017519

AGAUAUUGGUGCGGUUCAAUC

>aly-miR172a-5p MIMAT0017521

GUGGCAUCAUCAAGAUUCACA

>aly-miR172b-5p MIMAT0017523

GCAGCACCAUCAAGAUUCACA

>aly-miR173a-3p MIMAT0017532

GAUUCUCUGUGCAAGUGGAAG

>aly-miR173a-5p MIMAT0017531

UUCGCUUGCAGAGAGAAAUCAC

>aly-miR173b-3p MIMAT0031389

GAUUCUCUGUGCAAGUGGAAG

>aly-miR173b-5p MIMAT0031388

UUCGCUUGCAGAGAGAAAUCAC

>aly-miR391-3p MIMAT0017538

ACGGUAUCUCUCCUACGUAGC

>aly-miR391-5p MIMAT0017537

UUCGCAGGAGAGAUAGCGCCA

>aly-miR396a-3p MIMAT0017558

GUUCAAUAAAGCUGUGGGAAG

>aly-miR396b-3p MIMAT0017560

GCUCAAGAAAGCUGUGGGAAA

>aly-miR403a-3p MIMAT0017586

UUAGAUUCACGCACAAACUCG

>aly-miR403a-5p MIMAT0017585

UGUUUUGUGCGUGACUCUAAUU

>aly-miR403b-5p MIMAT0031386

UGUUUUGUGCGUGACUCUAAUU

>aly-miR824-3p MIMAT0017604

CCUUCUCAUCGAUGGUCUAGA

>aly-miR824-5p MIMAT0017603

UAGACCAUUUGUGAGAAGGGA

>aly-miR862-3p MIMAT0017654

ACAUGCUGGAUCUACUUGAAG

>aly-miR862-5p MIMAT0017653

UCCAAUAGGUCUAGCAUGUGC

>ath-miR156b-3p MIMAT0031866

UGCUCACCUCUCUUUCUGUCAGU

>ath-miR156d-3p MIMAT0031868

GCUCACUCUCUUUUUGUCAUAAC

>ath-miR156e MIMAT0000170

UGACAGAAGAGAGUGAGCAC

>ath-miR156f-3p MIMAT0031869

GCUCACUCUCUAUCCGUCACC

>ath-miR156h MIMAT0001013

UGACAGAAGAAAGAGAGCAC

>ath-miR157a-3p MIMAT0031870

GCUCUCUAGCCUUCUGUCAUC

>ath-miR157a-5p MIMAT0000172

UUGACAGAAGAUAGAGAGCAC

>ath-miR157b-3p MIMAT0031871

GCUCUCUAGCCUUCUGUCAUC

>ath-miR157b-5p MIMAT0000173

UUGACAGAAGAUAGAGAGCAC

>ath-miR157d MIMAT0000175

UGACAGAAGAUAGAGAGCAC

>ath-miR158b MIMAT0001014

CCCCAAAUGUAGACAAAGCA

>ath-miR160b MIMAT0000179

UGCCUGGCUCCCUGUAUGCCA

>ath-miR160c-3p MIMAT0031875

CGUACAAGGAGUCAAGCAUGA

>ath-miR160c-5p MIMAT0000180

UGCCUGGCUCCCUGUAUGCCA

>ath-miR162a-5p MIMAT0031876

UGGAGGCAGCGGUUCAUCGAUC

>ath-miR162b-3p MIMAT0000183

UCGAUAAACCUCUGCAUCCAG

>ath-miR162b-5p MIMAT0031877

UGGAGGCAGCGGUUCAUCGAUC

>ath-miR163 MIMAT0000184

UUGAAGAGGACUUGGAACUUCGAU

>ath-miR164a MIMAT0000185

UGGAGAAGCAGGGCACGUGCA

>ath-miR164b-3p MIMAT0031878

CAUGUGCCCAUCUUCACCAUC

>ath-miR164b-5p MIMAT0000186

UGGAGAAGCAGGGCACGUGCA

>ath-miR164c-3p MIMAT0031916

CACGUGUUCUACUACUCCAAC

>ath-miR164c-5p MIMAT0001017

UGGAGAAGCAGGGCACGUGCG

>ath-miR165a-3p MIMAT0000187

UCGGACCAGGCUUCAUCCCCC

>ath-miR165a-5p MIMAT0031879

GGAAUGUUGUCUGGAUCGAGG

>ath-miR165b MIMAT0000188

UCGGACCAGGCUUCAUCCCCC

>ath-miR166a-5p MIMAT0031880

GGACUGUUGUCUGGCUCGAGG

>ath-miR166b-5p MIMAT0031881

GGACUGUUGUCUGGCUCGAGG

>ath-miR166e-5p MIMAT0031882

GGAAUGUUGUCUGGCACGAGG

>ath-miR167c-3p MIMAT0031917

UAGGUCAUGCUGGUAGUUUCACC

>ath-miR167c-5p MIMAT0001018

UAAGCUGCCAGCAUGAUCUUG

>ath-miR168b-3p MIMAT0031885

CCCGUCUUGUAUCAACUGAAU

>ath-miR168b-5p MIMAT0000199

UCGCUUGGUGCAGGUCGGGAA

>ath-miR169a-3p MIMAT0031886

GGCAAGUUGUCCUUGGCUAC

>ath-miR169d MIMAT0000908

UGAGCCAAGGAUGACUUGCCG

>ath-miR169f-3p MIMAT0031898

GCAAGUUGACCUUGGCUCUGC

>ath-miR169f-5p MIMAT0000910

UGAGCCAAGGAUGACUUGCCG

>ath-miR169h MIMAT0000913

UAGCCAAGGAUGACUUGCCUG

>ath-miR169i MIMAT0000914

UAGCCAAGGAUGACUUGCCUG

>ath-miR169j MIMAT0000915

UAGCCAAGGAUGACUUGCCUG

>ath-miR169k MIMAT0000916

UAGCCAAGGAUGACUUGCCUG

>ath-miR169m MIMAT0000918

UAGCCAAGGAUGACUUGCCUG

>ath-miR169n MIMAT0000919

UAGCCAAGGAUGACUUGCCUG

>ath-miR170-3p MIMAT0000201

UGAUUGAGCCGUGUCAAUAUC

>ath-miR170-5p MIMAT0031887

UAUUGGCCUGGUUCACUCAGA

>ath-miR171a-5p MIMAT0031888

UAUUGGCCUGGUUCACUCAGA

>ath-miR172a MIMAT0000203

AGAAUCUUGAUGAUGCUGCAU

>ath-miR172b-5p MIMAT0000204

GCAGCACCAUUAAGAUUCAC

>ath-miR172d-3p MIMAT0000923

AGAAUCUUGAUGAUGCUGCAG

>ath-miR172d-5p MIMAT0031901

GCAACAUCUUCAAGAUUCAGA

>ath-miR172e-3p MIMAT0001019

GGAAUCUUGAUGAUGCUGCAU

>ath-miR172e-5p MIMAT0031918

GCAGCACCAUUAAGAUUCAC

>ath-miR173-3p MIMAT0022843

UGAUUCUCUGUGUAAGCGAAA

>ath-miR173-5p MIMAT0000206

UUCGCUUGCAGAGAGAAAUCAC

>ath-miR319b MIMAT0000512

UUGGACUGAAGGGAGCUCCCU

>ath-miR390a-3p MIMAT0031902

CGCUAUCCAUCCUGAGUUUCA

>ath-miR390a-5p MIMAT0000931

AAGCUCAGGAGGGAUAGCGCC

>ath-miR390b-3p MIMAT0031903

CGCUAUCCAUCCUGAGUUCC

>ath-miR393a-3p MIMAT0031905

AUCAUGCUAUCUCUUUGGAUU

>ath-miR393a-5p MIMAT0000934

UCCAAAGGGAUCGCAUUGAUCC

>ath-miR393b-3p MIMAT0031906

AUCAUGCGAUCUCUUUGGAUU

>ath-miR393b-5p MIMAT0000935

UCCAAAGGGAUCGCAUUGAUCC

>ath-miR394a MIMAT0000936

UUGGCAUUCUGUCCACCUCC

>ath-miR394b-3p MIMAT0031907

AGGUGGGCAUACUGCCAAUAG

>ath-miR394b-5p MIMAT0000937

UUGGCAUUCUGUCCACCUCC

>ath-miR398a-3p MIMAT0000948

UGUGUUCUCAGGUCACCCCUU

>ath-miR398a-5p MIMAT0031910

AAGGAGUGGCAUGUGAACACA

>ath-miR398b-3p MIMAT0000949

UGUGUUCUCAGGUCACCCCUG

>ath-miR398b-5p MIMAT0031911

AGGGUUGAUAUGAGAACACAC

>ath-miR398c-3p MIMAT0000950

UGUGUUCUCAGGUCACCCCUG

>ath-miR398c-5p MIMAT0031912

AGGGUUGAUAUGAGAACACAC

>ath-miR399c-3p MIMAT0000953

UGCCAAAGGAGAGUUGCCCUG

>ath-miR399c-5p MIMAT0031913

GGGCAUCUUUCUAUUGGCAGG

>ath-miR400 MIMAT0001001

UAUGAGAGUAUUAUAAGUCAC

>ath-miR403-3p MIMAT0001004

UUAGAUUCACGCACAAACUCG

>ath-miR403-5p MIMAT0031914

UGUUUUGUGCUUGAAUCUAAUU

>ath-miR408-5p MIMAT0031915

ACAGGGAACAAGCAGAGCAUG

>ath-miR5663-3p MIMAT0032129

UGAGAAUGCAAAUCCUUAGCU

>ath-miR5663-5p MIMAT0022440

AGCUAAGGAUUUGCAUUCUCA

>ath-miR822-3p MIMAT0032018

UGUGCAAAUGCUUUCUACAGG

>ath-miR822-5p MIMAT0004239

UGCGGGAAGCAUUUGCACAUG

>ath-miR837-3p MIMAT0004259

AAACGAACAAAAAACUGAUGG

>ath-miR837-5p MIMAT0004258

AUCAGUUUCUUGUUCGUUUCA

>ath-miR840-3p MIMAT0032021

UUGUUUAGGUCCCUUAGUUUC

>ath-miR840-5p MIMAT0004262

ACACUGAAGGACCUAAACUAAC

>ath-miR844-3p MIMAT0004267

UUAUAAGCCAUCUUACUAGUU

>ath-miR844-5p MIMAT0004266

UGGUAAGAUUGCUUAUAAGCU

>ath-miR846-3p MIMAT0004269

UUGAAUUGAAGUGCUUGAAUU

>ath-miR846-5p MIMAT0032023

CAUUCAAGGACUUCUAUUCAG

>ath-miR851-3p MIMAT0004274

UGGGUGGCAAACAAAGACGAC

>ath-miR851-5p MIMAT0004273

UCUCGGUUCGCGAUCCACAAG

>ath-miR864-3p MIMAT0004312

UAAAGUCAAUAAUACCUUGAAG

>ath-miR864-5p MIMAT0004311

UCAGGUAUGAUUGACUUCAAA

>cpa-miR162a MIMAT0005567

UCGAUAAACCUCUGCAUCCAG

>cre-miR1151b-3p MIMAT0005396

ACGGGUUGUGGGACCCGGAC

>cre-miR1151b-5p MIMAT0005395

UCCGGGGCUCAUAACCUGUUA

>cre-miR1152 MIMAT0005397

UAAGAAGGUGCGCUGUCUUGA

>cre-miR1154-3p MIMAT0005403

CGCCUUGUGACGACUAAGU

>cre-miR1154-5p MIMAT0005402

ACUUAGUCAUCCCAAGGCGU

>cre-miR1155 MIMAT0005404

UAGUCCUGCACGAGGAAGGAGC

>cre-miR1157-3p MIMAT0005408

UUCAGGUAGCGGGACCAGGUG

>cre-miR1157-5p MIMAT0005407

ACCUGGUCCCGCUAUUUGAAUC

>cre-miR1162-3p MIMAT0005416

UGUUGUAGUAGUUUAGCCCUGC

>cre-miR1162-5p MIMAT0005415

CGGCCUAAAUUACUACAACACG

>cre-miR1165-3p MIMAT0005421

ACGGACCGCUUGUACGGAUAUG

>cre-miR1165-5p MIMAT0005420

UACCGUACAAGCGGUCCGUCC

>cre-miR911 MIMAT0004394

ACAAUGGUAGUACGGCCAUUU

>csi-miR156 MIMAT0018448

UGACAGAAGAGAGUGAGCAC

>csi-miR160 MIMAT0014068

GCCUGGCUCCCUGUAUGCCAU

>csi-miR164 MIMAT0014069

UGGAGAAGCAGGGCACGUGCA

>csi-miR166a MIMAT0014090

UCGGACCAGGCUUCAUUCCCCC

>csi-miR166d MIMAT0018452

UCGGACCAGGCUUCAUUCCCU

>csi-miR166e-5p MIMAT0017386

GGAAUGUUGUCUGGCUCGAGG

>csi-miR167a MIMAT0018498

UGAAGCUGCCAGCAUGAUCUG

>csi-miR167b MIMAT0018454

UGAAGCUGCCAGCAUGAUCUU

>csi-miR167c MIMAT0018453

UGAAGCUGCCAGCAUGAUCUG

>csi-miR172a-5p MIMAT0017387

GCAGCGUCCUCAAGAUUCACA

>csi-miR396c MIMAT0018493

UUCAAGAAAUCUGUGGGAAG

>csi-miR477a MIMAT0018475

ACCUCCCUCGAAGGCUUCCAA

>csi-miR479 MIMAT0018478

UGUGAUAUUGGUUCGGCUCAUC

>csi-miR482a-3p MIMAT0018480

UCUUCCCUAUGCCUCCCAUUCC

>csi-miR482a-5p MIMAT0018479

AGUGGGAGCGUGGGGUAAGAAG

>csi-miR482b MIMAT0018481

UCUUGCCCACCCCUCCCAUUCC

>csi-miR482c MIMAT0018495

UUCCCUAGUCCCCCUAUUCCUA

>gar-miR2947 MIMAT0014330

UAUACCGUGCCCAUGACUGUAG

>hvu-miR156b MIMAT0035815

UGACAGAAGAGAGUGAGCACA

>hvu-miR159a MIMAT0018210

UUUGGAUUGAAGGGAGCUCUG

>nta-miR156g MIMAT0024635

UGACAGAAGAUAGAGAGCAC

>nta-miR156h MIMAT0024636

UGACAGAAGAUAGAGAGCAC

>nta-miR156i MIMAT0024637

UGACAGAAGAUAGAGAGCAC

>nta-miR156j MIMAT0024638

UGACAGAAGAUAGAGAGCAC

>nta-miR160d MIMAT0024643

UGCCUGGCUCCCUGCAUGCCA

>nta-miR166h MIMAT0024656

UCGGACCAGGCUUCAUUCCCC

>nta-miR167a MIMAT0024657

UGAAGCUGCCAGCAUGAUCUGG

>nta-miR167e MIMAT0024661

UGAAGCUGCCAGCAUGAUCUA

>nta-miR168a MIMAT0024662

UCGCUUGGUGCAGGUCGGGAC

>nta-miR168b MIMAT0024663

UCGCUUGGUGCAGGUCGGGAC

>nta-miR168c MIMAT0024664

UCGCUUGGUGCAGGUCGGGAC

>nta-miR168e MIMAT0024666

UCGCUUGGUGCAGGUCGGGAA

>nta-miR169r MIMAT0024683

CAGCCAAGGAUGACUUGCCGG

>nta-miR172a MIMAT0024689

AGAAUCUUGAUGAUGCUGCAG

>nta-miR172d MIMAT0024692

AGAAUCUUGAUGAUGCUGCAU

>nta-miR172e MIMAT0024693

AGAAUCUUGAUGAUGCUGCAU

>nta-miR390b MIMAT0024702

AAGCUCAGGAGGGAUAGCGCC

>nta-miR390c MIMAT0024703

AAGCUCAGGAGGGAUAGCGCC

>nta-miR396b MIMAT0024709

UUCCACAGCUUUCUUGAACUU

>nta-miR396c MIMAT0024710

UUCCACAGCUUUCUUGAACUU

>nta-miR482c MIMAT0024727

UUUCCAAUUCCACCCAUUCCUA

>nta-miR482d MIMAT0024776

UUCCCGACUCCCCCCAUACCAC

>nta-miR6020a-3p MIMAT0023585

AGAUACUCAGCAAAACAUUUAC

>nta-miR6020a-5p MIMAT0023584

AAAUGUUUUUCGAGUAUCUUC

>nta-miR6020b MIMAT0023587

AAAUGUUCUUCGAGUAUCUUC

>nta-miR6025a MIMAT0023606

UACCAACAAUUGAGAUAACAUC

>nta-miR6144 MIMAT0024729

UGGCAACUUCUUCAUCAUGCC

>nta-miR6145a MIMAT0024731

CAUUUUCACAUGUAGCACUGAC

>nta-miR6145c MIMAT0024758

CAGUGCACAUAUAACAGUAA

>nta-miR6145d MIMAT0024764

AUUGUUACAUGUAACACUGGC

>nta-miR6145f MIMAT0024771

AUCGUAACAUAUAGCACUAGC

>nta-miR6146a MIMAT0024734

UUUGUCCAAUGAAACACUUAUC

>nta-miR6146b MIMAT0024735

UUUGUCCAAUGAAAUACUUAUC

>nta-miR6147 MIMAT0024736

UGACAUCUUCAAAACCCACUA

>nta-miR6149a MIMAT0024739

UUGAUACGCACCUGAAUCGGC

>nta-miR6149b MIMAT0024740

UUGAUACGCACCUGAAUCGGC

>nta-miR6154a MIMAT0024755

UGGGUCUCCUGGAGAAAGGUC

>nta-miR6155 MIMAT0024757

UAAGGUUGCCUUGCUCUUGCA

>nta-miR6159 MIMAT0024769

UAGCAUAGAAUUCUCGCACCUA

>nta-miR827 MIMAT0024725

UUAGAUGAACAUCAACAAACA

>osa-miR1320-3p MIMAT0015286

UGUAAAAUUCAUUCGUUCCAA

>osa-miR1320-5p MIMAT0009137

UGGAACGGAGGAAUUUUAUAG

>osa-miR1425-3p MIMAT0020933

CAGCAAGAACUGGAUCUUAAU

>osa-miR1425-5p MIMAT0005959

UAGGAUUCAAUCCUUGCUGCU

>osa-miR1430 MIMAT0005964

UGGUGAGCCUUCCUGGCUAAG

>osa-miR1432-3p MIMAT0022950

CAGGUGUCAUCUCCCCUGAAC

>osa-miR1432-5p MIMAT0005966

AUCAGGAGAGAUGACACCGAC

>osa-miR156b-3p MIMAT0022845

GCUCACUCUCUAUCUGUCAGC

>osa-miR156c-3p MIMAT0022846

GCUCACUUCUCUCUCUGUCAGC

>osa-miR156f-3p MIMAT0022847

GCUCACUUCUCUUUCUGUCAGC

>osa-miR156g-3p MIMAT0022848

GCUCACUUCUCUCUCUGUCAGC

>osa-miR156g-5p MIMAT0000624

UGACAGAAGAGAGUGAGCAC

>osa-miR156h-3p MIMAT0031157

GCUCACUUCUCUUUCUGUCAGC

>osa-miR156h-5p MIMAT0031156

UGACAGAAGAGAGUGAGCAC

>osa-miR156i MIMAT0000626

UGACAGAAGAGAGUGAGCAC

>osa-miR156j-3p MIMAT0022850

GCUCGCUCCUCUUUCUGUCAGC

>osa-miR156j-5p MIMAT0000627

UGACAGAAGAGAGUGAGCAC

>osa-miR156l-3p MIMAT0022866

GCUCACUUCUCUUUCUGUCAGC

>osa-miR156l-5p MIMAT0001021

CGACAGAAGAGAGUGAGCAUA

>osa-miR159d MIMAT0001025

AUUGGAUUGAAGGGAGCUCCG

>osa-miR160a-3p MIMAT0022851

GCGUGCAAGGAGCCAAGCAUG

>osa-miR160b-3p MIMAT0022852

GCGUGCAAGGAGCCAAGCAUG

>osa-miR160c-3p MIMAT0022853

GCGUGCACGGAGCCAAGCAUA

>osa-miR160d-3p MIMAT0022854

GCGUGCGAGGAGCCAAGCAUG

>osa-miR160d-5p MIMAT0000631

UGCCUGGCUCCCUGUAUGCCA

>osa-miR160e-3p MIMAT0022867

GCGUGCGAGGUGCCAAGCAUG

>osa-miR160e-5p MIMAT0001030

UGCCUGGCUCCCUGUAUGCCG

>osa-miR160f-3p MIMAT0022868

GCAUUGAGGGAGUCAUGCAGG

>osa-miR160f-5p MIMAT0001031

UGCCUGGCUCCCUGAAUGCCA

>osa-miR162b MIMAT0001032

UCGAUAAGCCUCUGCAUCCAG

>osa-miR164d MIMAT0001034

UGGAGAAGCAGGGCACGUGCU

>osa-miR166a-5p MIMAT0022855

GGAAUGUUGUCUGGUUCAAGG

>osa-miR166e-3p MIMAT0000639

UCGAACCAGGCUUCAUUCCCC

>osa-miR166e-5p MIMAT0022859

GGAAUGUUGUCUGGUUCAAGG

>osa-miR166g-3p MIMAT0001072

UCGGACCAGGCUUCAUUCCUC

>osa-miR166g-5p MIMAT0022881

AAUGGAGGCUGAUCCAAGAUC

>osa-miR166h-3p MIMAT0001073

UCGGACCAGGCUUCAUUCCUC

>osa-miR166h-5p MIMAT0022882

GGAAUGUUGGCUGGCUCGAGG

>osa-miR166i-3p MIMAT0001074

UCGGAUCAGGCUUCAUUCCUC

>osa-miR166i-5p MIMAT0022883

AAUGCAGUUUGAUCCAAGAUC

>osa-miR166j-3p MIMAT0001088

UCGGACCAGGCUUCAUUCCCC

>osa-miR166j-5p MIMAT0022887

GAAUGACGUCCGGUCUGAAGA

>osa-miR166k-3p MIMAT0001037

UCGGACCAGGCUUCAAUCCCU

>osa-miR166k-5p MIMAT0022870

GGUUUGUUGUCUGGCUCGAGG

>osa-miR166l-3p MIMAT0001038

UCGGACCAGGCUUCAAUCCCU

>osa-miR166l-5p MIMAT0022871

GGAUUGUUGUCUGGUUCAAGG

>osa-miR167a-3p MIMAT0006780

AUCAUGCAUGACAGCCUCAUUU

>osa-miR167b MIMAT0000642

UGAAGCUGCCAGCAUGAUCUA

>osa-miR167c-3p MIMAT0022860

GGUCAUGCUGCGGCAGCCUCACU

>osa-miR167c-5p MIMAT0000643

UGAAGCUGCCAGCAUGAUCUA

>osa-miR167d-3p MIMAT0022872

GAUCAUGCUGUGCAGUUUCAUC

>osa-miR167d-5p MIMAT0001039

UGAAGCUGCCAGCAUGAUCUG

>osa-miR167e-3p MIMAT0022873

AGAUCAUGUUGCAGCUUCACU

>osa-miR167f MIMAT0001041

UGAAGCUGCCAGCAUGAUCUG

>osa-miR167h-3p MIMAT0022874

AGGUCAUGCUGUAGUUUCAUC

>osa-miR167h-5p MIMAT0001043

UGAAGCUGCCAGCAUGAUCUG

>osa-miR167i-3p MIMAT0022875

AGAUCAUGUUGCAGCUUCACU

>osa-miR167i-5p MIMAT0001044

UGAAGCUGCCAGCAUGAUCUG

>osa-miR169c MIMAT0001048

CAGCCAAGGAUGACUUGCCGG

>osa-miR169g MIMAT0001052

UAGCCAAGGAUGACUUGCCUA

>osa-miR169i-3p MIMAT0022876

UGAGUCGCUCUUAUCACUCAUG

>osa-miR169i-5p.2 MIMAT0020917

UGGUGAUAAGGGUGUAGCUCUG

>osa-miR169l MIMAT0001057

UAGCCAAGGAUGACUUGCCUG

>osa-miR169n MIMAT0001059

UAGCCAAGAAUGACUUGCCUA

>osa-miR169o MIMAT0001060

UAGCCAAGAAUGACUUGCCUA

>osa-miR169r-3p MIMAT0005967

UGGCAAGUCUCCUCGGCUACC

>osa-miR169r-5p MIMAT0026762

UAGCCAAGGAUGAUUUGCCUG

>osa-miR171a MIMAT0000645

UGAUUGAGCCGCGCCAAUAUC

>osa-miR171d-3p MIMAT0001065

UGAUUGAGCCGUGCCAAUAUC

>osa-miR171d-5p MIMAT0022878

UGUUGGCCCGGCUCACUCAGA

>osa-miR171e-3p MIMAT0001066

UGAUUGAGCCGUGCCAAUAUC

>osa-miR171e-5p MIMAT0022879

UGUUGGCUCGGCUCACUCAGA

>osa-miR171f-3p MIMAT0001067

UGAUUGAGCCGUGCCAAUAUC

>osa-miR171f-5p MIMAT0022880

UGUUGGCAUGGUUCAAUCAAA

>osa-miR171h MIMAT0001077

GUGAGCCGAACCAAUAUCACU

>osa-miR172c MIMAT0001071

UGAAUCUUGAUGAUGCUGCAC

>osa-miR172d-5p MIMAT0022885

GCAGCACCAUCAAGAUUCAC

>osa-miR1846a-3p MIMAT0007789

UGACCCCGUUCUCCUCGCCGG

>osa-miR1846a-5p MIMAT0007788

AGUGAGGAGGCCGGGGCCGCU

>osa-miR1846b-3p MIMAT0007791

UGACCCCGUUCUCCUCGCCGG

>osa-miR1846b-5p MIMAT0007790

AGUGAGGAGGCCGGGGCCGCU

>osa-miR1846d-3p MIMAT0007765

UAUCCGGCGCCGCAGGGAGG

>osa-miR1846d-5p MIMAT0007764

UCCCACCGAGCAGCCGGAUCUC

>osa-miR1850.1 MIMAT0007770

UGGAAAGUUGGGAGAUUGGGG

>osa-miR1850.2 MIMAT0009210

UUGUGUGUGAACUAAACGUGG

>osa-miR1850.3 MIMAT0009211

CUGUUUAGUUCACAUCAAUCUU

>osa-miR1859 MIMAT0007792

UUUCCUAUGACGUCCAUUCCAA

>osa-miR1860-3p MIMAT0007794

AUCUGGAAGCUAGGUUUUCUCU

>osa-miR1860-5p MIMAT0007793

AGAAAACCAGCUUCCAGAUCU

>osa-miR1861a MIMAT0007796

UGAUCUUGAGGCAGAAACUGAG

>osa-miR1861h MIMAT0007803

CGGUCUUGAGGCAGGAACUGAG

>osa-miR1862e MIMAT0007833

CUAGAUUUGUUUAUUUUGGGACGG

>osa-miR1863c MIMAT0013830

UAGAAACUUGGCUGAUGCAUUACU

>osa-miR1865-3p MIMAT0007816

CGAAGAAUCGCAGUCACUAGUUGU

>osa-miR1865-5p MIMAT0007815

UGCUAGUGAUGGUGAUUCUUCGAC

>osa-miR1878 MIMAT0007835

ACUUAAUCUGGACACUAUAAAAGA

>osa-miR1882e-3p MIMAT0015285

GAAAUGAUCUUGGACGUAAUCUAG

>osa-miR1882e-5p MIMAT0007843

AGAUUGCUUUCAAGGUCAUUUCUU

>osa-miR2096-3p MIMAT0010057

CCUGAGGGGAAAUCGGCGGGA

>osa-miR2096-5p MIMAT0010056

UGCCGAUUUCCCCCUCGGGCG

>osa-miR2118r MIMAT0011757

UUCCCAAUGCCUCCCAUGCCUA

>osa-miR2275a MIMAT0011758

UUUGGUUUCCUCCAAUAUCUCA

>osa-miR2275c MIMAT0022147

AGAAUUGGAGGAAAACAAACUGA

>osa-miR2866 MIMAT0013813

UCUAGUUUGUGUUCAGCAUC

>osa-miR319a-3p MIMAT0020915

ACUGGAUGACGCGGGAGCUAA

>osa-miR319a-5p MIMAT0020914

AGCUGCCGAAUCAUCCAUUCA

>osa-miR390-3p MIMAT0022888

CGCUAUCUAUCCUGAGCUCC

>osa-miR393a MIMAT0000957

UCCAAAGGGAUCGCAUUGAUC

>osa-miR394 MIMAT0000958

UUGGCAUUCUGUCCACCUCC

>osa-miR395b MIMAT0000959

GUGAAGUGUUUGGGGGAACUC

>osa-miR395d MIMAT0000960

GUGAAGUGUUUGGGGGAACUC

>osa-miR395h MIMAT0000963

GUGAAGUGUUUGGGGGAACUC

>osa-miR395i MIMAT0000964

GUGAAGUGUUUGGGGGAACUC

>osa-miR395j MIMAT0000965

GUGAAGUGUUUGGGGGAACUC

>osa-miR395k MIMAT0000966

GUGAAGUGUUUGGGGGAACUC

>osa-miR395l MIMAT0000967

GUGAAGUGUUUGGGGGAACUC

>osa-miR395n MIMAT0003871

GUGAAGUGUUUGGGGGAACUC

>osa-miR395p MIMAT0003873

GUGAAGUGUUUGGGGGAACUC

>osa-miR395q MIMAT0003874

GUGAAGUGUUUGGGGGAACUC

>osa-miR395r MIMAT0003878

GUGAAGUGUUUGGGGGAACUC

>osa-miR395s MIMAT0000968

GUGAAGUGUUUGGGGGAACUC

>osa-miR396a-3p MIMAT0022863

GUUCAAUAAAGCUGUGGGAA

>osa-miR396b-3p MIMAT0022864

GUUCAAUAAAGCUGUGGGAA

>osa-miR396c-3p MIMAT0022865

GGUCAAGAAAGCUGUGGGAAG

>osa-miR3979-3p MIMAT0019674

CUUCGGGGGAGGAGAGAAGC

>osa-miR3979-5p MIMAT0019673

UCUCUCUCUCCCUUGAAGGC

>osa-miR399c MIMAT0000986

UGCCAAAGGAGAAUUGCCCUG

>osa-miR399i MIMAT0000992

UGCCAAAGGAGAGCUGCCCUG

>osa-miR408-3p MIMAT0001079

CUGCACUGCCUCUUCCCUGGC

>osa-miR408-5p MIMAT0022884

CAGGGAUGAGGCAGAGCAUGG

>osa-miR444f MIMAT0005976

UGCAGUUGUUGCCUCAAGCUU

>osa-miR5143a MIMAT0021087

UGUGGUAUGUUGGCAAUGUAGGAA

>osa-miR5150-3p MIMAT0021099

AGAAGCUGCAGCUGUCAGAAGCUC

>osa-miR5150-5p MIMAT0021098

AGCUUCUGACAGCUGCAGUUUCUC

>osa-miR5152-3p MIMAT0021102

AGACCAUGCCUAUACCUACCA

>osa-miR5152-5p MIMAT0021101

GUAGGGAUAGGCAUGAUCUCU

>osa-miR5157a-3p MIMAT0021108

AGAAGUUGUGGCUAUCAAAAAGUU

>osa-miR5157a-5p MIMAT0021107

AACUUUUUAAUAGCUACAACUUCU

>osa-miR5157b-3p MIMAT0021110

AGAAGUUGUGGCUAUCAAAAAGUU

>osa-miR5157b-5p MIMAT0021109

AACUUUUUAAUAGCUACAACUUCU

>osa-miR528-3p MIMAT0022926

CCUGUGCUUGCCUCUUCCAUU

>osa-miR528-5p MIMAT0002884

UGGAAGGGGCAUGCAGAGGAG

>osa-miR529b MIMAT0005015

AGAAGAGAGAGAGUACAGCUU

>osa-miR530-3p MIMAT0002886

AGGUGCAGAGGCAGAUGCAAC

>osa-miR530-5p MIMAT0006787

UGCAUUUGCACCUGCACCUA

>osa-miR535-3p MIMAT0022927

GUGCUUUCUCCCGUUGUCACU

>osa-miR535-5p MIMAT0003142

UGACAACGAGAGAGAGCACGC

>osa-miR5485 MIMAT0022118

UGACAACUGGUAGCAGAGCAA

>osa-miR5497 MIMAT0022130

CAGAAUAUCUGGGACGAGCAU

>osa-miR5513 MIMAT0022146

UAACAAAGGACAACAGACUGA

>osa-miR5518 MIMAT0022152

AUACUCAAACAGGGCAUUGCA

>osa-miR5792 MIMAT0023259

GAUGACAGCGGUGGUUCGGACAUC

>osa-miR5820 MIMAT0023293

UGGCAGAGAUUGAUCGAGGAA

>osa-miR5825 MIMAT0023299

AAAACAUCUGAUAACCUGAAACGG

>osa-miR5828 MIMAT0023302

UUACGGCAUUAAAAGUAAAUC

>pab-miR160a MIMAT0018146

UGCCUGGCUCCCUGUAUGCCA

>pab-miR3695 MIMAT0018127

UUGUCGCCUGGUCGUUGUGGG

>pab-miR3697 MIMAT0018129

UAGCCCCUGACUUCAACAUGAG

>pab-miR396a MIMAT0018152

UUCCACAGCUUUCUUGAACUA

>pab-miR482a MIMAT0018156

UCUUCCCUACUCCUCCCAUUCC

>pab-miR482b MIMAT0018157

UCUUCCCUAUUCCUCCCAUUCC

>pab-miR482c MIMAT0018158

UCUUUCCUACUCCUCCCAUUCC

>pab-miR951 MIMAT0018161

UGUUCUUGACGUCUGGACCACG

>ppt-miR1023a-3p MIMAT0005103

AGAGAAUUGGAGAGAGUGCAU

>ppt-miR1023a-5p MIMAT0005102

ACACUCUCUCCAUUUCUCUAC

>ppt-miR1023b-3p MIMAT0005105

AGAGAAUUGAAGAGAGUGCAU

>ppt-miR1023b-5p MIMAT0005104

ACACUCUCUCCAUUUCUCUGC

>ppt-miR1023c-3p MIMAT0005107

AGGGAAUCGGAAAGAGUGUAC

>ppt-miR1023c-5p MIMAT0005106

CCACUCUCUCCGUUUCCCUUCC

>ppt-miR1023e-3p MIMAT0010025

AGGGAAUCGGGUGGAGUGCAU

>ppt-miR1023e-5p MIMAT0010024

ACACUCUCUCCGUUUCCCUUC

>ppt-miR1024a MIMAT0005109

UCUGGUUGGAUUGUAGGCCUC

>ppt-miR1024b MIMAT0005110

UCUGGUUGGAUUGUAGGCCUC

>ppt-miR1025 MIMAT0005111

UGCCACAACAAAGCUAAUAAC

>ppt-miR1026b MIMAT0005113

UGAGAAAGACUUGAGAGGACA

>ppt-miR1028a-3p MIMAT0005117

UGACAUUGUAGAUCUACGUGC

>ppt-miR1028a-5p MIMAT0005116

UCUUAGAUCUACAAUGUCACA

>ppt-miR1028b-3p MIMAT0005119

CGGCAUUGUGGACCUAAGACC

>ppt-miR1028b-5p MIMAT0005118

UCUUAGAUCUACAAUGCCACC

>ppt-miR1028c-3p MIMAT0005121

UGGCAUUGUAGGUUUAAGAGC

>ppt-miR1028c-5p MIMAT0005120

UCUUAGAUCUACAAUGCCUCU

>ppt-miR1030a MIMAT0005123

UCUGCAUCUGCACCUGCACCA

>ppt-miR1030b MIMAT0005124

UCUGCAUCUGCACCUGCACCA

>ppt-miR1030c MIMAT0005125

UCUGCAUCUGCACCUGCACCA

>ppt-miR1030e MIMAT0005127

UCUGCAUCUGCACCUGCACCA

>ppt-miR1030f MIMAT0005128

UCUGCAUCUGCACCUGCACCA

>ppt-miR1030h MIMAT0005130

UCUGCAUCUGCACCUGCACCG

>ppt-miR1034 MIMAT0005141

UUACUUUGGCAGCGCUGUGCU

>ppt-miR1035 MIMAT0005142

CGUCUUAGCCCACAAAACGAA

>ppt-miR1036-3p MIMAT0005144

AGCUAAUUAAGGAUUCUACAC

>ppt-miR1036-5p MIMAT0005143

UGUGGAGUCCGUAAUUAGCUG

>ppt-miR1038-3p MIMAT0005147

CAUGGUGGAAUCGCAUCCAGG

>ppt-miR1038-5p MIMAT0005146

UAGGUGCGUUUCCACCAAAG

>ppt-miR1040 MIMAT0005150

UGAACGCAAAUGAACAUGUUC

>ppt-miR1044-3p MIMAT0005157

UUGUAGUGCAUAUUUGUUUU

>ppt-miR1044-5p MIMAT0005156

GCACAAAUAUGCACUACAAAC

>ppt-miR1047-3p MIMAT0005162

UGAUCAGCGAUGACCUAGUUG

>ppt-miR1047-5p MIMAT0005161

ACUAGGUAGUCCUUGAUUACA

>ppt-miR1048-3p MIMAT0005164

UGUCUACACUCAUGUUCUAGA

>ppt-miR1048-5p MIMAT0005163

UAGAACAUGAGUGUAGACGAC

>ppt-miR1050 MIMAT0005166

UGACCACCUUGAUUCCGGCCU

>ppt-miR1052 MIMAT0005169

UUCCUUUUAUUGAUUGUGGUA

>ppt-miR1058 MIMAT0005176

AGAGAUUCCAUCACGAAGCAC

>ppt-miR1059 MIMAT0005177

UGAAAGUCCUUCACAAACAAC

>ppt-miR1063b MIMAT0005183

CAUCUUGGAGUACUGCAUCUU

>ppt-miR1063c MIMAT0005184

CAUCUUGGAGUACUGCAUCUU

>ppt-miR1063d MIMAT0005185

CAUCUUGGAGUACUGCAUCUU

>ppt-miR1063f MIMAT0005187

CAUCUUGGAGUACUGCAUCUU

>ppt-miR1063g MIMAT0005188

CAUCUUGGAGUACUGCAUCUU

>ppt-miR1065 MIMAT0005192

ACAGUCUCUGACUUCUCGCAG

>ppt-miR1072 MIMAT0005201

UGCAUUGUGUUAUUUGAAGCUUGA

>ppt-miR1073-3p MIMAT0005203

UGGGCGUUAAUACAUUUCAUA

>ppt-miR1073-5p MIMAT0005202

UGAAUGAUAUUAACGUCCACG

>ppt-miR1211-3p MIMAT0004829

UGCAUGACCGUCUCUUCCUGC

>ppt-miR1211-5p MIMAT0003900

AGGGAGGGAUGGUUAUGCAAG

>ppt-miR1212 MIMAT0003901

CGUGGGACAGCAUAGAAUGCG

>ppt-miR1214 MIMAT0003903

UACUAUGAGAAUCUCGCGGCC

>ppt-miR1216 MIMAT0003905

UGAUGGUGAUGCGCUUGUAUC

>ppt-miR1217-3p MIMAT0003906

AAUUUGAAGCAUGAUGUCAAG

>ppt-miR1217-5p MIMAT0004830

UGGUAUCAUGUUGCAAAUGGC

>ppt-miR1219a MIMAT0003908

CUUCCUGCCUCUCACUAGCUU

>ppt-miR1219b MIMAT0003909

CUUCCUGCCUCUCACUAGCUU

>ppt-miR1221-3p MIMAT0004834

UGGCCCAUGCACAGUAUCUACG

>ppt-miR1221-5p MIMAT0003916

UGGAUGGUGUGCAGGGUCAAA

>ppt-miR1222a MIMAT0003917

UUGAAGGAGUUCAUUGGUAUA

>ppt-miR1223a MIMAT0003918

UUGUAGAGUCAUACACCUCCA

>ppt-miR1223d MIMAT0005095

UUGUAGAGUCAUACACCUCU

>ppt-miR1223e MIMAT0005096

UUGUAGAGUCAUGCACCUCUG

>ppt-miR1223f MIMAT0005097

UUGUAGAGUCAUGCACCUCUG

>ppt-miR1223g MIMAT0005098

UUGUAGAGUCAUGCACCUCUG

>ppt-miR1223i MIMAT0005100

UUGUAGAGUCACGCACCUCUG

>ppt-miR1223j MIMAT0005101

UUGUAGAGUCAUGCACCUCUA

>ppt-miR156a MIMAT0003143

UGACAGAAGAGAGUGAGCAC

>ppt-miR156c MIMAT0004346

UGACAGAAGAGAGUGAGCAC

>ppt-miR160c MIMAT0004349

CGCCUGGCUCCCUGCAUGCCA

>ppt-miR160d MIMAT0004350

CGCCUGGCUCCCUGCAUGCCG

>ppt-miR160e MIMAT0005034

UGCCUGGCUCCCUGUAUGCCA

>ppt-miR160f MIMAT0005035

UGCCUGGCUCCCUGUAUGCCA

>ppt-miR160g MIMAT0005036

UGCCUGGCUCCUUGUAUGCCA

>ppt-miR166a MIMAT0004352

UCGGACCAGGCUUCAUUCCCC

>ppt-miR166b MIMAT0004351

UCGGACCAGGCUUCAUUCCCC

>ppt-miR166c MIMAT0005039

UCGGACCAGGCUUCAUUCCCC

>ppt-miR166d MIMAT0005040

UCGGACCAGGCUUCAUUCCCC

>ppt-miR166e MIMAT0005041

UCGGACCAGGCUUCAUUCCCC

>ppt-miR166f MIMAT0005042

UCGGACCAGGCUUCAUUCCCC

>ppt-miR166g MIMAT0005043

UCGGACCAGGCUUCAUUCCCC

>ppt-miR171a MIMAT0004375

UGAGCCGCGCCAAUAUCACAU

>ppt-miR2078 MIMAT0010015

GGUUGGCUUGCCUGUGCCUGU

>ppt-miR2079 MIMAT0010016

AGAGUUGAUGUUGAUGACGCA

>ppt-miR2080 MIMAT0010017

UCCAUAUCAAUUCGCAGAUGC

>ppt-miR2081 MIMAT0010020

AGCUAGAGUUAUGUGUCUGAU

>ppt-miR2082 MIMAT0010021

UGUGUGUUCCGCUUCUUCUUU

>ppt-miR319b MIMAT0003134

CUUGGACUGAAGGGAGCUCC

>ppt-miR390c-3p MIMAT0004327

CGCUGUCCAUUCUGAGCAUUG

>ppt-miR390c-5p MIMAT0003919

GAGCUCAGGAGGGAUAGCGCC

>ppt-miR477a-3p MIMAT0003899

AGAAGCCUUUGUGGGAGAGGA

>ppt-miR477a-5p MIMAT0004360

CUCUCCCUCAAAGGCUUCCA

>ppt-miR477g-3p MIMAT0003902

GUUGGAAGCCUUCGUGGGAGA

>ppt-miR477g-5p MIMAT0004362

UCCCUCAAAGGCUUCCAACAA

>ppt-miR529f MIMAT0005059

AGAAGAGAGAGAGUACAGCCC

>ppt-miR533a-3p MIMAT0004783

CUCACAGUCUGCACAGCUCUC

>ppt-miR533a-5p MIMAT0003137

GAGCUGGCCAGGCUGUGAGGG

>ppt-miR533c MIMAT0005061

CUCACAGUCUGCACAGCUCUC

>ppt-miR533e MIMAT0005063

CUCACAGUCUGCAUGGCUCUC

>ppt-miR534b MIMAT0004363

UAUGUCCAUUACAGUUGCAUAC

>ppt-miR535a MIMAT0003139

UGACAACGAGAGAGAGCACGC

>ppt-miR535d MIMAT0003915

UGACAACGAGAGAGAGCACGC

>ppt-miR537a MIMAT0003145

UUGAGGUGUUUCUACAGGCUA

>ppt-miR537d MIMAT0005068

UUGAGGUGUUUCUACAGGCUA

>ppt-miR538b MIMAT0003148

UUGCAUGGAGUCUAUGUCUGGA

>ppt-miR538c MIMAT0003149

UUGCAUGGAGUCUAUGUCUGGA

>ppt-miR898a-3p MIMAT0004383

GCUAGGCAGUGCACAGCGAUA

>ppt-miR898a-5p MIMAT0004382

UUGCUGUGCACUACUUAGUAC

>ppt-miR902c-3p MIMAT0005070

ACGAAGGUCUGCAUCAUAGU

>ppt-miR902c-5p MIMAT0005069

UAUGAUGCAGAUUCUUCAUCU

>ppt-miR902e-3p MIMAT0005074

ACGAAGGUCUGCAUCAUAGU

>ppt-miR902e-5p MIMAT0005073

UAUGAUGCAGAUUCUUCAUCU

>ppt-miR902g-3p MIMAT0005078

ACGAAGGUCUGCAUCAUAGU

>ppt-miR902g-5p MIMAT0005077

UAUGAUGCAGAUUCUUCAUCU

>ppt-miR902h-3p MIMAT0005080

AGAAGGGUCUACAUCAUAAAC

>ppt-miR902h-5p MIMAT0005079

UUAUGAUGUAGAUUCUUCAU

>ppt-miR902i-3p MIMAT0005082

UGGAGGAUCUGCAUCGUAAAC

>ppt-miR902i-5p MIMAT0005081

UUAUGAUGUAGAUUCUUCAU

>ppt-miR902j-3p MIMAT0005084

AGAAGGAUCUGCAACAUAGAC

>ppt-miR902j-5p MIMAT0005083

AUAUGUUGCAGAUUCUUCAUU

>ppt-miR902k-3p MIMAT0005086

ACGAAGGAUCUGCAAUAUAAA

>ppt-miR902k-5p MIMAT0005085

UAUGUUGCAGAUUCUUCAUUU

>ptc-miR1446d MIMAT0006007

UUCUGAACUCUCUCCCUCAA

>ptc-miR1447 MIMAT0006009

CAGAAUUGCAGUGCCUUGAUU

>ptc-miR1448 MIMAT0006010

CUUUCCAACGCCUCCCAUAC

>ptc-miR1450 MIMAT0006012

UUCAAUGGCUCGGUCAGGUUAC

>ptc-miR156f MIMAT0001895

UGACAGAAGAGAGUGAGCAC

>ptc-miR156j MIMAT0001899

UUGACAGAAGAUAGAGAGCAC

>ptc-miR160e-3p MIMAT0022892

GCAUGAGGGGAGUCGAGCAGG

>ptc-miR160e-5p MIMAT0001911

UGCCUGGCUCCCUGAAUGCCA

>ptc-miR164e MIMAT0001922

UGGAGAAGCAGGGCACGUGCA

>ptc-miR167g-3p MIMAT0022894

AGAUCAUGUGGCAGUUUCACC

>ptc-miR167g-5p MIMAT0001947

UGAAGCUGCCAGCAUGAUCUU

>ptc-miR168b-3p MIMAT0022897

CCCGCCUUGCAUCAACUGAAU

>ptc-miR169ac MIMAT0001954

UAGCCAAGGACGACUUGCCCA

>ptc-miR169ae MIMAT0001956

UAGCCAAGGACGACUUGCCCA

>ptc-miR169af MIMAT0001957

UAGCCAAGGACGACUUGCCCA

>ptc-miR169n-3p MIMAT0022899

GCAAGCAUCCUUGGUUCUCC

>ptc-miR169n-5p MIMAT0001970

UGAGCCAAGGAUGACUUGCCG

>ptc-miR169u-3p MIMAT0022900

GGCAGUCUCCUUUGGCUAUCC

>ptc-miR169u-5p MIMAT0001977

UAGCCAAGGACGACUUGCCUA

>ptc-miR171j MIMAT0005996

CGAGCCGAAUCAAUAUCACU

>ptc-miR171l-3p MIMAT0005994

CGAGCCGAAUCAAUAUCACU

>ptc-miR171l-5p MIMAT0002095

UGUGAUAUUGGUCCGGCUCAUC

>ptc-miR172c MIMAT0001995

AGAAUCUUGAUGAUGCUGCAU

>ptc-miR172g-3p MIMAT0001999

GGAAUCUUGAUGAUGCUGCAG

>ptc-miR172g-5p MIMAT0022905

GGAGCAUCAUCAAGAUUCACA

>ptc-miR2111a MIMAT0025270

UAAUCUGCAUCCUGAGGUUUG

>ptc-miR393b-3p MIMAT0022909

AUCAUGCUAUCCCUUUGGAUU

>ptc-miR393b-5p MIMAT0002016

UCCAAAGGGAUCGCAUUGAUC

>ptc-miR396a MIMAT0002031

UUCCACAGCUUUCUUGAACUG

>ptc-miR396b MIMAT0002032

UUCCACAGCUUUCUUGAACUG

>ptc-miR396d MIMAT0002034

UUCCACAGCUUUCUUGAACUU

>ptc-miR396e-3p MIMAT0022910

CUCAAGAAAGCUGUGGGAGA

>ptc-miR396e-5p MIMAT0002035

UUCCACAGCUUUCUUGAACUU

>ptc-miR396g-3p MIMAT0022911

CUCAAGAAAGCCGUGGGAAAA

>ptc-miR396g-5p MIMAT0002037

UUCCACGGCUUUCUUGAACUU

>ptc-miR398c-3p MIMAT0002043

UGUGUUCUCAGGUCGCCCCUG

>ptc-miR398c-5p MIMAT0022912

GGAGCGACCUGAAAUCACAUG

>ptc-miR403c-3p MIMAT0003944

UUAGAUUCACGCACAAACUCG

>ptc-miR403c-5p MIMAT0022932

UUUGUGCGUGGAUCUGAGGCC

>ptc-miR408-5p MIMAT0022913

CGGGGAACAGGCAGAGCAUGG

>ptc-miR473a-3p MIMAT0022914

UGAGGCCUUUGGGGGAGAGUGG

>ptc-miR473a-5p MIMAT0002062

ACUCUCCCUCAAGGCUUCCA

>ptc-miR477a-3p MIMAT0022918

GGAUGCCUUUGGGGGAGAUUG

>ptc-miR477a-5p MIMAT0002074

AUCUCCCUCAGAGGCUUCCAA

>ptc-miR477c MIMAT0025257

GGAAACCUUUUGUGGGGGUUUG

>ptc-miR477d-3p MIMAT0025276

UGGACUCCUUUGGGGAGAUGG

>ptc-miR477d-5p MIMAT0025275

AUCUCCCUCAAAGGCUUCCUCU

>ptc-miR482c-3p MIMAT0025278

UCUUUCCGAGUCCUCCCAUACC

>ptc-miR482c-5p MIMAT0025277

UAUGGGAGAGGCGGGAAUGACU

>ptc-miR6427-3p MIMAT0025228

GUGGGAAUGAACAUUAUGAGA

>ptc-miR6427-5p MIMAT0025227

UCGUAAUGCUUCAUUCUCACAA

>ptc-miR6445a MIMAT0025248

UUCAUUCCUCUUCCUAAAAUGG

>ptc-miR6459a-3p MIMAT0025185

UCGAAUUUGGGCUUGAGAUUG

>ptc-miR6459a-5p MIMAT0025184

AGCUCAAGCACAAAUUCGAUC

>ptc-miR6463 MIMAT0025189

UGGAUGAUCAUGUUGGCAACC

>ptc-miR6474 MIMAT0025213

UGUUCAGAUCAGUAGAUAGCA

>sbi-miR156e MIMAT0001758

UGACAGAAGAGAGCGAGCAC

>sbi-miR166a MIMAT0001394

UCGGACCAGGCUUCAUUCCC

>sbi-miR167e MIMAT0001450

UGAAGCUGCCAGCAUGAUCUG

>sbi-miR167i MIMAT0011332

UGAAGCUGCCAGCAUGAUCUA

>sbi-miR169d-3p MIMAT0026431

GGGCGGUCACCUUGGCUAGC

>sbi-miR169d-5p MIMAT0001454

UAGCCAAGGAUGACUUGCCU

>sbi-miR171h MIMAT0011341

GGAUUGAGCCGCGUCAAUAUC

>sbi-miR172a MIMAT0001397

AGAAUCUUGAUGAUGCUGCA

>sbi-miR396e MIMAT0011358

UUCCACAGGCUUUCUUGAACUG

>sbi-miR5564a MIMAT0022241

UGGGGAAGCAAUUCGUCGAACA

>sbi-miR5564b MIMAT0022242

GCAAUUCGUCGAACAGCUUGA

>sbi-miR6223-3p MIMAT0026413

CUAGCAUGUUCCUCCUAAGAG

>sbi-miR6223-5p MIMAT0026412

UUCUUGGGAGGAGCAUGCUAG

>sly-miR164a-3p MIMAT0033974

CAUGUGCCUGUUUUCCCCAUC

>sly-miR166c-3p MIMAT0035444

UCGGACCAGGCUUCAUUCCUC

>sly-miR166c-5p MIMAT0035443

GGGAUGUUGUCUGGCUCGACA

>sly-miR167a MIMAT0007917

UGAAGCUGCCAGCAUGAUCUA

>sly-miR167b-3p MIMAT0035458

AGGUCAUCUAGCAGCUUCAAU

>sly-miR167b-5p MIMAT0035457

UAAAGCUGCCAGCAUGAUCUGG

>sly-miR171a MIMAT0007922

UGAUUGAGCCGUGCCAAUAUC

>sly-miR172b MIMAT0009144

AGAAUCUUGAUGAUGCUGCAU

>sly-miR1919b MIMAT0007912

ACGAGAGUCAUCUGUGACAGG

>sly-miR1919c-3p MIMAT0007913

ACGAGAGUCAUCUGUGACAGG

>sly-miR1919c-5p MIMAT0032040

UGUCGCAGAUGACUUUCGCCC

>sly-miR319c-3p MIMAT0035432

UUGGACUGAAGGGAGCUCCUU

>sly-miR319c-5p MIMAT0035431

AGAGCUUCCUUCAGCCCACUC

>sly-miR390b-3p MIMAT0035480

CGCUAUCCAUCCUGAGUUUCA

>sly-miR394-3p MIMAT0035438

AGGUGGGCAUACUGUCAACA

>sly-miR397 MIMAT0007928

AUUGAGUGCAGCGUUGAUGA

>sly-miR399 MIMAT0009146

UGCCAAAGGAGAGUUGCCCUA

>sly-miR403-3p MIMAT0035434

CUAGAUUCACGCACAAGCUCG

>sly-miR403-5p MIMAT0035433

CGUUUGUGCGUGAAUCUAACA

>sly-miR4376 MIMAT0022688

ACGCAGGAGAGAUGAUGCUGGA

>sly-miR482a MIMAT0020769

UUUCCAAUUCCACCCAUUCCUA

>sly-miR482d-3p MIMAT0035460

UUUCCUAUUCCACCCAUGCCAA

>sly-miR482d-5p MIMAT0035459

GGAGUGGGUGGGAUGGAAAAA

>sly-miR482e-3p MIMAT0032124

UCUUUCCUACUCCUCCCAUACC

>sly-miR482e-5p MIMAT0020765

UGUGGGUGGGGUGGAAAGAUU

>sly-miR6026 MIMAT0023610

UUCUUGGCUAGAGUUGUAUUGC

>sly-miR6027-3p MIMAT0023611

UGAAUCCUUCGGCUAUCCAUAA

>sly-miR6027-5p MIMAT0032133

AUGGGUAGCACAAGGAUUAAUG

>sly-miR9470-3p MIMAT0035440

UUUGGCUCAUGGAUUUUAGC

>sly-miR9470-5p MIMAT0035439

UGAAAUCCAUGAGCCUAAACU

>sly-miR9472-3p MIMAT0035450

UUCACAAUCUCUGCUGAAAAA

>sly-miR9472-5p MIMAT0035449

UUUCAGUAGACGUUGUGAAUA

>sly-miR9475-3p MIMAT0035466

CUACAAUGUAGAGAUCGUUUU

>sly-miR9475-5p MIMAT0035465

AACGAUCUCUACAUUGUAGGC

>sly-miR9476-3p MIMAT0035470

AAAAAGAUGCAGGACUAGACC

>sly-miR9476-5p MIMAT0035469

UCUAGUCCUGCAUCUUUUUUU

>stu-miR482a-5p MIMAT0031161

GGAAUUGGUGGAUUGGAAAGC

>stu-miR482b-3p MIMAT0023597

UUACCGAUUCCCCCCAUUCCAA

>stu-miR482b-5p MIMAT0031160

GGAGUGGGUGGCAUGGUAAGA

>stu-miR482c MIMAT0023596

UUUCCUAUUCCACCCAUGCCAA

>stu-miR482d-3p MIMAT0023600

UCUUGCCUACACCGCCCAUGCC

>stu-miR482d-5p MIMAT0031162

CGUGAGUGGUGGGGUAAGAUA

>stu-miR482e-3p MIMAT0023601

UCUUGCCAAUACCGCCCAUUCC

>stu-miR482e-5p MIMAT0031163

AGUGGGUGGUGUGGUAAGAUU

>stu-miR6024-3p MIMAT0023593

UUUUAGCAAGAGUUGUUUUCCC

>stu-miR6024-5p MIMAT0031159

AGAAACAACACUUGCUAAAAGA

>stu-miR6025 MIMAT0023608

UACCAACAAUUGAGAUAACAUC

>stu-miR6026-5p MIMAT0031164

AAUACAACUAUUGCCAAGACAA

>tae-miR156 MIMAT0018208

UGACAGAAGAGAGUGAGCACA

>tae-miR167c-5p MIMAT0035789

UGAAGCUGCCAGCAUGAUCUGC

>tae-miR396-5p MIMAT0035771

AACUGUGAACUCGCGGGGAUG

>tae-miR397-5p MIMAT0035795

UCACCGGCGCUGCACACAAUG

>tae-miR5048-5p MIMAT0035803

UUUGCAGGUUUUAGGUCUAAGU

>tae-miR5062-5p MIMAT0035781

UGAACCUUAGGGAACAGCCGCAU

>tae-miR5384-3p MIMAT0035797

UGAGCGCGCCGCCGUCGAAUG

>tae-miR7757-5p MIMAT0035800

AUAAAACCUUCAGCUAUCCAUC

>tae-miR9656-3p MIMAT0035763

CUUCGAGACUCUGAACAGCGG

>tae-miR9662a-3p MIMAT0035772

UUGAACAUCCCAGAGCCACCG

>tae-miR9662b-3p MIMAT0035780

UGAACAUCCCAGAGCCACCGG

>tae-miR9663-5p MIMAT0035773

AAGCGUAGUCGAACGAAUCUG

>tae-miR9664-3p MIMAT0035775

UUGCAGUCCUCGAUGUCGUAG

>tae-miR9666b-3p MIMAT0035813

CGGUUGGGCUGUAUGAUGGCGA

>tae-miR9666b-5p MIMAT0035812

GCCAUCAUACGUCCAACCGUG

>tae-miR9669-5p MIMAT0035783

UACUGUGGGCACUUAUUUGAC

>tae-miR9670-3p MIMAT0035785

AGGUGGAAUACUUGAAGAAGA

>tae-miR9674a-5p MIMAT0035793

GCAUCAUCCAUCCUACCAUUC

>tae-miR9677a MIMAT0035804

UGGCCGUUGGUAGAGUAGGAGA

>tae-miR9772 MIMAT0036982

UGAGAUGAGAUUACCCCAUAC

>vvi-miR156b MIMAT0005641

UGACAGAAGAGAGUGAGCAC

>vvi-miR156d MIMAT0005643

UGACAGAAGAGAGUGAGCAC

>vvi-miR156g MIMAT0005646

UUGACAGAAGAUAGAGAGCAC

>vvi-miR160b MIMAT0005652

UGCCUGGCUCCCUGAAUGCCAUC

>vvi-miR162 MIMAT0005657

UCGAUAAACCUCUGCAUCCAG

>vvi-miR166a MIMAT0005662

UCGGACCAGGCUUCAUUCC

>vvi-miR168 MIMAT0005675

UCGCUUGGUGCAGGUCGGGAA

>vvi-miR169g MIMAT0005682

CAGCCAAGGAUGACUUGCCGA

>vvi-miR169u MIMAT0005690

UGAGUCAAGGAUGACUUGCCG

>vvi-miR171i MIMAT0005698

UGAUUGAGCCGUGCCAAUAUC

>vvi-miR172c MIMAT0005701

GGAAUCUUGAUGAUGCUGCAG

>vvi-miR2111-3p MIMAT0016365

GUCCUCUGGUUGCAGAUUACU

>vvi-miR2111-5p MIMAT0016364

UAAUCUGCAUCCUGAGGUCUA

>vvi-miR2950-3p MIMAT0018010

UGGUGUGCACGGGAUGGAAUA

>vvi-miR2950-5p MIMAT0018009

UUCCAUCUCUUGCACACUGGA

>vvi-miR3623-3p MIMAT0018008

UGGUGCUUGGACGAAUUUGCUA

>vvi-miR3623-5p MIMAT0018007

UCACAAGUUCAUCCAAGCACCA

>vvi-miR3624-3p MIMAT0018012

UCAGGGCAGCAGCAUACUACU

>vvi-miR3624-5p MIMAT0018011

UAGUAUGCUGCUGUCUUUAGA

>vvi-miR3633a-3p MIMAT0018038

UUCCUAUACCACCCAUUCCCUA

>vvi-miR3633a-5p MIMAT0018037

GGAAUGGAUGGUUAGGAGAG

>vvi-miR3634-3p MIMAT0018040

UUUCCGACUCGCACUCAUGCCGU

>vvi-miR3634-5p MIMAT0018039

GGCAUAUGUGUGACGGAAAGA

>vvi-miR390 MIMAT0005707

AAGCUCAGGAGGGAUAGCGCC

>vvi-miR396b MIMAT0005725

UUCCACAGCUUUCUUGAACU

>vvi-miR396c MIMAT0006560

UUCCACAGCUUUCUUGAACUG

>vvi-miR396d MIMAT0005726

UUCCACAGCUUUCUUGAACUG

>vvi-miR397a MIMAT0006561

UCAUUGAGUGCAGCGUUGAUG

>vvi-miR399a MIMAT0005728

UGCCAAAGGAGAAUUGCCCUG

>vvi-miR403b MIMAT0006570

UUAGAUUCACGCACAAACUCG

>vvi-miR408 MIMAT0005733

AUGCACUGCCUCUUCCCUGGC

>vvi-miR477b-3p MIMAT0018026

CGAAGUCUUUGGGGAGAGUGG

>vvi-miR477b-5p MIMAT0018025

ACUCUUUCUCAAGGGCUUCUAG

>vvi-miR479 MIMAT0005734

UGUGGUAUUGGUUCGGCUCAUC

>vvi-miR482 MIMAT0006576

UCUUUCCUACUCCUCCCAUUCC

>zma-miR156d-3p MIMAT0015124

GCUCACUUCUCUUUCUGUCAGC

>zma-miR156e-3p MIMAT0015128

GCUCACUGCUCUCUCUGUCAUC

>zma-miR156k-3p MIMAT0015208

GCUCGCUUCUCUUUCUGUCAGC

>zma-miR156k-5p MIMAT0001749

UGACAGAAGAGAGCGAGCAC

>zma-miR159a-5p MIMAT0015176

GAGCUCCUAUCAUUCCAAUGA

>zma-miR166a-5p MIMAT0015146

GGAAUGUUGUCUGGCUCGGGG

>zma-miR166c-3p MIMAT0001383

UCGGACCAGGCUUCAUUCCC

>zma-miR166c-5p MIMAT0015152

GGAAUGUUGUCUGGCUCGAGG

>zma-miR166e MIMAT0001378

UCGGACCAGGCUUCAUUCCC

>zma-miR166g-3p MIMAT0001381

UCGGACCAGGCUUCAUUCCC

>zma-miR166g-5p MIMAT0015150

GGAAUGUUGUCUGGUUGGAGA

>zma-miR166k-5p MIMAT0015184

GGAUUGUUGUCUGGCUCGGGG

>zma-miR166m-3p MIMAT0001744

UCGGACCAGGCUUCAUUCCUC

>zma-miR166m-5p MIMAT0015204

GGAAUGUUGGCUGGCUCGAGG

>zma-miR166n-3p MIMAT0013986

UCGGACCAGGCUUCAAUCCCU

>zma-miR166n-5p MIMAT0015321

GGAUUGUUGUCUGGCUCGGUG

>zma-miR167c-3p MIMAT0015145

GAUCAUGCUGUGGCAGCCUCACU

>zma-miR167e-3p MIMAT0015186

GAUCAUGCUGUGCAGUUUCAUC

>zma-miR167h-3p MIMAT0015189

GAUCAUGUUGCAGCUUCAC

>zma-miR167i-3p MIMAT0015190

GAUCAUGUUGCAGCUUCAC

>zma-miR167j-3p MIMAT0015322

GAUCAUGUGGCAGUUUCAUU

>zma-miR167j-5p MIMAT0013987

UGAAGCUGCCAGCAUGAUCUG

>zma-miR168a-3p MIMAT0015191

CCCGCCUUGCACCAAGUGAA

>zma-miR168a-5p MIMAT0001726

UCGCUUGGUGCAGAUCGGGAC

>zma-miR168b-3p MIMAT0015192

CCCGCCUUGCAUCAAGUGAA

>zma-miR168b-5p MIMAT0001727

UCGCUUGGUGCAGAUCGGGAC

>zma-miR169a-3p MIMAT0015140

GGCAAGUUGUUCUUGGCUACA

>zma-miR169b-3p MIMAT0015141

GGCAAGUUGUUCUUGGCUACA

>zma-miR169b-5p MIMAT0001369

CAGCCAAGGAUGACUUGCCGA

>zma-miR171d-5p MIMAT0015160

UGUUGGCUCGGCUCACUCAGA

>zma-miR172c-3p MIMAT0001390

AGAAUCUUGAUGAUGCUGCA

>zma-miR172c-5p MIMAT0015158

CAGCACCACCAAGAUUCACA

>zma-miR319a-3p MIMAT0001715

UUGGACUGAAGGGUGCUCCC

>zma-miR319a-5p MIMAT0015180

GAGCUCUCUUCAGUCCACUC

>zma-miR319b-3p MIMAT0001717

UUGGACUGAAGGGUGCUCCC

>zma-miR319b-5p MIMAT0015182

AGAGCGUCCUUCAGUCCACUC

>zma-miR319c-3p MIMAT0001716

UUGGACUGAAGGGUGCUCCC

>zma-miR319c-5p MIMAT0015181

GAGCUCUCUUCAGUCCACUC

>zma-miR390b-3p MIMAT0015367

CGCUAUCUAUCCUGAGCUCCA

>zma-miR396a-3p MIMAT0015168

GUUCAAUAAAGCUGUGGGAAA

>zma-miR396b-3p MIMAT0015167

GUUCAAUAAAGCUGUGGGAAA

>zma-miR396f-3p MIMAT0015350

GGUCAAGAAAGCUGUGGGAAG

>zma-miR396f-5p MIMAT0014015

UUCCACAGCUUUCUUGAACUU

>zma-miR397b-3p MIMAT0015353

CCAGCGCUGCACUCAAUUACG

>zma-miR397b-5p MIMAT0014019

UCAUUGAGCGCAGCGUUGAUG

>zma-miR399b-3p MIMAT0001706

UGCCAAAGGAGAGCUGUCCUG

>zma-miR399b-5p MIMAT0015171

GUGCAGCUCUCCUCUGGCAUG

>zma-miR399d-3p MIMAT0001707

UGCCAAAGGAGAGCUGCCCUG

>zma-miR399d-5p MIMAT0015172

GUGUGGCUCUCCUCUGGCAUG

>zma-miR399f-3p MIMAT0001709

UGCCAAAGGAAAUUUGCCCCG

>zma-miR399f-5p MIMAT0015174

GGGCAACUUCUCCUUUGGCAGA

>zma-miR399h-3p MIMAT0014023

UGCCAAAGGAGAAUUGCCCUG

>zma-miR399h-5p MIMAT0015357

GUGCAGUUCUCCUCUGGCACG

>zma-miR399j-3p MIMAT0014025

UGCCAAAGGAGAGUUGCCCUG

>zma-miR399j-5p MIMAT0015359

AGGCAGCUCUCCUCUGGCAGG

>zma-miR408a MIMAT0001748

CUGCACUGCCUCUUCCCUGGC

>zma-miR444a MIMAT0022261

UGCAGUUGUUGUCUCAAGCUU

>zma-miR529-3p MIMAT0015364

GCUGUACCCUCUCUCUUCUUC

>zma-miR529-5p MIMAT0014030

AGAAGAGAGAGAGUACAGCCU

>zma-miR827-3p MIMAT0014031

UUAGAUGACCAUCAGCAAACA

>zma-miR827-5p MIMAT0015365

UUUGUUGGUGGUCAUUUAACC

>ath-miR156a-5p MIMAT0000166

UGACAGAAGAGAGUGAGCAC

>ath-miR156b-5p MIMAT0000167

UGACAGAAGAGAGUGAGCAC

>ath-miR156c-5p MIMAT0000168

UGACAGAAGAGAGUGAGCAC

>ath-miR156d-5p MIMAT0000169

UGACAGAAGAGAGUGAGCAC

>ath-miR156f-5p MIMAT0000171

UGACAGAAGAGAGUGAGCAC

>ath-miR159a MIMAT0000177

UUUGGAUUGAAGGGAGCUCUA

>ath-miR160a-5p MIMAT0000178

UGCCUGGCUCCCUGUAUGCCA

>ath-miR161.1 MIMAT0000181

UGAAAGUGACUACAUCGGGGU

>ath-miR161.2 MIMAT0006779

UCAAUGCAUUGAAAGUGACUA

>ath-miR162a-3p MIMAT0000182

UCGAUAAACCUCUGCAUCCAG

>ath-miR166a-3p MIMAT0000189

UCGGACCAGGCUUCAUUCCCC

>ath-miR166b-3p MIMAT0000190

UCGGACCAGGCUUCAUUCCCC

>ath-miR166e-3p MIMAT0000193

UCGGACCAGGCUUCAUUCCCC

>ath-miR167a-5p MIMAT0000196

UGAAGCUGCCAGCAUGAUCUA

>ath-miR167d MIMAT0000905

UGAAGCUGCCAGCAUGAUCUGG

>ath-miR169b-5p MIMAT0000906

CAGCCAAGGAUGACUUGCCGG

>ath-miR169e MIMAT0000909

UGAGCCAAGGAUGACUUGCCG

>ath-miR169g-3p MIMAT0000912

UCCGGCAAGUUGACCUUGGCU

>ath-miR171a-3p MIMAT0000202

UGAUUGAGCCGCGCCAAUAUC

>ath-miR172b-3p MIMAT0000205

AGAAUCUUGAUGAUGCUGCAU

>ath-miR172c MIMAT0000922

AGAAUCUUGAUGAUGCUGCAG

>ath-miR319a MIMAT0000511

UUGGACUGAAGGGAGCUCCCU

>ath-miR319c MIMAT0001016

UUGGACUGAAGGGAGCUCCUU

>ath-miR390b-5p MIMAT0000932

AAGCUCAGGAGGGAUAGCGCC

>ath-miR395a MIMAT0000938

CUGAAGUGUUUGGGGGAACUC

>ath-miR395b MIMAT0000939

CUGAAGUGUUUGGGGGGACUC

>ath-miR395c MIMAT0000940

CUGAAGUGUUUGGGGGGACUC

>ath-miR395d MIMAT0000941

CUGAAGUGUUUGGGGGAACUC

>ath-miR395e MIMAT0000942

CUGAAGUGUUUGGGGGAACUC

>ath-miR395f MIMAT0000943

CUGAAGUGUUUGGGGGGACUC

>ath-miR396a-5p MIMAT0000944

UUCCACAGCUUUCUUGAACUG

>ath-miR396b-5p MIMAT0000945

UUCCACAGCUUUCUUGAACUU

>ath-miR397b MIMAT0000947

UCAUUGAGUGCAUCGUUGAUG

>ath-miR399a MIMAT0000951

UGCCAAAGGAGAUUUGCCCUG

>ath-miR399b MIMAT0000952

UGCCAAAGGAGAGUUGCCCUG

>ath-miR399d MIMAT0000954

UGCCAAAGGAGAUUUGCCCCG

>ath-miR399e MIMAT0000955

UGCCAAAGGAGAUUUGCCUCG

>ath-miR399f MIMAT0000956

UGCCAAAGGAGAUUUGCCCGG

>ath-miR401 MIMAT0001002

CGAAACUGGUGUCGACCGACA

>ath-miR402 MIMAT0001003

UUCGAGGCCUAUUAAACCUCUG

>ath-miR404 MIMAT0001005

AUUAACGCUGGCGGUUGCGGCAGC

>ath-miR405a MIMAT0001006

AUGAGUUGGGUCUAACCCAUAACU

>ath-miR405b MIMAT0001007

AUGAGUUGGGUCUAACCCAUAACU

>ath-miR405d MIMAT0001008

AUGAGUUGGGUCUAACCCAUAACU

>ath-miR406 MIMAT0001009

UAGAAUGCUAUUGUAAUCCAG

>ath-miR407 MIMAT0001010

UUUAAAUCAUAUACUUUUGGU

>ath-miR408-3p MIMAT0001011

AUGCACUGCCUCUUCCCUGGC

>ath-miR413 MIMAT0001321

AUAGUUUCUCUUGUUCUGCAC

>ath-miR414 MIMAT0001322

UCAUCUUCAUCAUCAUCGUCA

>ath-miR415 MIMAT0001323

AACAGAGCAGAAACAGAACAU

>ath-miR416 MIMAT0001324

GGUUCGUACGUACACUGUUCA

>ath-miR417 MIMAT0001325

GAAGGUAGUGAAUUUGUUCGA

>ath-miR418 MIMAT0001326

UAAUGUGAUGAUGAACUGACC

>ath-miR419 MIMAT0001327

UUAUGAAUGCUGAGGAUGUUG

>ath-miR420 MIMAT0001328

UAAACUAAUCACGGAAAUGCA

>ath-miR426 MIMAT0001337

UUUUGGAAAUUUGUCCUUACG

>ath-miR447a-3p MIMAT0002113

UUGGGGACGAGAUGUUUUGUUG

>ath-miR447b MIMAT0002114

UUGGGGACGAGAUGUUUUGUUG

>ath-miR447c-3p MIMAT0002115

UUGGGGACGACAUCUUUUGUUG

>ath-miR472-3p MIMAT0003931

UUUUUCCUACUCCGCCCAUACC

>ath-miR771 MIMAT0003930

UGAGCCUCUGUGGUAGCCCUCA

>ath-miR773a MIMAT0003932

UUUGCUUCCAGCUUUUGUCUC

>ath-miR774a MIMAT0003933

UUGGUUACCCAUAUGGCCAUC

>ath-miR775 MIMAT0003934

UUCGAUGUCUAGCAGUGCCA

>ath-miR776 MIMAT0003935

UCUAAGUCUUCUAUUGAUGUU

>ath-miR777 MIMAT0003936

UACGCAUUGAGUUUCGUUGCUU

>ath-miR778 MIMAT0003937

UGGCUUGGUUUAUGUACACCG

>ath-miR779.1 MIMAT0003938

UUCUGCUAUGUUGCUGCUCAU

>ath-miR780.1 MIMAT0004218

UCUAGCAGCUGUUGAGCAGGU

>ath-miR780.2 MIMAT0003939

UUCUUCGUGAAUAUCUGGCAU

>ath-miR781a MIMAT0003940

UUAGAGUUUUCUGGAUACUUA

>ath-miR782 MIMAT0003941

ACAAACACCUUGGAUGUUCUU

>ath-miR823 MIMAT0004240

UGGGUGGUGAUCAUAUAAGAU

>ath-miR825 MIMAT0004241

UUCUCAAGAAGGUGCAUGAAC

>ath-miR827 MIMAT0004243

UUAGAUGACCAUCAACAAACU

>ath-miR830-3p MIMAT0004248

UAACUAUUUUGAGAAGAAGUG

>ath-miR830-5p MIMAT0004247

UCUUCUCCAAAUAGUUUAGGUU

>ath-miR833a-3p MIMAT0004253

UAGACCGAUGUCAACAAACAAG

>ath-miR833a-5p MIMAT0004252

UGUUUGUUGUACUCGGUCUAGU

>ath-miR842 MIMAT0004264

UCAUGGUCAGAUCCGUCAUCC

>ath-miR843 MIMAT0004265

UUUAGGUCGAGCUUCAUUGGA

>ath-miR845a MIMAT0004268

CGGCUCUGAUACCAAUUGAUG

>ath-miR845b MIMAT0004317

UCGCUCUGAUACCAAAUUGAUG

>ath-miR852 MIMAT0004275

AAGAUAAGCGCCUUAGUUCUG

>ath-miR853 MIMAT0004276

UCCCCUCUUUAGCUUGGAGAAG

>ath-miR854a MIMAT0004280

GAUGAGGAUAGGGAGGAGGAG

>ath-miR854b MIMAT0004281

GAUGAGGAUAGGGAGGAGGAG

>ath-miR854c MIMAT0004282

GAUGAGGAUAGGGAGGAGGAG

>ath-miR854d MIMAT0004283

GAUGAGGAUAGGGAGGAGGAG

>ath-miR854e MIMAT0018504

GAUGAGGAUAGGGAGGAGGAG

>ath-miR855 MIMAT0004279

AGCAAAAGCUAAGGAAAAGGAA

>ath-miR856 MIMAT0004300

UAAUCCUACCAAUAACUUCAGC

>ath-miR857 MIMAT0004301

UUUUGUAUGUUGAAGGUGUAU

>ath-miR858a MIMAT0004302

UUUCGUUGUCUGUUCGACCUU

>ath-miR859 MIMAT0004303

UCUCUCUGUUGUGAAGUCAAA

>ath-miR860 MIMAT0004304

UCAAUAGAUUGGACUAUGUAU

>ath-miR861-5p MIMAT0004305

CCUUGGAGAAAUAUGCGUCAA

>ath-miR862-3p MIMAT0004308

AUAUGCUGGAUCUACUUGAAG

>ath-miR862-5p MIMAT0004307

UCCAAUAGGUCGAGCAUGUGC

>ath-miR863-3p MIMAT0004310

UUGAGAGCAACAAGACAUAAU

>ath-miR863-5p MIMAT0004309

UUAUGUCUUGUUGAUCUCAAU

>ath-miR865-3p MIMAT0004314

UUUUUCCUCAAAUUUAUCCAA

>ath-miR865-5p MIMAT0004313

AUGAAUUUGGAUCUAAUUGAG

>ath-miR866-3p MIMAT0004316

ACAAAAUCCGUCUUUGAAGA

>ath-miR866-5p MIMAT0004315

UCAAGGAACGGAUUUUGUUAA

>ath-miR867 MIMAT0004318

UUGAACAUGGUUUAUUAGGAA

>ath-miR868-3p MIMAT0004319

CUUCUUAAGUGCUGAUAAUGC

>ath-miR869.1 MIMAT0004320

AUUGGUUCAAUUCUGGUGUUG

>ath-miR869.2 MIMAT0004321

UCUGGUGUUGAGAUAGUUGAC

>ath-miR870-3p MIMAT0004322

UAAUUUGGUGUUUCUUCGAUC

>bcy-miR156 MIMAT0020964

UUUGACAGAAGAUAGAGAGCAC

>bcy-miR529 MIMAT0020967

GAAGAAGAGAGAUGGUAGAG

>bdi-miR156a MIMAT0012181

UGACAGAAGAGAGAGAGCACA

>bdi-miR444a MIMAT0012173

UUGCUGCCUCAAGCUUGCUGC

>bna-miR1140 MIMAT0005637

ACAGCCUAAACCAAUCGGAGC

>bna-miR156a MIMAT0004445

UGACAGAAGAGAGUGAGCACA

>bna-miR156b MIMAT0005636

UUGACAGAAGAUAGAGAGCAC

>bna-miR156c MIMAT0005639

UUGACAGAAGAUAGAGAGCAC

>bna-miR159 MIMAT0005635

UUUGGAUUGAAGGGAGCUCUA

>bna-miR161 MIMAT0005634

UCAAUGCACUGAAAGUGACUA

>bna-miR167a MIMAT0005626

UGAAGCUGCCAGCAUGAUCUAA

>bna-miR167b MIMAT0005627

UGAAGCUGCCAGCAUGAUCUAA

>bna-miR167c MIMAT0005628

UGAAGCUGCCAGCAUGAUCUA

>bna-miR168a MIMAT0005625

UCGCUUGGUGCAGGUCGGGAA

>bna-miR169a MIMAT0005612

CAGCCAAGGAUGACUUGCCGA

>bna-miR169b MIMAT0005613

CAGCCAAGGAUGACUUGCCGA

>bna-miR169c MIMAT0005614

UAGCCAAGGAUGACUUGCCUA

>bna-miR169d MIMAT0005615

UAGCCAAGGAUGACUUGCCUA

>bna-miR169e MIMAT0005616

UAGCCAAGGAUGACUUGCCUA

>bna-miR169f MIMAT0005617

UAGCCAAGGAUGACUUGCCUA

>bna-miR169g MIMAT0005618

UAGCCAAGGAUGACUUGCCUGC

>bna-miR169h MIMAT0005619

UAGCCAAGGAUGACUUGCCUGC

>bna-miR169i MIMAT0005620

UAGCCAAGGAUGACUUGCCUGC

>bna-miR169j MIMAT0005621

UAGCCAAGGAUGACUUGCCUGC

>bna-miR169k MIMAT0005622

UAGCCAAGGAUGACUUGCCUGC

>bna-miR169l MIMAT0005623

UAGCCAAGGAUGACUUGCCUGC

>bna-miR169m MIMAT0005624

UGAGCCAAAGAUGACUUGCCG

>bna-miR171a MIMAT0005605

UUGAGCCGUGCCAAUAUCACG

>bna-miR171b MIMAT0005606

UUGAGCCGUGCCAAUAUCACG

>bna-miR171c MIMAT0005607

UUGAGCCGUGCCAAUAUCACG

>bna-miR171d MIMAT0005608

UUGAGCCGUGCCAAUAUCACG

>bna-miR171e MIMAT0005609

UUGAGCCGUGCCAAUAUCACG

>bna-miR171f MIMAT0005610

UGAUUGAGCCGCGCCAAUAUC

>bna-miR171g MIMAT0004446

UGAUUGAGCCGCGCCAAUAUCU

>bna-miR390a MIMAT0005602

AAGCUCAGGAGGGAUAGCGCC

>bna-miR393 MIMAT0004447

UCCAAAGGGAUCGCAUUGAUC

>bna-miR396a MIMAT0004448

UUCCACAGCUUUCUUGAACUU

>bna-miR397a MIMAT0005600

UCAUUGAGUGCAGCGUUGAUGU

>bna-miR397b MIMAT0005601

UCAUUGAGUGCAGCGUUGAUGU

>bna-miR399a MIMAT0004449

UGCCAAAGGAGAUUUGCCCGG

>bna-miR824 MIMAT0005599

UAGACCAUUUGUGAGAAGGGA

>bra-miR157a MIMAT0010152

UUGACAGAAGAUAGAGAGCAC

>bra-miR167d MIMAT0010160

UGAAGCUGCCAGCAUGAUCUA

>bra-miR171e MIMAT0010165

UGAUUGAGCCGCGCCAAUAUC

>bra-miR172b-5p MIMAT0010167

GCAGCACCAUUAAGAUUCACA

>bra-miR1885a MIMAT0009213

CAUCAAUGAAAGGUAUGAUUCC

>ccl-miR167a MIMAT0014072

UGAAGCUGCCAGCAUGAUCUGA

>ccl-miR171 MIMAT0014075

UGAUUGAGCCGCGCCAAUAUC

>ccl-miR396 MIMAT0014078

UUCCACAGCUUUCUUGAACUU

>cme-miR164a MIMAT0022749

UGGAGAAGCAGGGCACGUGCU

>cme-miR168 MIMAT0022751

UCGCUUGGUGCAGGUCGGGA

>cre-miR1142 MIMAT0005376

AAGGUGUGGAUGCGGCAUGGG

>cre-miR1143-3p MIMAT0005378

UUAUUUGCCCGAAGGGGACGUCCU

>cre-miR1143-5p MIMAT0005377

AGGACGUCCCCUUACGGGA

>cre-miR1144a.1 MIMAT0005380

CAGGCAGCGCGGGGCUGCUGG

>cre-miR1144a.2 MIMAT0005379

UGGAACCGGGCACGCAGGAG

>cre-miR1144b MIMAT0005435

UGGGUAGUGUGGCGGCAGGCAG

>cre-miR1145.1 MIMAT0005382

UUGGGGCCCAGCAGGUCCUGG

>cre-miR1146 MIMAT0005383

AUGGGUCCGAUCGGGAAGCU

>cre-miR1147.1 MIMAT0005384

AGUGCCAGCGCGCUCUCGGCC

>cre-miR1147.2 MIMAT0005385

UCUCGGCCAAGUCUGGCAGA

>cre-miR1148.1 MIMAT0005386

CCAACGUGCAGGGGGACAUGG

>cre-miR1148.2 MIMAT0005387

UGGAGAUCCUCCUGUCCGGCU

>cre-miR1149.1 MIMAT0005388

UCGGACAACACCGACCCCCAGC

>cre-miR1149.2 MIMAT0005389

UGUUGUCUGACAUGGAGGGUC

>cre-miR1150.1 MIMAT0005392

AGAGGGACAUUGAUGCGCUGC

>cre-miR1150.2 MIMAT0005391

UCGCCGAGAGGGACAUUGAU

>cre-miR1150.3 MIMAT0005390

UGCAGCGGCGACUGGGGCCGA

>cre-miR1151a-3p MIMAT0005394

ACGGGGUGUGGGACCCGG

>cre-miR1151a-5p MIMAT0005393

UCCGGGGCUCAUAACCUGUUG

>cre-miR1153-3p.1 MIMAT0005401

GAUUGUAAUGCGAUGGCUC

>cre-miR1153-3p.2 MIMAT0005400

UUGUAAUUGCCGGUAGCACUGA

>cre-miR1153-5p.1 MIMAT0005398

UGGGCCAUCGUAUUACUAUCAG

>cre-miR1153-5p.2 MIMAT0005399

AGUGCUGCUAGCAACUACAAG

>cre-miR1156.1 MIMAT0005405

UGGACCCUCGCAUGUCCGUGA

>cre-miR1156.2 MIMAT0005406

UUCAGCUGGAGCUUCAGGCAC

>cre-miR1158 MIMAT0005409

ACUUGGAGGAGGCCACUGGC

>cre-miR1159.1 MIMAT0005437

UGCCACAGUGCCCGAUUGCCG

>cre-miR1159.2 MIMAT0005436

ACAAUGCCAAUGGAGACGGAU

>cre-miR1160.1 MIMAT0005411

UUGCCCUUUUUAAGCAGGGC

>cre-miR1160.2 MIMAT0005410

UGACAAGGAAGCAGAGCGGAU

>cre-miR1160.3 MIMAT0005412

AAGGACCCGUGCGGGAAGGGA

>cre-miR1161a MIMAT0005413

UACUGGAGUUCUCAACAGC

>cre-miR1161b MIMAT0018505

UACUGGAGUUCUCAACAGC

>cre-miR1163.1 MIMAT0005418

AAGAGCGCCAUGGCACGCAGC

>cre-miR1163.2 MIMAT0005417

AGGGCAUGCUGCGUGCCAUGG

>cre-miR1164 MIMAT0005419

UGGUGCAACAGGCCAGUGGUU

>cre-miR1166.1 MIMAT0005422

UGGACCUCGCGGCCCUGGAGG

>cre-miR1166.2 MIMAT0005423

AGGUCCAUGACCUCAUGGG

>cre-miR1167 MIMAT0005434

GGGGUGUGAUGAUUUGAAAC

>cre-miR1168.1 MIMAT0005424

UGUGGACAAGGCCAAGUCCGA

>cre-miR1168.2 MIMAT0005425

AGCACGGAAGGCGAAGA

>cre-miR1169-3p MIMAT0005427

UGUGGAUGUUGCUUGCUGGAU

>cre-miR1169-5p MIMAT0005426

UAUCCAGCAAGCAACAUCCACA

>cre-miR1170.1 MIMAT0005429

UGUCCAUCGCCAAGUUGCCAG

>cre-miR1170.2 MIMAT0005428

AAUCAGCCAAACACGGCAGA

>cre-miR1171 MIMAT0005430

UGGAGUGGAGUGGAGUGGAGUGG

>cre-miR1172.1 MIMAT0005433

AGGAUUGCAGCAGCAACGGGGC

>cre-miR1172.2 MIMAT0005432

UAGGAUCGGAGACGCAGUGAA

>cre-miR1173 MIMAT0005431

AUGGUUGCAAUAGAAAUCAUG

>cre-miR905-3p MIMAT0004386

AAGGUGCCAUAUCCAGGGACC

>cre-miR905-5p MIMAT0004964

AGGUCCCUGGAUAUGGCACC

>cre-miR906-3p MIMAT0004388

UCCGAUAAAGCUUCCCCCUGC

>cre-miR906-5p MIMAT0004387

CGGUUGGUGGGCGUGAUCAGC

>cre-miR907 MIMAT0004389

UCUUCUGCGAGCGGUGCGAGC

>cre-miR908.1 MIMAT0004965

UGAGAAGAUGCGGUCCGUUGGC

>cre-miR908.2 MIMAT0004390

UGACGCGUUUGAUAGCAGGAUC

>cre-miR908.3 MIMAT0004966

ACUACGUCAAUAAGGCAGC

>cre-miR909.1 MIMAT0004967

UGCUGGUCAAACCGGUGGUGG

>cre-miR909.2 MIMAT0004391

AUGCAAACAUGACCCUGAAUG

>cre-miR909.3 MIMAT0004392

UUCAGGGUCAAGUUUGCAUGC

>cre-miR910 MIMAT0004393

AGCAGCGUCGGGCUCGACCGC

>cre-miR912 MIMAT0004395

UGGAUUGAUCCCAGCCAGGC

>cre-miR913-5p MIMAT0004968

UGCACACUUGCGAGUCCGUGG

>cre-miR914 MIMAT0004397

CGGGCGCGCCGGAAUCCGUGG

>cre-miR915 MIMAT0004398

UGGCAAUAAGGCAAUCGUUGC

>cre-miR917 MIMAT0004404

UUUCACGGUUAUGUUCGAAG

>cre-miR918 MIMAT0004385

UACCUGAAGCGGACAUCUUGC

>cre-miR919.1 MIMAT0004969

AAUCGAGAUGCUGACCGAGAU

>cre-miR919.2 MIMAT0004399

UCUCAGGAGGACAUCGCCACU

>csi-miR169 MIMAT0014074

GAGCCAAGAAUGACUUGCCGA

>csi-miR172a-3p MIMAT0014076

AGAAUCUUGAUGAUGCUGCA

>csi-miR398 MIMAT0014079

UGUGUUCUCAGGUCACCCCUU

>ctr-miR166 MIMAT0014071

UCGGACCAGGCUUCAUUCCCCC

>ctr-miR319 MIMAT0014077

UUGGACUGAAGGGAGCUCCC

>egu-miR172d MIMAT0024023

UGAAUCUUGAUGAUGCUGCAC

>egu-miR172e MIMAT0024024

UGAAUCUUGAUGAUGCUGCAC

>egu-miR172f MIMAT0024025

UGAAUCUUGAUGAUGCUGCAC

>gma-miR1507a MIMAT0007365

UCUCAUUCCAUACAUCGUCUGA

>gma-miR1507b MIMAT0010080

UCUCAUUCCAUACAUCGUCUG

>gma-miR1508a MIMAT0007366

UCUAGAAAGGGAAAUAGCAGUUG

>gma-miR1508b MIMAT0010081

UAGAAAGGGGAAUAGCAGUUG

>gma-miR1509a MIMAT0007367

UUAAUCAAGGAAAUCACGGUCG

>gma-miR1509b MIMAT0011201

UUAAUCAAGGAAAUCACGGUU

>gma-miR1510a-3p MIMAT0007368

UUGUUGUUUUACCUAUUCCACCC

>gma-miR1510b-3p MIMAT0010082

UGUUGUUUUACCUAUUCCACC

>gma-miR1512c MIMAT0022458

UAACUGAACAUUCUUAGAGCAU

>gma-miR1513a-5p MIMAT0007371

UGAGAGAAAGCCAUGACUUAC

>gma-miR159c MIMAT0007352

AUUGGAGUGAAGGGAGCUCCG

>gma-miR167e MIMAT0011196

UGAAGCUGCCAGCAUGAUCUU

>gma-miR167f MIMAT0011197

UGAAGCUGCCAGCAUGAUCUU

>gma-miR169a MIMAT0001693

CAGCCAAGGAUGACUUGCCGG

>gma-miR171b-3p MIMAT0007363

CGAGCCGAAUCAAUAUCACUC

>gma-miR172d MIMAT0011199

GGAAUCUUGAUGAUGCUGCAGCAG

>gma-miR172e MIMAT0011200

GGAAUCUUGAUGAUGCUGCAGCAG

>gma-miR2107 MIMAT0010083

CAAACCUCCGUAGCCUGUAUC

>gma-miR2108a MIMAT0010084

UUAAUGUGUUGUGUUUGUCGG

>gma-miR2108b MIMAT0010085

UUAAUGUGUUGUGUUUGUGAG

>gma-miR2109-5p MIMAT0010086

UGCGAGUGUCUUCGCCUCUG

>gma-miR2111a MIMAT0022457

GUCCUUGGGAUGCAGAUUACG

>gma-miR4387e MIMAT0022464

UGUUAGUGAUAAGGCGUGAUG

>gma-miR4401b MIMAT0022461

UCAAAGACGUUGCUGAGGUAA

>gma-miR5037d MIMAT0022455

CGGGAGCCUAUGAAGGUUAAC

>gma-miR530b MIMAT0022463

UGCAUUUGCACCUGCACUUUA

>gma-miR5667-3p MIMAT0022449

AAACAGAUCUAAAUGGAUUCC

>gma-miR5668 MIMAT0022450

AGCAAUGGAAUUAUAGACUGC

>gma-miR5669 MIMAT0022451

CAAUGUAGUGUGGUAAGUGGUC

>gma-miR5670a MIMAT0022452

CAUCAUACCAUAUUUGCUUCAU

>gma-miR5671a MIMAT0022453

CAUGGAAGUGAAUCGGGUGAC

>gma-miR5672 MIMAT0022454

CAUGGUAGUGGAAGAAAUGGA

>gma-miR5673 MIMAT0022456

CGUGGAAUCUCGCGGAAGACAU

>gma-miR5674a MIMAT0022459

UAAUUGUGUUGUACAUUAUCA

>gma-miR5675 MIMAT0022460

UAGAGACGACAACAAUGGAAA

>gma-miR5676 MIMAT0022462

UCGACACCAUAUGUAGAGGCAG

>gma-miR5677 MIMAT0022465

UUUGGUCUUUAAUCAAGCUGA

>gma-miR5678 MIMAT0022466

UUCCAUGAUAAGAUCUUUGAC

>gma-miR5679 MIMAT0022467

UUGGUGACCCAGAAGAAGUUGA

>gso-miR1507b MIMAT0016358

UCUCAUUCCAUACAUCGUCUGA

>gso-miR1508a MIMAT0016352

UAGAAAGGGAAAUAGCAGUUG

>gso-miR1510a MIMAT0016356

UGUUGUUUUACCUAUUCCACC

>gso-miR1510b MIMAT0016355

UGUUGUUUUACCUAUUCCACC

>gso-miR2109 MIMAT0016363

UGCGAGUGUCUUCGCCUCUGA

>gso-miR2218 MIMAT0016361

UUGCCGAUUCCACCCAUUCCUA

>gso-miR3522a MIMAT0016359

UGAGACCAAAUGAGCAGCUGA

>gso-miR3522b MIMAT0016360

UGAGACCAAAUGAGCAGCUGAC

>gso-miR482a MIMAT0016353

UCUUCCCUACACCUCCCAUAC

>gso-miR482b MIMAT0016354

UCUUCCCUACACCUCCCAUAC

>hvu-miR171-3p MIMAT0018220

UGAUUGAGCCGUGCCAAUAUC

>hvu-miR397b-3p MIMAT0035814

AUCAACGCUGCACUCAACGGC

>lja-miR1507a MIMAT0029330

UCUUCCAUCCAUACAUCAUCU

>lja-miR1507b MIMAT0029331

UCUUCCAUCCAUACAUCAUCU

>lja-miR167a MIMAT0029311

UGAAGCUGCCAGCAUGAUCU

>lja-miR167b MIMAT0029312

UGAAGCUGCCAGCAUGAUCU

>lja-miR167c MIMAT0029313

UGAAGCUGCCAGCAUGAUCU

>lja-miR171b MIMAT0029315

UGAUUGAGCCGCGUCAAUAUC

>lja-miR171c MIMAT0029316

UGAGCCGAAUCAAUAUCACUC

>lja-miR171d-3p MIMAT0029317

CGAUGUUGGUGAGGUUCAAUC

>lja-miR171d-5p MIMAT0029318

UUGAGCCGCGCCAAUAUCACU

>lja-miR172b MIMAT0029320

AGAAUCUUGAUGAUGCUGCA

>lja-miR172c MIMAT0029321

AGAAUCUUGAUGAUGCUGCA

>lja-miR397 MIMAT0029326

UAUUGAGUGCAGCGUUGAUGA

>lja-miR408 MIMAT0029327

CAGGGAAGAGGCAGAGCAUGG

>lja-miR7516-3p MIMAT0029329

AGAGACGUGACUCCCGCUACG

>lja-miR7516-5p MIMAT0029328

UAGCGGGUGUCUUCGCCUCUGA

>lja-miR7517 MIMAT0029332

AUAUGGUAAAGGUUAGGGACC

>lja-miR7518 MIMAT0029333

UUGCGCACUGAGCAAGGACAGG

>lja-miR7519 MIMAT0029334

CAAAUUUUCUAAGUGGGCUAGC

>lja-miR7520 MIMAT0029335

GAGGGGGAAGGUGAUGACAUC

>lja-miR7521 MIMAT0029336

UCAUGGGUGGGGUGUUAAACC

>lja-miR7522 MIMAT0029337

AACUGCGGACAGGUUUUAUGAC

>lja-miR7523a MIMAT0029338

ACCACCGGGCUCGAGGAUCAGC

>lja-miR7523b MIMAT0029339

ACCACCGGGCUCGAGGAUCAGC

>lja-miR7524 MIMAT0029340

ACCAGUGAGUCAUUGGGCGGA

>lja-miR7525 MIMAT0029341

AGGGCGUUUUGGUACAUGACU

>lja-miR7526a MIMAT0029342

AUCAAGGUAGCUGUAACUUCC

>lja-miR7526b MIMAT0029343

AUCAAGGUAGCUGUAACUUCC

>lja-miR7526c MIMAT0029344

AUCAAGGUAGCUGUAACUUCC

>lja-miR7526d MIMAT0029345

AUCAAGGUAGCUGUAACUUCC

>lja-miR7526e MIMAT0029346

AUCAAGGUAGCUGUAACUUCC

>lja-miR7526f MIMAT0029347

AUCAAGGUAGCUGUAACUUCC

>lja-miR7526g MIMAT0029348

AUCAAGGUAGCUGUAACUUCC

>lja-miR7526h MIMAT0029349

AUCAAGGUAGCUGUAACUUCC

>lja-miR7527 MIMAT0029350

CAUGGCGUGCAAAACCCCACGC

>lja-miR7528 MIMAT0029351

CCGAAAUGCUAAUCUGAAGCUU

>lja-miR7529 MIMAT0029352

CCGUAGCAUCAAUUUAUCCGA

>lja-miR7530 MIMAT0029353

CCUUCCUCUCUUCACUAUCUUC

>lja-miR7531 MIMAT0029354

CGUGUUUUCUUUCAUUCCCCA

>lja-miR7532a MIMAT0029355

GAAGCUGCCUCUGGUCGUGGU

>lja-miR7532b MIMAT0029356

GAAGCUGCCUCUGGUCGUGGU

>lja-miR7533a MIMAT0029357

GAGGGGAUGGAGAGAAGCUGG

>lja-miR7533b MIMAT0029358

GAGGGGAUGGAGAGAAGCUGG

>lja-miR7534 MIMAT0029359

GCAACUUGACUACAGUUUGAC

>lja-miR7535 MIMAT0029360

GGGAAAAUGUGGGUGUGGU

>lja-miR7536a MIMAT0029361

UAAGACAUGCUCAAGAGUG

>lja-miR7536b MIMAT0029362

UAAGACAUGCUCAAGAGUG

>lja-miR7537 MIMAT0029363

UAGGAAAUACGCCUGCGGUUCC

>lja-miR7538 MIMAT0029364

UCAACGGAGAGCUUGCUGUC

>lja-miR7539 MIMAT0029365

UCGAGAGAGAGAGCGACGAGG

>lja-miR7540a MIMAT0029366

UGAUAUGAUAAGUGAUGUGA

>lja-miR7540b MIMAT0029367

UGAUAUGAUAAGUGAUGUGA

>lja-miR7541 MIMAT0029368

UGCAUUCUCUUUUGGUGGCCC

>lja-miR7542 MIMAT0029369

UGCUUGCUUAUAGAUGGUG

>lja-miR7543 MIMAT0029370

UUAAUGAUACAUGUUUGACU

>lja-miR7544 MIMAT0029371

UUAGAAAGAAAAUGUUGUUAGC

>lja-miR7545 MIMAT0029372

UUGGGAAAGCUAGAGUGCU

>lja-miR7546 MIMAT0029373

UUGGUGACCGACAGGCGCGUGC

>lus-miR159c MIMAT0027235

UUUGGAUUGAAGGGAGCUCUU

>lus-miR162b MIMAT0027187

UCGAUAAACCUCUGCAUCCAG

>lus-miR166b MIMAT0027172

UCGGACCAGGCUUCAUCCCCC

>lus-miR167a MIMAT0027124

UGAAGCUGCCAGCAUGAUCUC

>lus-miR167h MIMAT0027177

UGAAGCUGCCAGCAUGAUCUA

>lus-miR168b MIMAT0027206

UCGCUUGGUGCAGGUCGGGAA

>lus-miR172e MIMAT0027174

GGAAUCUUGAUGAUGCUGCAG

>lus-miR172i MIMAT0027230

GGAAUCUUGAUGAUGCUGCAG

>lus-miR408a MIMAT0027210

AUGCACUGCCUCUUCCCUGGC

>mtr-miR1507-3p MIMAT0010031

CCUCGUUCCAUACAUCAUCUAG

>mtr-miR1507-5p MIMAT0010030

AGAGUUGUAUGGAACGAAAGAU

>mtr-miR1509a-3p MIMAT0010035

ACCGGAUUUCCUUGAUUAAAG

>mtr-miR1509a-5p MIMAT0010034

UUAAUCUAGGAAAAUACGGUG

>mtr-miR1510a-3p MIMAT0010033

CGGAGGAUUAGGUAAAACAAC

>mtr-miR1510a-5p MIMAT0010032

UUGUCUUACCCAUUCCUCCCA

>mtr-miR1510b-3p MIMAT0010029

ACAUGGUCGGUAUCCCUGGAA

>mtr-miR1510b-5p MIMAT0010028

CCAUGGAUCCCUACCAUGUGG

>mtr-miR156e MIMAT0011081

UUGACAGAAGAUAGAGAGCAC

>mtr-miR156f MIMAT0011091

UUGACAGAAGAUAGAGAGCAC

>mtr-miR156h-5p MIMAT0011099

UUGACAGAAGAUAGAGAGCAC

>mtr-miR160d MIMAT0011101

UGCCUGGCUCCCUGUAUGCCA

>mtr-miR164b MIMAT0011098

UGGAGAAGCAGGGCACGUGCA

>mtr-miR164c MIMAT0011102

UGGAGAAGCAGGGCACGUGCA

>mtr-miR168a MIMAT0011089

UUGCUUGGUGCUGGUCGGGAA

>mtr-miR171d MIMAT0011105

UGAUUGAGCCGUGCCAAUAUC

>mtr-miR172a MIMAT0011086

AGAAUCCUGAUGAUGCUGCAG

>mtr-miR2086-3p MIMAT0010027

GACAUGAAUGCAGAACUGGAA

>mtr-miR2086-5p MIMAT0010026

CCAGUUCUGCGUUCAUGUCCC

>mtr-miR2087-3p MIMAT0010037

CUGCAGUCGGUUUCUUACUUC

>mtr-miR2087-5p MIMAT0010036

GAAGUAAAGAACCGGCUGCAG

>mtr-miR2088-3p MIMAT0010039

UCCAAUGUAAUCUAGGUCUA

>mtr-miR2088-5p MIMAT0010038

AGGCCUAGAUUACAUUGGAC

>mtr-miR2089-3p MIMAT0010041

AGGAUUGGUGUAAUAGGUAAAA

>mtr-miR2089-5p MIMAT0010040

UUACCUAUUCCACCAAUUCCAU

>mtr-miR2118 MIMAT0011318

UUACCGAUUCCACCCAUUCCUA

>mtr-miR2119 MIMAT0011168

UCAAAGGGAGGUGUGGAGUAG

>mtr-miR2199 MIMAT0011319

UGAUACACUAGCACGGAUCAC

>mtr-miR395a MIMAT0001648

AUGAAGUGUUUGGGGGAACUC

>mtr-miR399a MIMAT0001651

UGCCAAAGGAGAUUUGCCCAG

>osa-miR1423-3p MIMAT0005957

AGCGCCCAAGCGGUAGUUGUC

>osa-miR1424 MIMAT0005958

AUGCACACUGAUGCUGAUUGU

>osa-miR1426 MIMAT0005960

AGAAUCUUGAUGAUGAUUAAA

>osa-miR1427 MIMAT0005961

UGCGGAACCGUGCGGUGGCGC

>osa-miR1428a-5p MIMAT0005962

CGUUUUGCAAAUUCGCAGGCC

>osa-miR1428e-3p MIMAT0007785

UAAGAUAAUGCCAUGAAUUUG

>osa-miR1429-3p MIMAT0005963

GUUGCACGGGUUUGUAUGUUG

>osa-miR1431 MIMAT0005965

UUUGCGAGUUGGCCCGCUUGC

>osa-miR1435 MIMAT0005986

UUUCUUAAGUCAAACUUUUU

>osa-miR1436 MIMAT0005987

ACAUUAUGGGACGGAGGGAGU

>osa-miR1437a MIMAT0005988

UCCGGCGCCGCACUAGGCACUG

>osa-miR1438 MIMAT0005989

AGGGUAAUUUUAUCAUUUUUAA

>osa-miR1439 MIMAT0005993

UUUUGGAACGGAGUGAGUAUU

>osa-miR1440a MIMAT0005990

UGCUCAAAUACCACUCUCCU

>osa-miR1441 MIMAT0005991

ACCGGAUGUCGGAAAAGGUUU

>osa-miR1442 MIMAT0005992

AUUCAUAGUACUAGAUGUGU

>osa-miR1874-3p MIMAT0007828

UAUGGAUGGAGGUGUAACCCGAUG

>osa-miR2055 MIMAT0009980

UUUCCUUGGGAAGGUGGUUUC

>osa-miR2120 MIMAT0011179

AAAGAUCUUUAGUCCCGGUUGUUC

>osa-miR2121a MIMAT0011180

AAAACGGAGCGGUCCAUUAGCGCG

>osa-miR2121b MIMAT0011181

AAAACGGAGCGGUCCAUUAGCGCG

>osa-miR2122 MIMAT0011182

UUUCAAAAAUAACCUUUUGUUC

>osa-miR2907a MIMAT0014036

GGCAGCCGAGCGAGGGCCUCGG

>osa-miR2907b MIMAT0014037

GGCAGCCGAGCGAGGGCCUCGG

>osa-miR2907c MIMAT0014038

GGCAGCCGAGCGAGGGCCUCGG

>osa-miR2907d MIMAT0014039

GGCAGCCGAGCGAGGGCCUCGG

>osa-miR2918 MIMAT0014049

AUCCGUGUUGUCUGCGCUUUA

>osa-miR2919 MIMAT0014050

AAGGGGGGGGGGGAAAGA

>osa-miR2920 MIMAT0014051

AAACAACAAUAUAACAUUUCAAA

>osa-miR2921 MIMAT0014052

AAGAACUUAAUAUAACUUUAAAGC

>osa-miR2922 MIMAT0014053

AAUAAGUGAUUACCGAAAUU

>osa-miR2923 MIMAT0014054

AGACAAAAAUAUAAAUAACAAA

>osa-miR2924 MIMAT0014055

CUCGCUUGCUCCGGCCGCCAC

>osa-miR2925 MIMAT0014056

UGGCGGCCGCGGGCUUCGU

>osa-miR2926 MIMAT0014057

AGGUCGUCGACGUUGGUGCU

>osa-miR2927 MIMAT0014058

UGUCGUCGUCGAUGGAGCCCAUG

>osa-miR2928 MIMAT0014059

AAGAAGACGACAUUUUGUUG

>osa-miR2929 MIMAT0014060

CUCAAGGGUGUUUGUGAAAUA

>osa-miR2930 MIMAT0014061

UUCUCUUCUCUCGCGCGUGGCC

>osa-miR2931 MIMAT0014062

CUUUAUUGUUGAUGUCAAAA

>osa-miR2932 MIMAT0014063

AGUAUGCCCACUACCUAUC

>osa-miR395w MIMAT0003877

GUGAAGUGUUUGGGGGAUUCUC

>osa-miR396e-5p MIMAT0001601

UCCACAGGCUUUCUUGAACUG

>osa-miR435 MIMAT0001585

UUAUCCGGUAUUGGAGUUGA

>osa-miR437 MIMAT0001586

AAAGUUAGAGAAGUUUGACUU

>osa-miR438 MIMAT0001587

UUCCCACGCGUUAUAGUGAAA

>osa-miR439a MIMAT0001589

UGUCGAACCGCGGUUGUUCGA

>osa-miR439b MIMAT0001590

UGUCGAACCGCGGUUGUUCGA

>osa-miR439c MIMAT0001591

UGUCGAACCGCGGUUGUUCGA

>osa-miR439d MIMAT0001592

UGUCGAACCGCGGUUGUUCGA

>osa-miR439e MIMAT0001593

UGUCGAACCGCGGUUGUUCGA

>osa-miR439f MIMAT0001594

UGUCGAACCGCGGUUGUUCGA

>osa-miR439g MIMAT0001595

UGUCGAACCGCGGUUGUUCGA

>osa-miR439h MIMAT0001596

UGUCGAACCGCGGUUGUUCGA

>osa-miR439i MIMAT0001598

UGUCGAACCGCGGUUGUUCGA

>osa-miR440 MIMAT0001599

AGUGUCUCCUGAUGAUCGGGACAA

>osa-miR443 MIMAT0001606

AUCACAAUACAAUAAAUCUGGA

>osa-miR444a-3p.1 MIMAT0001617

UUGCUGCCUCAAGCUUGCUGC

>osa-miR444a-3p.2 MIMAT0005790

UGCAGUUGCUGCCUCAAGCUU

>osa-miR444b.1 MIMAT0005969

UGUUGUCUCAAGCUUGCUGCC

>osa-miR444b.2 MIMAT0005968

UGCAGUUGUUGUCUCAAGCUU

>osa-miR444c.1 MIMAT0005971

UGUUGUCUCAAGCUUGCUGCC

>osa-miR444c.2 MIMAT0005970

UGCAGUUGUUGUCUCAAGCUU

>osa-miR444d.1 MIMAT0005974

UUGCUGCCUCAAGCUUGCUGC

>osa-miR444d.2 MIMAT0005973

UGCAGUUGCUGCCUCAAGCUU

>osa-miR444d.3 MIMAT0005972

UUGUGGCUUUCUUGCAAGUUG

>osa-miR444e MIMAT0005975

UGCAGUUGCUGCCUCAAGCUU

>osa-miR529a MIMAT0002885

CUGUACCCUCUCUCUUCUUC

>osa-miR531a MIMAT0002887

CUCGCCGGGGCUGCGUGCCGCCAU

>osa-miR531c MIMAT0031379

CUCGCCGGGGCUGCGUGCCGCCAU

>osa-miR810a MIMAT0004045

UCAUAAGCCCACCACAUGUGG

>osa-miR810b.1 MIMAT0005977

UGAACACCGAUAUGCGUCAUC

>osa-miR810b.2 MIMAT0005978

AAGUGAUUUAAUUAUGCCGUU

>osa-miR812a MIMAT0004049

GACGGACGGUUAAACGUUGGAC

>osa-miR812b MIMAT0004050

GACGGACGGUUAAACGUUGGAC

>osa-miR812c MIMAT0004051

GACGGACGGUUAAACGUUGGAC

>osa-miR812d MIMAT0004052

GACGGACGGUUAAACGUUGGAC

>osa-miR812e MIMAT0004053

GACGGACGGUUAAACGUUGGAC

>osa-miR814a MIMAT0004055

CACUUCAUAGUACAACGAAUCU

>osa-miR814b MIMAT0004056

CACUUCAUAGUACAACGAAUCU

>osa-miR814c MIMAT0004057

CACUUCAUAGUACAACGAAUCU

>osa-miR815a MIMAT0004058

AAGGGGAUUGAGGAGAUUGGG

>osa-miR815b MIMAT0004059

AAGGGGAUUGAGGAGAUUGGG

>osa-miR815c MIMAT0004060

AAGGGGAUUGAGGAGAUUGGG

>osa-miR816 MIMAT0004061

GUGACAUAUUUUACUACAAC

>osa-miR817 MIMAT0004062

UCCAACUUGAGGCCCGAUUGA

>osa-miR818a MIMAT0004063

AAUCCCUUAUAUUAUGGGACGG

>osa-miR818b MIMAT0004064

AAUCCCUUAUAUUAUGGGACGG

>osa-miR818c MIMAT0004065

AAUCCCUUAUAUUAUGGGACGG

>osa-miR818d MIMAT0004066

AAUCCCUUAUAUUAUGGGACGG

>osa-miR818e MIMAT0004067

AAUCCCUUAUAUUAUGGGACGG

>osa-miR820a MIMAT0004079

UCGGCCUCGUGGAUGGACCAG

>osa-miR820b MIMAT0004080

UCGGCCUCGUGGAUGGACCAG

>osa-miR820c MIMAT0004081

UCGGCCUCGUGGAUGGACCAG

>osa-miR821a MIMAT0004082

AAGUCAUCAACAAAAAAGUUGAAU

>osa-miR821b MIMAT0004083

AAGUCAUCAACAAAAAAGUUGAAU

>osa-miR821c MIMAT0004084

AAGUCAUCAACAAAAAAGUUGAAU

>osa-miR827 MIMAT0009981

UUAGAUGACCAUCAGCAAACA

>pab-miR1311 MIMAT0018162

UCAGAGUUUUGCCAGUUCCGCC

>pab-miR166a MIMAT0018148

UCGGACCAGGCUUCAUUCCUC

>pab-miR166b MIMAT0018149

UCGGACCAGGCUUCAUUCCUU

>pab-miR1863 MIMAT0018163

AGCUCUGAUACCAUGUUAGAUU

>pab-miR3693 MIMAT0018125

AGAGGGUGCUCAUGAACUGCUC

>pab-miR3694 MIMAT0018126

AUUAAGGGUGCGGGUGCGGCU

>pab-miR3696 MIMAT0018128

CGAGCUACCAUCGUGACGAUC

>pab-miR3698 MIMAT0018130

CUUGCAACUCUGCCUUGGCUUA

>pab-miR3699 MIMAT0018131

GACAGAAGAUAGACUUUGGUC

>pab-miR3700 MIMAT0018132

GACGCCCAAAACUGAAGGUCA

>pab-miR3701 MIMAT0018133

UAAACAGUGCCCACCCUUCAUC

>pab-miR3702 MIMAT0018134

AAUCUCUUGGUGCUUAUUCGC

>pab-miR3703 MIMAT0018135

UAGACUCUAUCAGCCUUGUCC

>pab-miR3704 MIMAT0018136

GGUCUAGGUGGAGUUGGAAAAA

>pab-miR3705 MIMAT0018137

GUAAGUGGUUAUGAUCUGGAC

>pab-miR3706 MIMAT0018138

UUUCGGAGAAAUGGAUAAGA

>pab-miR3707 MIMAT0018139

AUGAUCUGCCAUUAUCCUUGA

>pab-miR3708 MIMAT0018140

UCACACAACAUUUCUCGUACA

>pab-miR3709a MIMAT0018141

UCAGAUGCUUUAAAUUCCCGA

>pab-miR3709b MIMAT0018142

UUUAAAUGCCUUAAAUUCCCGA

>pab-miR3710 MIMAT0018143

UGGGAACCUGACGGGCCUCCA

>pab-miR3711 MIMAT0018144

UGGCGCUAGAAGGAGGGCCU

>pab-miR3712 MIMAT0018145

UGUGAUCAAGAUCAGACUACCA

>pab-miR395 MIMAT0018151

CUGAAGUGUUUGGAGGAACUU

>pab-miR396b MIMAT0018153

UUCCACGGCUUUCUUGAACUU

>pab-miR396c MIMAT0018154

UUCCACGGCUUUCUUGAACUU

>pab-miR397 MIMAT0018155

UCAUUGAGUGCAGCGUUGACG

>pab-miR482d MIMAT0018150

GGCUUGCGAGGAUAGGAAAAA

>pab-miR535 MIMAT0018159

UGACAACGAGAGAGAGCACGC

>pab-miR947 MIMAT0018160

UAUCGGAAUCUGUUACUGUUUC

>pab-miR950-3p MIMAT0018124

UCUGGGCCCCGGUGGUUUAUGA

>pab-miR950-5p MIMAT0018123

UCACAUCUGGGCCACGAUGGUU

>peu-miR2912a MIMAT0012879

UCUAGAACUCGAGAUAUGGGC

>peu-miR2912b MIMAT0012880

UCUAGAACUCGAGAUAUGGGC

>peu-miR2913 MIMAT0012881

GAGGUCGGGGAUUGCAAGGAG

>peu-miR2916 MIMAT0012884

UGGGGACUCGAAGACGAUCAUAU

>ppt-miR1215 MIMAT0003904

UCAUUGCAAAACUGUAUACGA

>ppt-miR1218 MIMAT0003907

CCUUAGAGUCGUAGGCCUCUG

>ppt-miR1219c MIMAT0003910

CUUCCUGCCUCUCACUAGCUU

>ppt-miR171b MIMAT0004376

UUGAGCCGCGCCAAUAUCACA

>ppt-miR319a MIMAT0003133

CUUGGACUGAAGGGAGCUCC

>ppt-miR319c MIMAT0003135

CUUGGACUGAAGGGAGCUCCC

>ppt-miR319d-3p MIMAT0003136

CUUGGACUGAAGGGAGCUCCC

>ppt-miR319d-5p.2 MIMAT0004326

UGGCUGAGUCGAAGGUUGUGC

>ppt-miR395 MIMAT0004355

CUGAAGCGUUUGGGGGAAGG

>ppt-miR408a MIMAT0004356

CUGCACUGCAUCUUCCCUGUGC

>ppt-miR414 MIMAT0004357

UCAUCCUCAUCAUCCUCGUCC

>ppt-miR419 MIMAT0004358

UGAUGAAUGAUGACGAUGUAU

>ppt-miR477c MIMAT0004359

CUCUCCCUCAAAGGCUUCCA

>ppt-miR477f MIMAT0004361

UCCCUCAAAGGCUUCCAACAA

>ppt-miR533b-5p MIMAT0003914

GAGCUGUCCAGGCUGUGAGGG

>ppt-miR534a MIMAT0003138

UAUGUCCAUUGCAGUUGCAUAC

>ppt-miR535b MIMAT0003140

UGACAACGAGAGAGAGCACGC

>ppt-miR535c MIMAT0003141

UGACAACGAGAGAGAGCACGC

>ppt-miR536a MIMAT0003144

UUCGUGCCAAGCUGUGUGCAAC

>ppt-miR536b MIMAT0004378

UUCGUGCCAAGCUGUGUGCAAC

>ppt-miR536c MIMAT0004374

AUCGUGCCAAGCUUUGUGCUUU

>ppt-miR537b MIMAT0003146

UUGAGGUGUUUCUACAGGCUA

>ppt-miR538a MIMAT0003147

UUGCAUGGAGUCUAUGUCUGGA

>ppt-miR893 MIMAT0004364

AUUGGGACUUGUGCUGGGAC

>ppt-miR894 MIMAT0004365

CGUUUCACGUCGGGUUCACC

>ppt-miR895 MIMAT0004366

GUAGCUUAGCGAGGUGUUGGUA

>ppt-miR897 MIMAT0004368

UGAUCAAGUGGAAACUCAGCAAA

>ppt-miR898b MIMAT0004381

UUGCUGUGCACUACUUAGUAC

>ppt-miR899 MIMAT0004369

AACUGAGAUACAUCGCAAUCG

>ppt-miR900-3p MIMAT0004370

UGUGUUCUUGUACCUGGGAAG

>ppt-miR901 MIMAT0004371

GGUAAAGUGGCGGCUAGGUUA

>ppt-miR902a-3p MIMAT0004372

ACGAAGGUCUGCAUCAUAGC

>ppt-miR902b-3p MIMAT0004373

ACGAAGGUCUGCAUCAUAGC

>ppt-miR903 MIMAT0004377

GCUACUUCGGCGGGACAAGAGC

>ppt-miR904a MIMAT0004379

UCUUGUCAAUGUUUAGGGGCA

>ppt-miR904b MIMAT0004380

UCUUGUCAAUGUUUAGGGGCA

>pta-miR156a MIMAT0004988

CAGAAGAUAGAGAGCACAUC

>pta-miR156b MIMAT0004989

CAGAAGAUAGAGAGCACAAC

>pta-miR159a MIMAT0004990

UUGGAUUGAAGGGAGCUCCA

>pta-miR159b MIMAT0004991

UUGGAUUGAAGAGAGCUCCC

>pta-miR319 MIMAT0004997

UUGGACUGAAGGGAGCUCC

>pta-miR946b MIMAT0005006

CAGCCCUUCUCCUAUCCACAA

>pta-miR947 MIMAT0005007

CAUCGGAAUCUGUUACUGUUUC

>pta-miR948 MIMAT0005008

UCAGGCUGUGUGGGAUCCGG

>pta-miR949 MIMAT0005009

UCUCCGGGAAUCCAAUGCGCC

>pta-miR950a MIMAT0005010

UCAGGUCCUCGGUGGUUUAU

>pta-miR950b MIMAT0005011

UCAGGUCCUCGGUGGUUUAU

>pta-miR951 MIMAT0005012

UGUUCUUGACGUCUGGACCAC

>pta-miR952b MIMAT0005014

AACUGAGAAUGCCAUUGGUG

>ptc-miR1444a MIMAT0005999

UCCACAUUCGGUCAAUGUUC

>ptc-miR1444b MIMAT0006000

UUCACAUUCGGUCAACGUUC

>ptc-miR1444c MIMAT0006001

UUCACAUUCGGUCAACGUUC

>ptc-miR1445 MIMAT0006002

UCCCUUGUAGACUAGAAAAA

>ptc-miR1446a MIMAT0006004

UUCUGAACUCUCUCCCUCAA

>ptc-miR1446b MIMAT0006005

UUCUGAACUCUCUCCCUCAA

>ptc-miR1446c MIMAT0006006

UUCUGAACUCUCUCCCUCAA

>ptc-miR1446e MIMAT0006008

UUCUGAACUCUCUCCCUCAA

>ptc-miR1449 MIMAT0006011

UGAGGUGCACGUAAGAUAACUC

>ptc-miR156h MIMAT0001897

UUGACAGAAGAUAGAGAGCAC

>ptc-miR156i MIMAT0001898

UUGACAGAAGAUAGAGAGCAC

>ptc-miR159b MIMAT0001902

UUUGGAUUGAAGGGAGCUCUA

>ptc-miR160b-5p MIMAT0001908

UGCCUGGCUCCCUGUAUGCCA

>ptc-miR164d MIMAT0001921

UGGAGAAGCAGGGCACGUGCA

>ptc-miR171e MIMAT0001987

UGAUUGAGCCGUGCCAAUAUC

>ptc-miR171f MIMAT0001988

UGAUUGAGCCGUGCCAAUAUC

>ptc-miR171h-3p MIMAT0001990

UGAUUGAGCCGUGCCAAUAUC

>ptc-miR171m MIMAT0005995

CGAGCCGAAUCAAUAUCACU

>ptc-miR172h-3p MIMAT0002000

GGAAUCUUGAUGAUGCUGCAG

>ptc-miR319a MIMAT0002002

UUGGACUGAAGGGAGCUCCC

>ptc-miR319b MIMAT0002003

UUGGACUGAAGGGAGCUCCC

>ptc-miR319c MIMAT0002004

UUGGACUGAAGGGAGCUCCC

>ptc-miR319d MIMAT0002005

UUGGACUGAAGGGAGCUCCC

>ptc-miR394a-3p MIMAT0006783

CUGUUGGUCUCUCUUUGUAA

>ptc-miR394b-3p MIMAT0006784

CUGUUGGUCUCUCUUUGUAA

>ptc-miR472a MIMAT0002060

UUUUCCCUACUCCACCCAUCCC

>ptc-miR474a MIMAT0002064

CAAAAGUUGCUGGGUUUGGCUGGG

>ptc-miR475a-3p MIMAT0002067

UUACAGUGCCCAUUGAUUAAG

>ptc-miR475b-3p MIMAT0002068

UUACAGUGCCCAUUGAUUAAG

>ptc-miR476a MIMAT0002071

UAGUAAUCCUUCUUUGCAAAG

>ptc-miR477b MIMAT0002075

AUCUCCCUCAGAGGCUUCCAA

>ptc-miR478a MIMAT0002076

UGACGUGUCUUCUAUUUUUAGGGA

>ptc-miR478b MIMAT0002077

UGACGUGUCUUCUAUUUUUAGGGA

>ptc-miR478c MIMAT0002078

UGACGUGUCUUCUAUUUUUAGGGA

>ptc-miR478d MIMAT0002079

UGACAUGUCUUCUAUUUUUAGUAA

>ptc-miR480 MIMAT0002096

ACUACUACAUCAUUGACGUUGAAC

>ptc-miR481a MIMAT0002098

AGGACCUCACUUAACAGCUUAAGC

>ptc-miR481b MIMAT0002099

AGGACCUCACUUAACAGCUUAAGC

>ptc-miR481c MIMAT0002100

AGGACCUCACUUAACAGCUUAAGC

>ptc-miR481d MIMAT0002102

AGGACCUCACCUAACAGCUUAAGC

>ptc-miR482a.1 MIMAT0002103

CCUACUCCUCCCAUUCC

>ptc-miR482a.2 MIMAT0006785

UCUUGCCUACUCCUCCCAUU

>ptc-miR530a MIMAT0005997

UGCAUUUGCACCUGCACCUU

>ptc-miR530b MIMAT0005998

UGCAUUUGCACCUGCAUCUU

>ptc-miR827 MIMAT0006003

UUAGAUGACCAUCAACGAAAA

>pvu-miR1514a MIMAT0011171

UUCAUUUUGAAAAUAGGCAUUG

>pvu-miR159a.1 MIMAT0015301

UUUGGAUUGAAGGGAGCUCUA

>pvu-miR159a.2 MIMAT0011170

CUUCCAUAUCUGGGGAGCUUC

>pvu-miR2118 MIMAT0011169

UUGCCGAUUCCACCCAUUCCUA

>pvu-miR2119 MIMAT0011174

UCAAAGGGAGUUGUAGGGGAA

>pvu-miR399a MIMAT0011177

UGCCAAAGGAGAGUUGCCCUG

>pvu-miR482-3p MIMAT0011173

UCUUCCCAAUUCCGCCCAUUCC

>rco-miR156c MIMAT0014146

UGACAGAAGAGAGUGAGCACA

>rco-miR156d MIMAT0014147

UGACAGAAGAGAGUGAGCACA

>rco-miR156e MIMAT0014148

UGACAGAAGAGAGAGAGCACA

>rco-miR160c MIMAT0014206

UGCCUGGCUCCCUGAAUGCCA

>rco-miR164d MIMAT0014159

UGGAGAAGCAGGGCACAUGCU

>rco-miR167c MIMAT0014167

UGAAGCUGCCAGCAUGAUCUGG

>rco-miR169b MIMAT0014170

CAGCCAAGGAUGACUUGCCGG

>rco-miR169c MIMAT0014171

UGAGCCAAGGAUGACUUGCCG

>rco-miR171c MIMAT0014174

UGAUUGAGCCGUGCCAAUAUC

>rco-miR171g MIMAT0014178

AGAUUGAGCCGCGCCAAUAUC

>rco-miR172 MIMAT0014179

GGAAUCUUGAUGAUGCUGCAG

>rco-miR319c MIMAT0014182

UUGGACUGAAGGGAGCUCCCU

>rco-miR319d MIMAT0014183

UUGGACUGAAGGGAGCUCCUU

>rco-miR395b MIMAT0014188

CUGAAGUGUUUGGGGGAACUC

>rco-miR395c MIMAT0014189

CUGAAGUGUUUGGGGGAACUC

>rco-miR397 MIMAT0014193

UCAUUGAGUGCAGCGUUGAUG

>rco-miR398a MIMAT0014194

UGUGUUCUCAGGUCACCCCUU

>rco-miR398b MIMAT0014195

UGUGUUCUCAGGUCGCCCCUG

>rco-miR399b MIMAT0014197

UGCCAAAGGAGAUUUGCCCGG

>rco-miR399c MIMAT0014198

UGCCAAAGGAGAUUUGCCCGG

>rco-miR399d MIMAT0014199

UGCCAAAGGAGAGCUGCCCUG

>rco-miR399e MIMAT0014200

UGCCAAAGGAGAUUUGCCCAG

>rco-miR399f MIMAT0014201

UGCCAAAGGAGAUUUGCUCAC

>rco-miR403a MIMAT0014202

UUAGAUUCACGCACAAACUCG

>rco-miR408 MIMAT0014204

CUGCACUGCCUCUUCCCUGGC

>sly-miR164a-5p MIMAT0033973

UGGAGAAGCAGGGCACGUGCA

>sly-miR164b-3p MIMAT0033976

CACGUGUUCUCCUUCUCCAAC

>tae-miR1117 MIMAT0005352

UAGUACCGGUUCGUGGCACGAACC

>tae-miR1118 MIMAT0005353

CACUACAUUAUGGAAUGGAGGGA

>tae-miR1119 MIMAT0005354

UGGCACGGCGUGAUGCUGAGUCAG

>tae-miR1120a MIMAT0005355

ACAUUCUUAUAUUAUGAGACGGAG

>tae-miR1121 MIMAT0005356

AGUAGUGAUCUAAACGCUCUUA

>tae-miR1122a MIMAT0005357

UAGAUACAUCCGUAUCUAGA

>tae-miR1123 MIMAT0005358

UCCGUGAGACCUGGUCUCAUAGA

>tae-miR1124 MIMAT0005359

GCAGGACGUGAAGAGCGAGUCC

>tae-miR1125 MIMAT0005360

AACCAACGAGACCAACUGCGGCGG

>tae-miR1127a MIMAT0005362

UCCUUCCGUUCGGAAUUAC

>tae-miR1128 MIMAT0005363

UACUACUCCCUCCGUCCGAAA

>tae-miR1129 MIMAT0005364

CAGCGAGCCAGCGGAGACCGGCAG

>tae-miR1130a MIMAT0005365

CCUCCGUCUCGUAAUGUAAGACG

>tae-miR1131 MIMAT0005366

UAGUACCGGUUCGUGGCUAACC

>tae-miR1133 MIMAT0005368

CAUAUACUCCCUCCGUCCGAAA

>tae-miR1134 MIMAT0005369

CAACAACAACAAGAAGAAGAAGAU

>tae-miR1135 MIMAT0005370

CUGCGACAAGUAAUUCCGAACGGA

>tae-miR1136 MIMAT0005371

UUGUCGCAGGUAUGGAUGUAUCUA

>tae-miR1137a MIMAT0005372

UAGUACAAAGUUGAGUCAUC

>tae-miR1138 MIMAT0005373

GCUUAGAUGUGACAUCCUUAAAA

>tae-miR1139 MIMAT0005374

AGAGUAACAUACACUAGUAACA

>tae-miR159b MIMAT0005344

UUUGGAUUGAAGGGAGCUCUG

>tae-miR160 MIMAT0005345

UGCCUGGCUCCCUGUAUGCCA

>tae-miR399 MIMAT0005349

UGCCAAAGGAGAAUUGCCC

>vun-miR169 MIMAT0022759

CAGCCAAGGAUGACUUGCCGG

>vun-miR319b MIMAT0022754

CUUGGACUGAAGGGAGCUCCU
