# Supplementary material for: A Comprehensive Prescription for Plant miRNA Identification
Source: Front Plant Sci. 2017 Jan 24;7:2058. doi: 10.3389/fpls.2016.02058 (PMC5258749; doi:10.3389/fpls.2016.02058)
Supplement: Supplementary file 3 [file Table3.PDF]

**Supplementary Document 3: Information about used dataset for pre-miRNA and mature miRNA/miRNA* duplex *in silico* expression analysis.**

For *in silico* expression analysis of putatively identified miRNAs, several data are collected from NCBI, ENA and DDJB databases. Identified miRNA precursors, mature miRNA and miRNA* sequences were aligned to these sequences and *in silico* expressed miRNAs were detected by filtration of results (Query identity and coverage: 95% for miRNA precursors, %100 for mature miRNA/miRNA* duplexes). In case of RNA sequencing data, reads were assembled into contigs with the help of Trinity software (https://github.com/trinityrnaseq/trinityrnaseq/wiki) and associated assembly metrics are also given in Table (1) and (2) including the related project ID. Table (3) and (4) contains the information of other sequences used for miRNA precursor expression analysis. Additionally, information about small RNA Seq. libraries used in *in silico* mature miRNA expression analysis was given in Table (5) and (6).

**(1) Transcriptomic data used for *B. distachyon* pre-miRNA expression analysis and associated assembly metrics**

| Transcriptome assemblies | Related project ID | # of assembled transcripts | Average contig length (b) | Total assembled bases (Mbp) |
|---|---|---|---|---|
| **Bd21-3** | DDJB: SRA171815 | 16,437 | 283.65 | 4.662308 |
| **Bd3-1** | DDJB: SRA171815 | 25,541 | 293.02 | 7.484015 |
| **Koz-3** | DDJB: SRA171815 | 20,482 | 287.16 | 5.881608 |
| **BdTR12C** | DDJB: SRA171815 | 19,946 | 286.58 | 5.716076 |
| **Bd30-1** | DDJB: SRA171815 | 25,178 | 292.56 | 7.365994 |
| **Bd1-1** | DDJB: | 19,932 | 286.74 | 5.715206 |

| | | | | |
|---|---|---|---|---|
| | SRA171815 | | | |
| **Bd21** | DDJB: SRA171815 | 23,350 | 290.71 | 6.788079 |
| **Bd1-1_Deep_RNASeq.** | NCBI: PRJNA253052 | 218,347 | 741.04 | 161.803097 |
| **ABR_5** | NCBI: PRJNA209327 | 29,343 | 487.69 | 14.310332 |
| **Bd21** | ENA: SRP008505 | 19,492 | 403.74 | 7.869703 |

**(2) Transcriptomic data used for *T. aestivum* pre-miRNA expression analysis and associated assembly metrics**

| Transcriptome assemblies | Related project ID | # of assembled transcripts | Average contig length (b) | Total assembled bases (Mbp) |
|---|---|---|---|---|
| Different tissues whole assembly (stem, spike, leaves, root, grain) | URGI, http://wheat-urgi.versailles.inra.fr/Seq-Repository/RNA-Seq | 389,276 | 687.07 | 267,460 |

**(3) Other sequences used for pre-miRNA expression analysis of *B. distachyon***

| Database name | Database version | # of sequences |
|---|---|---|
| **NCBI_EST** | GenBank , Release 214.0 | 207,717 |
| **NCBI_UniGene** | UniGene Build #2 | 113,694 |
| **PlantGDB PUT Assemblies** | PlantGDB,  Version 175a | 30,991 |
| **Phytozome-Bdi primary transcript data:** | Phytozome, Version 11 | 52,972 |

**(4) Other sequences used for pre-miRNA expression analysis of *T. aestivum***

| Database name | Database version | # of sequences |
|---|---|---|
| **NCBI_EST** | GenBank , Release 214.0 | 1,329,059 |

**(5) Small RNA sequencing libraries used in *in silico* mature miRNA/miRNA* expression analysis of *B. distachyon*.**

| Study ID | Related project ID |
|---|---|
| **Study1** | NCBI: PRJNA238902 |
| **Study2** | NCBI: PRJNA229162 |
| **Study3** | NCBI: PRJNA170607 |
| **Study4** | NCBI: PRJNA115065 |

**(6) Small RNA sequencing libraries used in *in silico* mature miRNA/miRNA* expression analysis of *T. aestivum*.**

| Study ID | Related project ID |
|---|---|
| **Study1** | NCBI: PRJNA275095 |
| **Study2** | NCBI: PRJNA115065 |
| **Study3** | NCBI: PRJNA232120 |
| **Study4** | NCBI: PRJNA289147 |
| **Study5** | NCBI: PRJNA142095 |
